# Nanoparticulate Perovskites for Photocatalytic Water Reduction

**DOI:** 10.3390/nano13142094

**Published:** 2023-07-18

**Authors:** Sven A. Freimann, Catherine E. Housecroft, Edwin C. Constable

**Affiliations:** Department of Chemistry, University of Basel, Mattenstrasse 22, BPR 1095, Postfach, 4002 Basel, Switzerlandcatherine.housecroft@unibas.ch (C.E.H.)

**Keywords:** nanoparticles, anchored catalyst, heterogeneous catalysis, water reduction

## Abstract

SrTiO_3_ and BaTiO_3_ nanoparticles (NPs) were activated using H_2_O_2_ or aqueous HNO_3_, and pristine and activated NPs were functionalized with a 2,2′-bipyridine phosphonic acid anchoring ligand (**1**), followed by reaction with RuCl_3_^.^3H_2_O and bpy, RhCl_3_^.^3H_2_O and bpy, or RuCl_3_^.^3H_2_O. The surface-bound metal complex functionalized NPs were used for the photogeneration of H_2_ from water, and their activity was compared to related systems using TiO_2_ NPs. The role of pH during surface complexation was found to be important. The NPs were characterized using Fourier transform infrared (FTIR) and solid-state absorption spectroscopies, thermogravimetric analysis mass spectrometry (TGA-MS), and matrix-assisted laser desorption/ionization mass spectrometry (MALDI-MS), and the dihydrogen generation was analyzed using gas chromatography–mass spectrometry (GC-MS). Our findings indicate that extensively functionalized SrTiO_3_ or BaTiO_3_ NPs may perform better than TiO_2_ NPs for water reduction.

## 1. Introduction

Increasing global economic development and a growing population have resulted in a higher demand for energy [1]. The burning of fossil fuels to provide energy releases greenhouse gases, which places immense stress on the environment. In the long term, this will cost society increasing amounts of resources [2,3,4,5]. As a consequence, research focusing on inexpensive energy solutions is increasingly focused on renewable and clean sources [6]. One solution is the use of dihydrogen derived from renewable sources as fuel or energy storage [7]. An attractive approach to a hydrogen economy is to combine energy harvesting and H_2_ evolution using photocatalysts operating under solar irradiation [8]. Hence, the design and development of efficient photocatalysts are crucial. A major contribution to this development is the use of heterogeneous catalysts [9,10,11,12]. These are often easier to recover than homogeneous catalysts but have the disadvantage of inactive interior volumes, with only surface sites being catalytically active [13,14,15]. An alternative is functionalizing nanoparticle (NP) scaffolds with photocatalysts. Such immobilized photocatalysts offer greater catalyst-to-volume ratios than bulk heterogeneous catalysts, and this enhances the catalytic activity and turnover. An additional benefit of NPs is the possibility of dispersing them in liquid phases [16,17,18,19].

In previous studies, we have shown that the binding of photo- and redox-active Rh and Ru coordination compounds onto TiO_2_ NP surfaces can be used successfully for H_2_ production, with the catalytic activity of the NP-supported catalyst outperforming previously reported, related homogeneous catalysts [20]. Another promising class of photocatalysts are perovskite-type oxides MTiO_3_ (M = Ca, Sr or Ba). These comprise cheap, earth-abundant elements [21,22,23], are water-insoluble, thermodynamically stable, and resistant to temperature- and photo-corrosion [24]. The photocatalytic properties of MTiO_3_ can be improved, tuned, and modified. This has been demonstrated using alternative synthetic methods [25,26], doping with organic or inorganic compounds [24,27,28], surface nanoparticle deposition [29,30], the use of cocatalysts [31,32], or the use of composites [33,34].

The photocatalytic properties of perovskites have been utilized for several applications, including organic compound degradation (methylene blue [24,25], methylene orange [29], salicylic acid [35], or ofloxacin dyes [34]) or H_2_ generation. In particular, SrTiO_3_ and BaTiO_3_ offer excellent activity in H_2_ generation. For example, the use of doped SrTiO_3_ has been widely demonstrated with examples, including Mn-, Ru-, Rh-, Ir- [27], Na- [36], Al-La- [37], and La-Rh- dopants [38]. Further approaches that result in improved H_2_ generation with doped SrTiO_3_ involve the introduction of oxygen vacancies [39] or additional cocatalysts, including Na-SrTiO_3_ combined with Rh_2-*y*_Cr*_y_*O_3_ [31] or Al-SrTiO_3_ with RhCrO*_x_* [32]. Similarly, BaTiO_3_ has been doped with Rh and functionalized with Pt nanoparticles to give a composite material shown to generate H_2_ upon irradiation [40]. A further promising feature of SrTiO_3_ and BaTiO_3_ NPs is their relatively similar pH-dependent zeta potentials and band gaps, only ~0.2 eV larger than TiO_2_ [41,42,43,44,45,46]. Functionalization using various anchoring groups, including phosphonic acids, carboxylic acids, or even hydroxy groups, makes them suitable candidates to extend our earlier results obtained with TiO_2_ NPs [47,48].

**Scheme 1:**
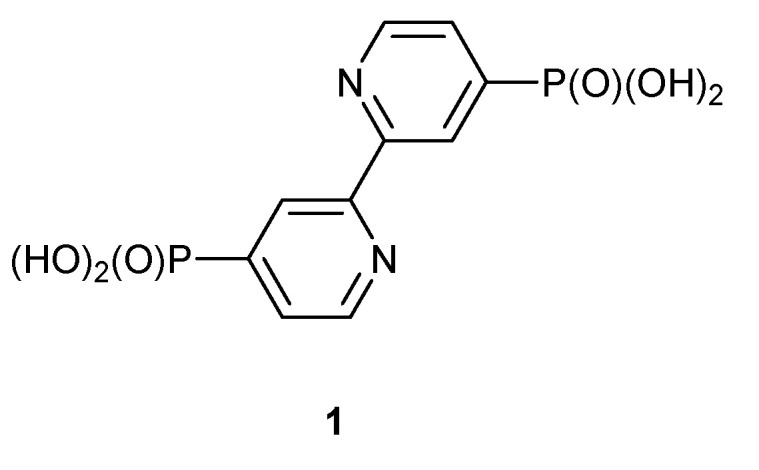
Anchoring ligand **1**.

In this work, we report the surface activation of pristine SrTiO_3_ and BaTiO_3_ NPs using either HNO_3_ or H_2_O_2_. The activated NPs are abbreviated as SrTiO_3_-a, BaTiO_3_-a (HNO_3_ activation) or SrTiO_3_-OH, BaTiO_3_-OH (H_2_O_2_ activation), respectively. We compare the subsequent functionalization of each type of pristine and activated NP with the ligand [2,2′-bipyridine]-4,4′-diylbis(phosphonic acid) (**1**, Scheme 1) which contains phosphonic acid anchoring domains. The functionalized NPs were used for direct surface metal complex assembly by reaction with 2,2′-bipyridine (bpy) and ruthenium or rhodium trichloride to give a surface-bound complex presumed to be (but not established as) an [M(bpy)_2_(**1**)]-species. The photocatalytic behavior of these metal-functionalized NPs was investigated. Experiments were also conducted to highlight the importance of pH control for successful metal complex assembly on the NP surface. The NPs were characterized using Fourier transform infrared (FTIR) and solid-state absorption spectroscopies, thermogravimetric analysis mass spectrometry (TGA-MS), and matrix-assisted laser desorption/ionization mass spectrometry (MALDI-MS), and the dihydrogen generation was analyzed using gas chromatography–mass spectrometry (GC-MS).

## 2. Materials and Methods

### 2.1. General

RuCl_3_^.^3H_2_O and RhCl_3_^.^3H_2_O were purchased from Oxkem Ltd. (Reading, UK) and Johnson Matthey (Materials Technology, Reading, UK). 2,2′-Bipyridine (bpy) and triethanolamine (TEOA) were purchased from Apollo Scientific Ltd. (Stockport, UK) and Sigma Aldrich Chemie GmbH (Buchs, Switzerland), respectively. K_2_[PtCl_4_] was purchased from Alfa Aesar GmbH & Co KG (Karlsruhe, Germany). Pristine SrTiO_3_ and BaTiO_3_ NPs were purchased from Sigma-Aldrich Chemie GmbH (Buchs, Switzerland) and had diameter sizes of <100 and 50 nm, respectively; further characterizations are reported in the Supplementary Materials. The anchoring ligand **1** was prepared as previously described [20]. Instrumentation details are given in the Supplementary Materials. The calculated major MALDI peaks reported in the Supplementary Materials were determined using the most abundant isotopes (e.g., ^102^Ru, ^35^Cl). For functionalized NPs in which the surface-bound ligand **1** is coordinated to a metal, a simplified notation is introduced; for example, **Ru**@SrTiO_3_ describes a SrTiO_3_ NP modified with ligand **1** which is also coordinated to Ru. In the case of NPs functionalized with both Ru and Rh, the relative amounts of the two metals are indicated using lower case (lower concentration) or upper case (higher concentration) letters (**r** or **R**). The first letter refers to Ru and the second to Rh, e.g., **rR**@SrTiO_3_ describes metal complex-functionalized NPs with Ru/Rh in a 1:20 molar ratio on the surface.

### 2.2. Experimental

#### 2.2.1. Nanoparticle Surface Activation Using HNO_3_ (SrTiO_3_-a, BaTiO_3_-a)

Pristine SrTiO_3_ or BaTiO_3_ NPs were activated as previously reported [20]. The NPs (2.00 g) were dispersed by sonication for 15 min in dilute aqueous HNO_3_ (30 mL, 3.0 M). The mixture was then stirred for 30 min. The suspension was centrifuged (10 min, 7000 rpm) and the NPs were washed once with milliQ water (40 mL). The NPs were added to milliQ water (40 mL) and dispersed by sonication for 10 min. The suspension was then stirred for 72 h. The suspension was centrifuged (10 min, 7000 rpm), and the NPs were washed with milliQ water (2 × 40 mL). The activated NPs (SrTiO_3_-a, 1.67 g and BaTiO_3_-a, 0.60 g) were stored in a sealed vial under N_2_ after drying them under high vacuum. The characterization data of the pristine NPs and acid-activated NPs are given in the Supplementary Materials. The TGA and TGA-MS spectra of the pristine NPs (Figures S1–S4) and acid-activated NPs (Figures S5–S7) are given in the Supplementary Materials.

#### 2.2.2. Nanoparticle Surface Activation Using H_2_O_2_ (SrTiO_3_-OH, BaTiO_3_-OH)

Pristine SrTiO_3_ or BaTiO_3_ NPs were activated as reported in the literature [49]. The NPs (1.05 g) were dispersed by sonication for 20 min in H_2_O_2_ (50 mL, 30%). The mixture was then stirred for 4 h at 110 °C under N_2_. The suspension was cooled down, centrifuged (10 min, 7000 rpm), and the NPs were washed with milliQ water (40 mL). The activated NPs (1.05 g and 1.05 g) were stored in a sealed vial under N_2_ after drying them under high vacuum for 72 h. The characterization data are given in the Supplementary Materials. The TGA and TGA-MS spectra of the H_2_O_2_-activated NPs (Figures S8–S11) are given in the Supplementary Materials.

#### 2.2.3. Nanoparticle Surface Ligand Functionalization (**1**@SrTiO_3_, **1**@SrTiO_3_-a, **1**@SrTiO_3_-OH, **1**@BaTiO_3_, **1**@BaTiO_3_-a, **1**@BaTiO_3_-OH)

The functionalization was performed as previously reported [20]. Anchoring ligand **1** (10.0 mg, 31.6 µmol, 1.0 eq.) and milliQ water (18 mL) were added to a microwave vial and dispersed by sonication for 1 min. Acid- or H_2_O_2_-activated NPs (449.0 mg, 4.6 SrTiO_3_ eq. or 7.3 BaTiO_3_) were added. The suspension was dispersed by sonication for 10 min. The microwave vial was sealed, and the reaction mixture was heated for 3 h at 130 °C in the microwave reactor. After cooling to room temperature, the suspension was centrifuged (20 min, 7000 rpm). The NPs were separated from the solvent and washed with EtOH (2 × 15 mL). This procedure gave white f-NPs (f-NP = functionalized NP) with the following yields: **1**@SrTiO_3_-a NPs (418.3 mg), **1**@SrTiO_3_-OH NPs (422.6 mg), **1**@BaTiO_3_-a NPs (409.9 mg), **1**@BaTiO_3_-OH NPs (425.1 mg), and these were stored in a sealed vial under N_2_ after drying the NPs under high vacuum. This reaction was repeated using pristine SrTiO_3_ and BaTiO_3_ NPs (224 mg, 4.6 SrTiO_3_ eq. or 7.3 BaTiO_3_ eq.) and **1** (5.0 mg, 15.8 µmol) yielding **1**@SrTiO_3_ NPs (218.1 mg, 221.6 mg) and **1**@BaTiO_3_ NPs (210.7 mg, 219.4 mg). For NMR spectroscopic measurements, NPs (5–10 mg) were dispersed in 500 µL D_2_O in an NMR tube. The characterization data are given in the Supplementary Materials. The TGA, TGA-MS, and MALDI spectra of the pristine (Figures S12–S17), acid-activated (Figures S18–S22), and H_2_O_2_-activated (Figures S23–S28) functionalized NPs are given in the Supplementary Materials.

#### 2.2.4. Nanoparticle Surface Complexation (**Ru**@SrTiO_3_, **Ru**@BaTiO_3_)

The metal complex was formed directly on the NP surface. **1**@SrTiO_3_ (70.9 mg), RuCl_3_^.^3H_2_O (1.03 mg, 3.94 µmol), and bpy (1.25 mg, 8.00 µmol) were added to a vial. H_2_O (5.0 mL) and EtOH (3.0 mL) were added, and the mixture was thoroughly dispersed using sonication and stirring. The suspension was transferred into an autoclave PTFE liner with additional EtOH (2.0 mL). The autoclave was sealed and then heated in an oven at a heating rate of 320 °C/h to 160 °C. The autoclave was left at 160 °C for 1 h. After cooling, the autoclave was opened, and the suspension was centrifuged (20 min, 7000 rpm). The resulting NPs were washed with H_2_O (3 × 10 mL) and EtOH (1 × 10 mL). **Ru**@SrTiO_3_ (65.8 mg) was obtained as a pale orange powder after drying the NPs under a high vacuum. This experiment was repeated with **1**@BaTiO_3_ (70.9 mg), RuCl_3_^.^3H_2_O (1.03 mg, 3.95 µmol), and bpy (1.25 mg, 8.00 µmol) yielding **Ru**@BaTiO_3_ (67.0 mg) as a dark brown powder. The characterization data are given in the Supplementary Materials. The TGA, TGA-MS, and MALDI spectra are given in the Supplementary Materials (Figures S29–S34).

#### 2.2.5. Nanoparticle Surface Complexation (**rR**@SrTiO_3_, **rR**@SrTiO_3_-a, **rR**@SrTiO_3_-OH, **rR**@SrTiO_3_-OH-A, **rR**@BaTiO_3_, **rR**@BaTiO_3_-a, **rR**@BaTiO_3_-OH, **rR**@BaTiO_3_-OH-A)

The notation is defined in Section 2.1. The metal complex was formed directly on the NP surface. This reaction was carried out with each isolated NP from Section 2.2.1 and Section 2.2.2. Hence, **1**@SrTiO_3_, **1**@SrTiO_3_-a NPs, **1**@SrTiO_3_-OH, **1**@BaTiO_3_, **1**@BaTiO_3_-a, or **1**@BaTiO_3_-OH (365.6 mg) was added to a vial with RuCl_3_^.^3H_2_O (0.24 mg, 0.9 µmol), RhCl_3_^.^3H_2_O (5.15 mg, 19.6 µmol), and bpy (6.45 mg, 41.3 µmol). H_2_O (5.0 mL) and EtOH (3.0 mL) were added, and the mixture was thoroughly dispersed using sonication and stirring. The suspension was transferred to an autoclave with a PTFE liner with additional EtOH (2.0 mL). The autoclave was sealed and then heated in an oven at a rate of 320 °C/h to 160 °C. The autoclave was left at 160 °C for 1 h. After cooling, the autoclave was opened, and the suspension was centrifuged (20 min, 7000 rpm). The resulting NPs were washed with H_2_O (3 × 10 mL) and EtOH (1 × 10 mL). After drying the NPs under high vacuum, the reaction yielded **rR**@SrTiO_3_ (354.2 mg) as a pale orange powder, **rR**@SrTiO_3_-a (358.2 mg) as a pale orange powder, **rR**@SrTiO_3_-OH (358.4 mg) as a grey powder, **rR**@BaTiO_3_ (349.9 mg) as a grey powder, **rR**@BaTiO_3_-a (356.9 mg) as a dark brown powder or **rR**@BaTiO_3_-OH (357.4 mg) as a dark grey powder. This reaction was repeated with **1**@SrTiO_3_-OH and **1**@BaTiO_3_-OH with a pH adjusted to 1.5 using 4.0 mL H_2_O, 1.0 mL aqueous H_2_SO_4_ (1 M), and the same amount of EtOH (5 mL). Other reaction conditions were kept the same. **rR**@SrTiO_3_-OH-A (356.0 mg) and **rR**@BaTiO_3_-OH-A (355.1 mg) were both isolated as a pale orange powder. The characterization data are given in the Supplementary Materials. The TGA, TGA-MS, and MALDI spectra of the pristine (Figures S36–S40), acid-activated (Figures S41–S45), H_2_O_2_-activated (Figures S46–S51), and H_2_O_2_-activated under adjusted pH (Figures S52–S55) functionalized, and ruthenium-, rhodium-, and bpy-complexed NPs are given in the Supplementary Materials. An FTIR reference spectrum (Figure S56) was recorded by mixing pristine SrTiO_3_ NPs (500 mg) with aqueous H_2_SO_4_ (200 µL, 3 M) using a mortar.

#### 2.2.6. Dihydrogen Generation

The water reduction reaction conditions were consistent with the conditions in the previous work with TiO_2_ NPs [20]. Triethanolamine (TEOA) was added as a sacrificial electron donor, K_2_[PtCl_4_] as a catalyst to facilitate H_2_ formation (feasibly by the formation of Pt nanoparticles), bpy as an additive, aqueous H_2_SO_4_ for altering the solution pH. TEOA (2.52 mmol, 376 mg), K_2_[PtCl_4_] (1.7 µmol, 0.70 mg), and bpy (18.6 µmol, 2.91 mg) were added to a 5 mL microwave vial with milliQ water and aqueous H_2_SO_4_ (1 M) to modify the pH. The experiments were performed at pH 7.5 and used 1 mL aqueous H_2_SO_4_ (1 M) and 5 mL milliQ water. Metal complex-functionalized NPs were added (114.1 mg). The vial was flushed with N_2_ and then sealed. The suspension was sonicated (10 min) and thoroughly shaken. N_2_ was bubbled through the suspension for 10 min. The vial was irradiated using a sun simulator generating 1200 W m^−2^ (see the Supplementary Materials) for 4 h with an incident angle of light of 5°. The suspension was stirred throughout the irradiation and periodically shaken. Headspace samples for gas chromatography were collected using a syringe and transferred to a 10 mL GC vial for analysis. The measured GC integral was converted into mL of H_2_ with calibration performed by injecting several known volumes of H_2_.

## 3. Results and Discussion

### 3.1. NP Activation, Functionalization, Surface Complexation, and Material Characterization

#### 3.1.1. NP Surface Activation

The surface activation of NPs is a crucial step for successful and stable surface functionalization. We previously described the benefits of acid activation of TiO_2_ NPs for their use in catalysis [20]. However, in the literature, other methods are also described, including using various acids (e.g., MeCO_2_H or HF), hydrogen peroxide, or plasma for surface activation [49,50,51]. These methods are used to enhance the surface reactivity and make the surface more reactive for functionalization. TiO_2_ and SrTiO_3_ NPs are acid-resistant, whereas BaTiO_3_ NPs are sensitive to mineral acids. Hence, as described in Section 2.2.1, during acid activation with aqueous HNO_3_ (3 M), the BaTiO_3_ NPs partially dissolved or were lost during washing (70%). Since acid activation was unsuitable for the BaTiO_3_ NPs, other activation methods were explored, and activation using the H_2_O_2_ treatment [49] was chosen. This method simultaneously saturates the surface with hydroxyl groups and strips it of carbonate groups. Although the literature protocol required hydroxyl groups for a salinization reaction [49], the hydroxyl groups should also favor condensation with the phosphonic acid of the anchoring ligand.

**Figure 1:**
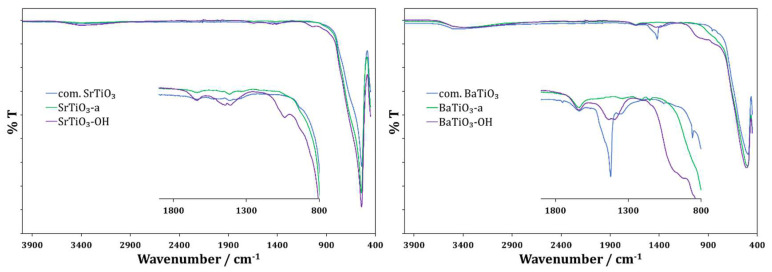
FTIR spectra of pristine SrTiO_3_ NPs (**left**, blue), SrTiO_3_-a NPs (**left**, green), SrTiO_3_-OH NPs (**left**, violet); pristine BaTiO_3_ (**right**, blue), BaTiO_3_-a NPs (**right**, green), BaTiO_3_-OH NPs (**right**, violet).

Activation was performed with both SrTiO_3_ and BaTiO_3_ by boiling the dispersed NPs under N_2_ in 30% H_2_O_2_. The particles were washed with water and then dried for 72 h under high vacuum. The pristine and activated NPs were characterized using FTIR and TGA-MS. The FTIR differences shown in Figure 1 were limited to the fingerprint region. For the pristine SrTiO_3_ activated with H_2_O_2_, an additional absorption at 1446 cm^−1^ appeared. The pristine BaTiO_3_ activated with acid showed the disappearance of a prominent peak at 1420 cm^−1^, while the activation with H_2_O_2_ caused the peak to shift and broaden.

The TGA-MS results are reported in Table 1. A comparison of the pristine SrTiO_3_ and BaTiO_3_ NPs with acid-activated NPs (SrTiO_3_ and BaTiO_3_) shows that there was a slight increase in the weight loss (0.1–0.4%) in the lower temperature region (<380 °C). The NPs activated with H_2_O_2_ showed a greater weight loss than the pristine NPs, with an increase in the lower temperature region of 1.2% or 2.2% for SrTiO_3_ and BaTiO_3_. Using TGA-MS, a peak with amu 18 (H_2_O) was found in each sample and assigned to physisorbed and chemisorbed water. For H_2_O_2_-activated NPs, this increase could also be due to the loss of the hydroxyl groups. The pristine NPs and H_2_O_2_-activated NPs showed organic decomposition products (amu 44, CO_2_) throughout the TGA experiment, possibly due to impurities in the pristine samples; this was especially visible for BaTiO_3_.

**Figure 2:**
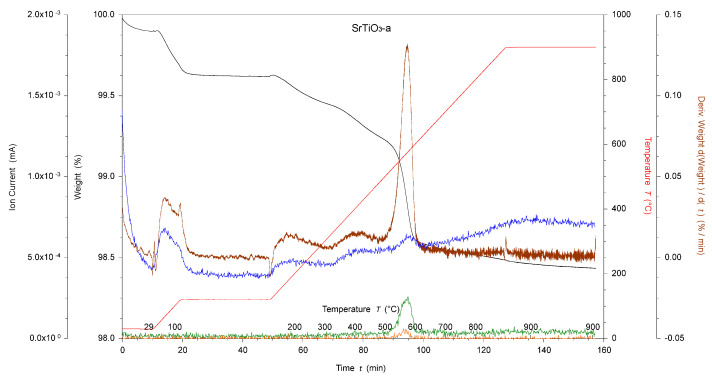
TGA-MS of acid-activated SrTiO_3_ NPs. SrTiO_3_-a, where black is the weight loss, red is the temperature, brown is the derivative weight against time, blue is the ion current of amu 18, green is the ion current of amu 44, and orange is the ion current of amu 81.

In the higher temperature region (380–900 °C), H_2_O_2_-activated SrTiO_3_ and BaTiO_3_ NPs also showed slightly higher weight losses (0.2%) than the pristine NPs. The acid-activated SrTiO_3_ and BaTiO_3_ NPs showed slightly higher (0.6%) and lower (0.1%) weight losses, respectively. In both cases, the TGA-MS experiment recorded a significant weight loss at ~550 °C attributed to amu 44 (CO_2_), and, in the case of SrTiO_3_-a, a mass loss at amu 81 was also observed. The TGA-MS experiment on SrTiO_3_-a is illustrated in Figure 2 as an example of the sharp weight loss. The origin of this weight loss is unclear.

#### 3.1.2. Nanoparticle Surface Ligand Functionalization

**Figure 3:**
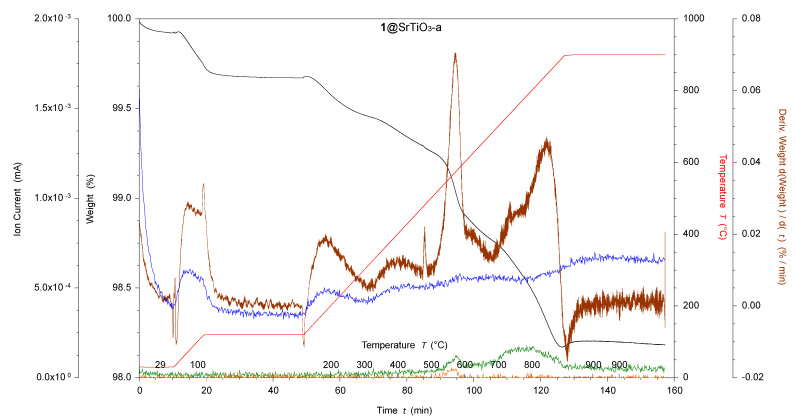
TGA-MS of acid-activated with ligand **1**-functionalized SrTiO_3_ NPs. **1**@SrTiO_3_-a, where black is the weight loss, red is the temperature, brown is the derivative weight against time, blue is the ion current of amu 18, green is the ion current of amu 44, and orange is the ion current of amu 81.

The bpy metal-binding domain in ligand **1** (Scheme 1) was chosen for the surface assembly of {M(bpy)_3_}*^n^*^+^ (M = Ru, *n* = 2; M = Rh, *n* = 3) moieties. Phosphonic acids bind strongly to BaTiO_3_ NP surfaces [47], and it is reasonable to assume a similar behavior with SrTiO_3_ NPs. The pristine BaTiO_3_ and SrTiO_3_ NPs had 50 and 100 nm diameters, respectively, making them larger than the p25 TiO_2_ NPs used in previous work [20,52,53]. This difference significantly changes the surface area from a volume ratio of 0.28 nm^−1^ for TiO_2_ to 0.12 nm^−1^ and 0.06 nm^−1^ for BaTiO_3_ and SrTiO_3_, respectively. This reduces the available surface area and necessitates functionalization adjustments to avoid the free ligand’s physisorption. Section 2.2.3 details the functionalization methods adopted. The NPs were characterized using ^1^H NMR spectroscopy, FTIR, TGA-MS, MALDI-MS, and solid-state absorption spectroscopy. The TGA-MS results are presented in Table 2 and show higher weight losses than the corresponding unfunctionalized NPs in the high-temperature region (380–900 °C), indicating successful surface functionalization. When compared to TiO_2,_ the weight loss due to functionalization was smaller (0.2–0.7% versus 2.6%) [20] than expected since the SrTiO_3_ and BaTiO_3_ NPs had considerably larger particle sizes. The NPs all showed carbon-containing impurities before functionalization, as described in Section 3.1.1. Based on the TGA-MS experiments in the low-temperature region, the impurities were lost during the functionalization. Figure 3 shows the TGA-MS measurement of **1**@SrTiO_3_-a, where ligand **1** was decomposed in a single event distinct from the decompositions recorded in the starting material. The TGA-MS measurement of **1**@SrTiO_3_ still shows the peak observed for SrTiO_3_-a at ~550 °C with an additional weight loss between 700 °C and 880 °C. The TGA-MS in this region shows amu 18 (H_2_O) and 44 (CO_2_), corresponding to a decomposition of the anchoring ligand **1**.

**Figure 4:**
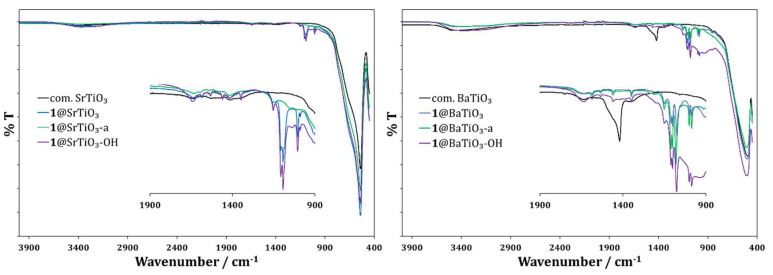
FTIR spectra of pristine SrTiO_3_ (**left**, black), **1**@SrTiO_3_ NPs (**left**, blue), **1**@SrTiO_3_-a NPs (**left**, green), **1**@SrTiO_3_-OH NPs (**left**, violet); pristine BaTiO_3_ (**right**, black), **1**@BaTiO_3_ NPs (**right**, blue), **1**@BaTiO_3_-a NPs (**right**, green), **1**@BaTiO_3_-OH NPs (**right**, violet).

The FTIR spectra of the f-NPs (Figure 4) showed a broad, weak absorption around 3300 cm^−1^, in accordance with the TGA-MS results, supporting the presence of hydroxy groups. The NPs also exhibited a strong absorption at 540 cm^−1^ and 500 cm^−1^ for SrTiO_3_ and BaTiO_3_, respectively. Compared to the unfunctionalized NPs, **1**@SrTiO_3_ and **1**@BaTiO_3_ (independent of prior activation) showed several absorptions in the range 1900 cm^−1^ to 900 cm^−1^, indicating a bound ligand. The possibility of traces of an absorbed and labile species being on the NP surface after the functionalization was excluded based on the ^1^H NMR spectroscopy.

**Figure 5:**
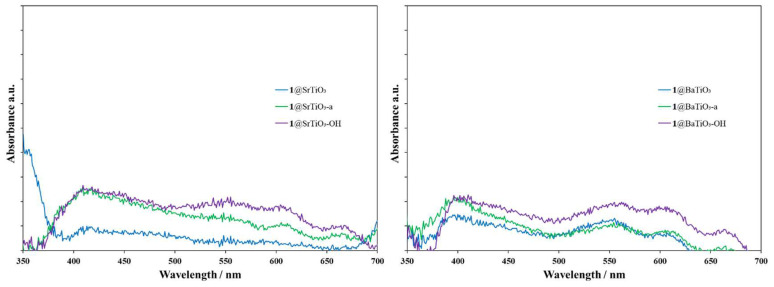
Solid-state absorption spectra of **1**@SrTiO_3_ NPs (**left**, blue), **1**@SrTiO_3_-a NPs (**left**, green), **1**@SrTiO_3_-OH NPs (**left**, violet); **1**@BaTiO_3_ NPs (**right**, blue), **1**@BaTiO_3_-a NPs (**right**, green), **1**@BaTiO_3_-OH NPs (**right**, violet).

The solid-state absorption spectra used the pristine NPs (SrTiO_3_ or BaTiO_3_) as the 100% baseline, and the results are shown in Figure 5. The f-NPs showed a broad weak absorption between 400 and 700 nm. These results are similar to the solid-state absorption spectra of the ligand **1**-functionalized TiO_2_ NPs [20].

#### 3.1.3. Nanoparticle Surface Complexation

**Figure 6:**
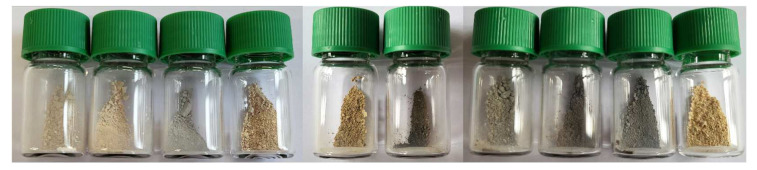
Isolated f-NPs from left to right: **rR**@SrTiO_3_, **rR**@SrTiO_3_-a, **rR**@SrTiO_3_-OH, **rR**@SrTiO_3_-OH-A, **Ru**@SrTiO_3_, **Ru**@BaTiO_3_, **rR**@BaTiO_3_, **rR**@BaTiO_3_-a, **rR**@BaTiO_3_-OH, **rR**@BaTiO_3_-OH-A.

For all complexations, the f-NPs were dispersed in an autoclave in a mixture of H_2_O/EtOH together with RuCl_3_**^.^**3H_2_O, RhCl_3_**^.^**3H_2_O, and bpy (see Section 2.2.4 and Section 2.2.5 for detailed procedures). Depending on the activation of the NPs and the pH during the complexation, differently colored NPs with different catalytic activities were isolated. The ruthenium and rhodium metal complex-bearing NPs were tested for their ability to catalyze dihydrogen production from water under irradiation using simulated sunlight. The method was also utilized with only RuCl_3_^.^3H_2_O to prepare **Ru**@SrTiO_3_ and **Ru**@BaTiO_3_. The isolated f-NPs are shown in Figure 6 and were characterized using ^1^H NMR spectroscopy, FTIR, TGA-MS, MALDI-MS, and solid-state absorption spectroscopy.

TGA-MS revealed an increased weight loss of the complex-bearing NPs when comparing them to ligand-functionalized NPs. The results are shown in Table 3. The greater weight loss for the metal complexed f-NPs compared to the ligand f-NPs in the high-temperature region (380–900 °C) was rather low (0.2–0.5%). This provides evidence for the low degree of functionalization compared to the equivalent functionalized TiO_2_ NPs [20].

**Figure 7:**
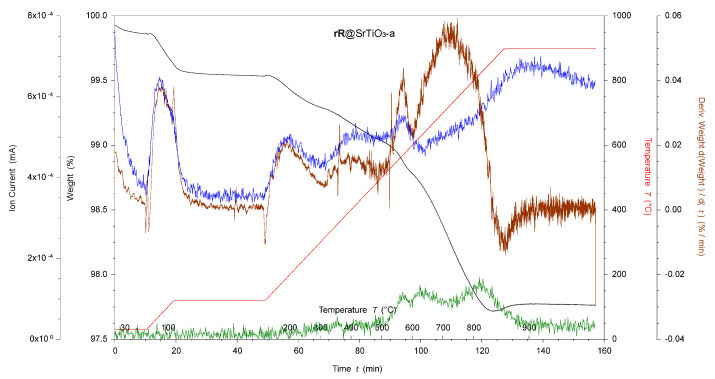
TGA-MS of acid-activated with ligand **1**-functionalized and ruthenium-, rhodium-, and bpy-complexed SrTiO_3_ NPs. **rR**@SrTiO_3_-a, where black is the weight loss, red is the temperature, brown is the derivative weight against time, blue is the ion current of amu 18, and green is the ion current of amu 44.

A successful complexation was expected to result in additional decomposition in the TGA-MS. Hence, the TGA-MS data of SrTiO_3_-a (Figure 2) and **1**@SrTiO_3_-a (Figure 3) to **rR**@SrTiO_3_-a (Figure 7) were used to identify the decomposition processes. Using the ion current of CO_2_, amu 44 (Figure 7, green), the decompositions can be differentiated from the starting material. The decomposition of **rR**@SrTiO_3_-a occurred at a slightly lower temperature (~650 °C) than the recorded decomposition for **1**@SrTiO_3_-a (~800 °C) and higher than the impurity recorded with SrTiO_3_-a (~550 °C).

**Figure 8:**
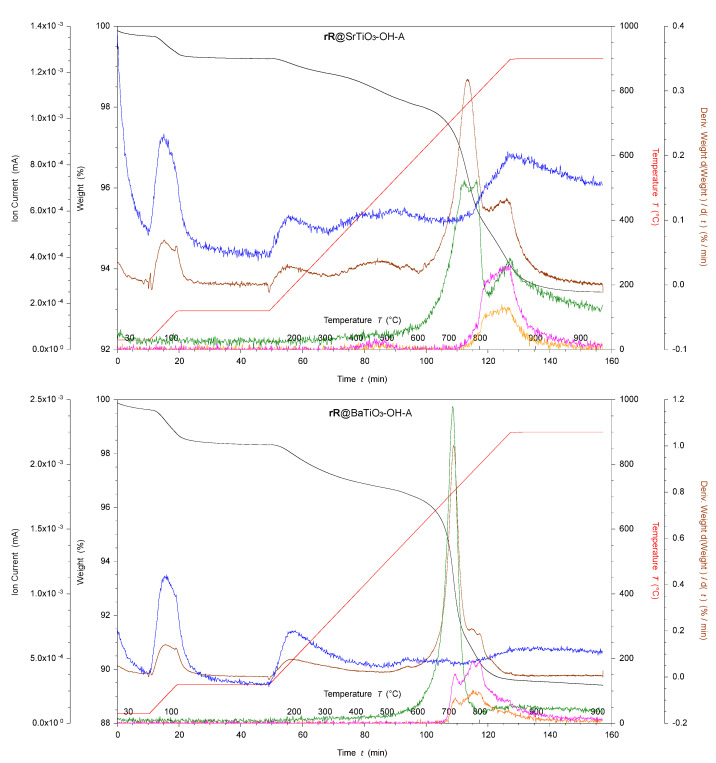
TGA-MS of H_2_O_2_ activated with ligand **1**-functionalized and ruthenium, rhodium, and bpy under adjusted pH complexed SrTiO_3_ NPs (**top**) and BaTiO_3_ NPs (**bottom**). **rR**@SrTiO_3_-OH-A and **rR**@SrTiO_3_-OH-A, where black is the weight loss, red is the temperature, brown is the derivative weight against time, blue is the ion current of amu 18, green is the ion current of amu 44, yellow is the ion current of amu 48, and pink is the ion current of amu 64.

In further experiments with **1**@SrTiO_3_-OH and **1**@BaTiO_3_-OH, the pH for the complexation reaction in the autoclave was adjusted from 6.9 to 1.5 with aqueous H_2_SO_4_. The resulting NPs showed large weight loss increases in the high-temperature region of 4.0% (Figure 8, bottom, labeled **rR**@SrTiO_3_-OH-A) and 6.1% (Figure 8, top, **rR**@BaTiO_3_-OH-A). However, the TGA-MS showed peaks at amu 48 (SO) and 64 (SO_2_) at 750 °C, suggesting the surface binding of H_2_SO_4_ [54]. The presence of amu 44 (CO_2_, Figure 8) suggests an organic decomposition, indicating that the decomposition of H_2_SO_4_ was not the main cause of the weight loss. The main weight loss occurred at ~700 °C, corresponding to the decomposition also observed for **rR**@SrTiO_3_-a. The earlier decomposition at ~550 °C and ~800 °C was not observed for **rR**@SrTiO_3_-OH-A. For **rR**@BaTiO_3_-OH-A, an additional decomposition at ~900 °C was observed. ^1^H NMR spectroscopy was used to verify the absence of a non-bound anchoring ligand in all cases.

**Figure 9:**
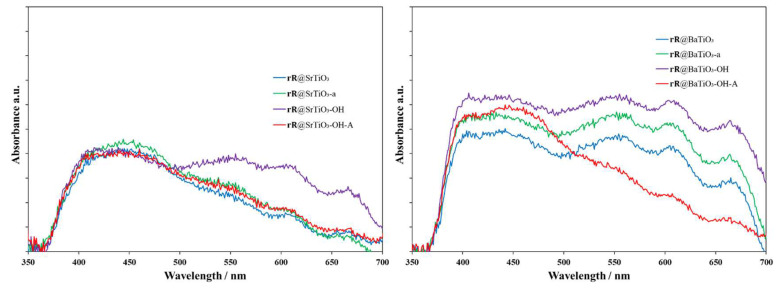
Solid-state absorption spectra of **rR**@SrTiO_3_ NPs (**left**, blue), **rR**@SrTiO_3_-a NPs (**left**, green), **rR**@SrTiO_3_-OH NPs (**left**, violet), **rR**@SrTiO_3_-OH-A NPs (**left**, red); **rR**@BaTiO_3_ NPs (**right**, blue), **rR**@BaTiO_3_-a NPs (**right**, green), **rR**@BaTiO_3_-OH NPs (**right**, violet), **rR**@BaTiO_3_-OH-A NPs (**right**, red).

The solid-state absorption spectra of the complexed NPs are shown in Figure 9. In the case of SrTiO_3_, the spectrum of the metal complex f-NPs (Figure 9, left) shows a broad absorption between 410 nm and 480 nm, with weaker absorptions between 500 and 700 nm. These generally agree with the absorption spectra for the corresponding TiO_2_ NPs [20]. However, **rR**@SrTiO_3_-OH NPs (Figure 9, left, violet) exhibited more intense absorptions in the regions 540–570, 600–620, and 660–680 nm. As these NPs were grey to black, a panchromatic absorption was to be expected. Metal complex f-NPs with BaTiO_3_ NPs (Figure 9, right) showed similar panchromatic adsorption. For **rR**@BaTiO_3_-OH-A (Figure 9, right, red), absorptions between 600–620 and 660–680 nm were less intense than other NPs, in accordance with their pale orange color.

**Figure 10:**
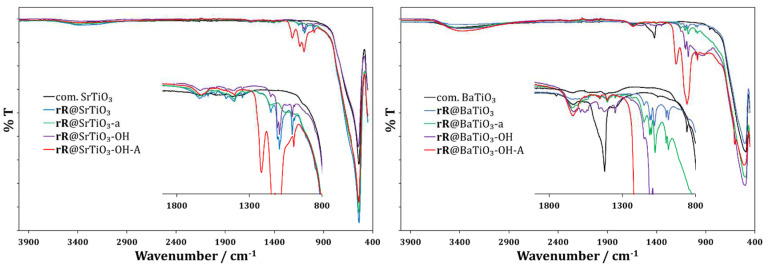
FTIR spectra of **rR**@SrTiO_3_ NPs (**left**, blue), **rR**@SrTiO_3_-a NPs (**left**, green), **rR**@SrTiO_3_-OH NPs (**left**, violet), **rR**@SrTiO_3_-OH-A NPs (**left**, red); **rR**@BaTiO_3_ NPs (**right**, blue), **rR**@BaTiO_3_-a NPs (**right**, green), **rR**@BaTiO_3_-OH NPs (**right**, violet), **rR**@BaTiO_3_-OH-A NPs (**right**, red).

Figure 10 shows the FTIR spectra of **rR**@SrTiO_3_ (left, blue) and **rR**@BaTiO_3_ (right, blue) and their variants (-a, green; -OH, violet; -OH-A, red). Overall, the FTIR spectra of all metal complex f-NPs of SrTiO_3_ and BaTiO_3_ were similar (except for **rR**@SrTiO_3_-OH-A and **rR**@BaTiO_3_-OH-A). In addition, the spectra were very similar to that of the anchoring ligand f-NPs described in Section 3.1.2, with only small differences in the fingerprint region (1900 to 900 cm^−1^). In contrast, the FTIR spectra of **rR**@SrTiO_3_-OH-A and **rR**@BaTiO_3_-OH-A showed strong absorptions at 1219, 1141, and 1100 and at 1200 and 1091 cm^−1^, respectively, due to the presence of H_2_SO_4_ on the NP surface. This was confirmed by recording an FTIR spectrum of a mixture of pristine SrTiO_3_ NPs and aqueous H_2_SO_4_ (see Supplementary Materials, Figure S56).

As expected, attempts to quantify the ruthenium or rhodium content of the functionalized NPs using energy-dispersive X-ray (EDX) spectroscopy were unsuccessful, as the expected values were below the 1% detection limit.

### 3.2. Dihydrogen Generation from Water

#### General Procedure

The experimental details of these studies are given in Section 2.2.6, and the results are collected in Table 4. The f-NPs that produced dihydrogen showed the characteristic red color of a {Ru(bpy)_3_}^2+^ chromophore, while f-NPs that were either grey or black either did not produce H_2_ or did so with low efficiency. BaTiO_3_ NPs and SrTiO_3_ NPs activated with H_2_O_2_, functionalized with ligand **1** and then complexed with RuCl_3_^.^3H_2_O and RhCl_3_^.^3H_2_O were less active for water reduction. This indicates minimal or no formation of the required photocatalyst(s) on the surface.

As discussed in our previous paper [20], surface functionalization is increased by the HNO_3_ treatment, leading to more active catalytic sites on the surface and greater hydrogen production.

## 4. Conclusions

We explored activation methods for SrTiO_3_ and BaTiO_3_ NPs using H_2_O_2_ and aqueous HNO_3_. Pristine and activated SrTiO_3_ and BaTiO_3_ NPs were functionalized with anchoring ligand **1** and subsequently elaborated using RuCl_3_^.^3H_2_O and bpy, RhCl_3_^.^3H_2_O and bpy, or RuCl_3_^.^3H_2_O. The potentially photo- and redox-active Rh and Ru surface-bound metal complex f-NPs were used in a photochemical system for the solar generation of H_2_ from water. BaTiO_3_ NPs and SrTiO_3_ NPs activated with H_2_O_2_, functionalized with ligand **1**, and then complexed with RuCl_3_^.^3H_2_O and RhCl_3_^.^3H_2_O yielded f-NPs that were inactive for water reduction. **rR**@SrTiO_3_-a was the most efficient within our tested materials, giving 1.1 mL H_2_ per hour, with **rR**@SrTiO_3_ being roughly two-thirds as efficient. The complexation of the metal species to the H_2_O_2_-activated and ligand-functionalized NPs was modified by adjusting the pH to 1.5 with aqueous H_2_SO_4_. The resulting orange SrTiO_3_ and BaTiO_3_ NPs were active for water reduction and produced H_2_. **rR**@SrTiO_3_-OH-A were considerably less active than **rR**@SrTiO_3_ or **rR**@SrTiO_3_-a, while **rR**@BaTiO_3_-OH-A performed better than **rR**@BaTiO_3_-a. Hence, pH seems to be more important during complexation than previously thought, and adjusting it can play a major role. It is unclear if the drop in efficiency for **rR**@SrTiO_3_-OH-A was due to residual H_2_SO_4_ influencing the pH of the suspension during the water reduction or if complexation was impacted. For **rR**@BaTiO_3_-OH-A, the solid-state absorption spectroscopic data and the red color of the functionalized NPs further suggest the formation of surface-bound {Ru(bpy)_3_}^2+^ chromophores.

We conclude that extensively functionalized SrTiO_3_ or BaTiO_3_ NPs may perform better than TiO_2_ NPs for water reduction. However, the former NPs are more expensive than TiO_2_, and cost-benefit and scale-up limitations should be explored. Particle size might also play a significant role in surface loading, and we note that the pristine BaTiO_3_ and SrTiO_3_ NPs had 2 to 4 times larger radii than the TiO_2_ NPs. This work included the characterization of activated f-NPs and metal complex f-NPs using FTIR spectroscopy, solid-state absorption spectroscopy, and TGA-MS, providing evidence for successful functionalization.

## Figures and Tables

**Table 1:** TGA-MS results of non-activated and activated SrTiO_3_ and BaTiO_3_ NPs. (-a) represents HNO_3_-activated NPs and (-OH) represents H_2_O_2_-activated NPs.

Entry	30–380 °C /%	Mass Found /amu	380–900 °C /%	Mass Found /amu
Pristine SrTiO_3_	0.5	18; 44 ^a^	0.4	18; 44
SrTiO_3_-a	0.6	18	1.0	18, 44 ^b^, 81 ^b^
SrTiO_3_-OH	1.8	18; 44	0.6	18; 44
PristineBaTiO_3_	1.0	18; 44	0.6	18; 44
BaTiO_3_-a	1.4	18	0.5	18; 44 ^b^
BaTiO_3_-OH	3.6	18; 44	0.8	18; 44

^a^ Peak with amu 44 was recorded as traces slightly above baseline. ^b^ Peak with amu 44 was only recorded within a temperature range of 500–600 °C.

**Table 2:** TGA-MS results of **1**@SrTiO_3_ and **1**@BaTiO_3_ NPs using non-activated and activated NPs.

Entry	30–380 °C /%	Mass Found /amu	380–900 °C /%	Mass Found /amu
**1**@SrTiO_3_	0.8	18	1.0	18; 44
**1**@SrTiO_3_-a	0.6	18	1.2	18; 44; 81
**1**@SrTiO_3_-OH	1.6	18	1.3	18; 44
**1**@BaTiO_3_	1.4	18	1.0	18; 44
**1**@BaTiO_3_-a	1.5	18	1.2	18; 44; 81
**1**@BaTiO_3_-OH	3.3	18	1.5	18; 44

(**1**@) represents ligand **1** bound to the NP surface, (-a) represents using HNO_3_-activated NPs during the functionalization, and (-OH) represents using H_2_O_2_-activated NPs during the functionalization.

**Table 3:** TGA-MS results of **rR**@SrTiO_3_ and **rR**@BaTiO_3_ NPs using non-activated and activated NPs.

Entry	30–380 °C /%	Mass Found /amu	380–900 °C /%	Mass Found /amu
**rR**@SrTiO_3_	0.9	18; 44	1.5	18; 44
**rR**@SrTiO_3_-a	0.7	18; 44	1.4	18; 44
**rR**@SrTiO_3_-OH	1.5	18; 44	1.6	18; 44
**rR**@SrTiO_3_-OH-A	1.2	18	5.3	18; 44; 48; 64
**rR**@BaTiO_3_	0.9	18; 44	1.2	18; 44
**rR**@BaTiO_3_-a	1.6	18; 44	1.5	18; 44
**rR**@BaTiO_3_-OH	3.2	18; 44	1.6	18; 44
**rR**@BaTiO_3_-OH-A	2.9	18	7.6	18; 44; 48; 64

(**rR**@) represents anchoring ligand **1**-f-NPs bound to the surface of the NPs and complexed with RuCl_3_ and RhCl_3_ at a ratio of roughly 1:20; (-a) represents using HNO_3_ activated NPs during the functionalization; (-OH) means H_2_O_2_ activated NPs during the functionalization; (-OH-A) refers to additionally adjusting the pH to 1.5 during the complexation.

**Table 4:** Dihydrogen-generating experiments.

Entry	GCI ^a^ /a.u.	H_2_ /mL	Rate /mL h^−1^
**rR**@SrTiO_3_	150,100	3.10	0.77
**rR**@SrTiO_3_-a	216,900	4.47	1.12
**rR**@SrTiO_3_-OH	31,800	0.66	0.16
**rR**@SrTiO_3_-OH-A	94,900	1.96	0.49
**rR**@BaTiO_3_	43,800	0.90	0.23
**rR**@BaTiO_3_-a	90,000	1.86	0.46
**rR**@BaTiO_3_-OH	0	0	0
**rR**@BaTiO_3_-OH-A	108,600	2.24	0.56

^a^ GC integral (GCI) was adjusted for pre-existing nitrogen headspace in the reaction vial and partial sampling during the GC measurement.

## Data Availability

Data are available from the authors upon request.

## Supplementary Materials

The following supporting information can be downloaded at: https://www.mdpi.com/article/10.3390/nano13142094/s1. Details of instrumentation and procedure, characterization details. Figures S1–S4: TGA and TGA-MS spectra for pristine NPs (SrTiO_3_, BaTiO_3_). Figures S5–S7: TGA and TGA-MS spectra for acid-activated NPs (SrTiO_3_-a, BaTiO_3_-a). Figures S8–S11: TGA and TGA-MS spectra for H_2_O_2_-activated NPs (SrTiO_3_-OH, BaTiO_3_-OH). Figures S12–S17: TGA, TGA-MS, and MALDI spectra for pristine with ligand **1**-functionalized NPs (**1**@SrTiO_3_, **1**@BaTiO_3_). Figures S18–S22: TGA, TGA-MS, and MALDI spectra for acid-activated with ligand **1**-functionalized NPs (**1**@SrTiO_3_-a, **1**@BaTiO_3_-a). Figures S23–S28: TGA, TGA-MS, and MALDI spectra for H_2_O_2_-activated with ligand **1**-functionalized NPs (**1**@SrTiO_3_-OH, **1**@BaTiO_3_-OH). Figures S29–S35: TGA, TGA-MS, and MALDI spectra for pristine with ligand **1**-functionalized NPs and ruthenium- and bpy-complexed NPs (**Ru**@SrTiO_3_, **Ru**@BaTiO_3_). Figures S36–S40: TGA, TGA-MS, and MALDI spectra for pristine with ligand **1**-functionalized NPs and ruthenium-, rhodium-, and bpy-complexed NPs (**rR**@SrTiO_3_, **rR**@BaTiO_3_). Figure S41–S45: TGA, TGA-MS, and MALDI spectra for acid-activated with ligand **1**-functionalized NPs and ruthenium-, rhodium-, and bpy-complexed NPs (**rR**@SrTiO_3_-a, **rR**@BaTiO_3_-a). Figures S46–S51: TGA, TGA-MS, and MALDI spectra for H_2_O_2_-activated with ligand **1**-functionalized NPs and ruthenium-, rhodium-, and bpy-complexed NPs (**rR**@SrTiO_3_-OH, **rR**@BaTiO_3_-OH). Figures S52–S55: TGA and MALDI spectra for H_2_O_2_-activated with ligand **1**-functionalized NPs and under adjusted pH with ruthenium-, rhodium-, and bpy-complexed NPs (**rR**@SrTiO_3_-OH-A, **rR**@BaTiO_3_-OH-A) Figure S56. FTIR spectra of commercial SrTiO_3_ NPs mixed with aqueous H_2_SO_4_.
