# Supplementary material for: Nanoparticulate Perovskites for Photocatalytic Water Reduction
Source: Nanomaterials (Basel). 2023 Jul 18;13(14):2094. doi: 10.3390/nano13142094 (PMC10386032; doi:10.3390/nano13142094)
Supplement: Supplementary file 1 [file nanomaterials-13-02094-s001.zip › nanomaterials-2502256-supplementary.pdf]

nanomaterials

MDPI

Supplementary Materials

Nanoparticulate Perovskites for Photocatalytic Water Reduction

Sven A. Freimann, Catherine E. Housecroft and Edwin C. Constable *

Department of Chemistry, University of Basel, Mattenstrasse 22, BPR 1095, Postfach, 4002 Basel, Switzerland; s.freimann@unibas.ch (S.A.F.); catherine.housecroft@unibas.ch (C.E.H.)

* Correspondence: edwin.constable@unibas.ch

1. Experimental Details

1.1. Instruments

^{1}H NMR spectra were measured at 298 K on a Bruker Avance III-500 NMR spectrometer (Bruker BioSpin AG, Fällanden, Switzerland). ^{1}H chemical shifts were referenced to residual solvent peaks with respect to δ(TMS) = 0 ppm for ^{1}H. Reactions and procedures under microwave conditions were carried out in a Biotage Initiator 8 reactor. Microwave vials (5 mL and 20 mL) were from Biotage (Biotage EU, 75103 Uppsala, Sweden) and were selected depending on the required solvent volume. For centrifugation an Eppendorf Centrifuge 5415 R (Vaudaux-Eppendorf AG, 4124 Schönenbuch, Switzerland) was used with 2-, 10- and 50 mL samples. Irradiation experiments were done in a Biotage 5 mL microwave vial with a LOT Quantum Design LS0811 instrument (LOT-QuantumDesign GmbH, Darmstadt, Germany) as light source at 1200 W m^{-2}. The simulated light power was calibrated with a silicon reference cell. Solid-state absorption spectra were recorded on an Agilent Cary 5000 spectrophotometer (Agilent Technologies Inc., Santa Clara, CA, US) with a Diffuse Reflectance Accessory. For each solid-state absorption spectrum, a baseline correction was done with activated NPs as the reference sample. FTIR spectra were recorded on a Perkin Elmer UATR Two spectrophotometer (Perkin Elmer, 8603 Schwerzenbach, Switzerland). MALDI mass spectra were measured using a Shimadzu MALDI-8020 (Shimadzu Schweiz GmbH, 4153 Reinach, Switzerland) with α-cyano-4-hydroxycinnamic acid (CHCA) solution as matrix for sample preparation. Thermogravimetric analysis (TGA) was performed on a TGA5500 (TA Instruments, New Castle, DE 19720, USA) instrument under nitrogen and coupled to a MKII mass spectrometer. Initially, the temperature was held at 30 °C for 10 min before heating at a rate of 10 °C/min to 120 °C. This temperature was maintained for 30 min to remove all traces of water. Afterwards the sample was heated to 900 °C at a rate of 10 °C/min. After maintaining the temperature at 900 °C for 30 min, the sample was cooled to ambient temperature. Autoclave reactions were done in a 23 mL Acid digestion vessel (Parr Instrument Company, Moline, IL 61265, USA) using a forced convection chamber furnace (Nabertherm NA15/65, Fisher Scientific GmbH, 58239 Schwerte, Germany).

Gas chromatography was performed on a Model 8610C Gas Chromatograph from SRI instruments (SRI Instruments Europe GmbH, 53604 Bad Honnef, Germany) equipped with a flame ionization detector (FID/meth 300C hi gain), thermal conductivity detector (TCD100C low current), Haysep D 3 m 1/8'' AD 2 mm ID Mesh 80/100 column and a HT2000H headspace autosampler from HTA S.R.L. The method used a constant column temperature of 90°C, 3 minutes event time, N_2 carrier gas at 10 mL min^{-1} and 1 mL isocratic sampling. The GC integral was measured, adjusted for pre-existing nitrogen volume, sampling amount and then converted to volume in mL using a H_2 GC measured blank curve.

1.2. Characterisation report

1.2.1. HNO_3 activation of pristine $SrTiO_3$ and $BaTiO_3$

The activated NPs ($SrTiO_3$-a, 1.67 g and $BaTiO_3$-a, 0.60 g) were stored in a sealed vial under N_2 after drying the NPs under high vacuum. The NPs were characterized using

TGA-MS and FTIR spectroscopy. $SrTiO_3$-a: TGA: weight loss / %, 0.6 (<380 °C), 1.0 (380 – 900 °C). TGA-MS: amu, 18 (<380 °C), 18, 44, 81 (380 – 900 °C). FTIR spectroscopy: 3384, 1635, 1414, and 534 cm^{-1}. $BaTiO_3$-a: TGA: weight loss / %, 1.5 (<380 °C), 0.5 (380 – 900 °C). TGA-MS: amu, 18 (<380 °C), 18, 44 (380 – 900 °C). FTIR spectroscopy: 3367, 1638, 1337, 1207, 1151 and 497 cm^{-1}.

1.2.2. H_2O_2 activation of pristine $SrTiO_3$ and $BaTiO_3$

The activated NPs ($SrTiO_3$-OH, 1.05 g and $BaTiO_3$-OH, 1.05 g) were stored in a sealed vial under N_2 after drying the NPs under high vacuum for 72 h. The NPs were characterized using TGA-MS and FTIR spectroscopy. $SrTiO_3$-OH: TGA: weight loss / %, 1.6 (<380 °C), 1.3 (380 – 900 °C). TGA-MS: amu, 18, 44 (<380 °C), 18, 44 (380 – 900 °C). FTIR spectroscopy: 3396, 1638, 1446, 1404, 1037 and 543 cm^{-1}. $BaTiO_3$-OH: TGA: weight loss / %, 3.6 (<380 °C), 0.8 (380 – 900 °C). TGA-MS: amu, 18, 44 (<380 °C), 18, 44 (380 – 900 °C). FTIR spectroscopy: 3348, 1635, 1435, 1392, 974, 924 and 506 cm^{-1}.

1.2.3. Surface-functionalization of pristine and activated NPs using anchoring ligand 1

The functionalized white f-NPs **1**@$SrTiO_3$ NPs (218.1 mg, 221.6 mg), **1**@$SrTiO_3$-a NPs (418.3 mg), **1**@$SrTiO_3$-OH NPs (422.6 mg), **1**@$BaTiO_3$ NPs (210.7 mg, 219.4 mg), **1**@$BaTiO_3$-a NPs (409.9 mg), **1**@$BaTiO_3$-OH NPs (425.1 mg) were stored in a sealed vial under N_2 after drying the NPs under high vacuum for 24 – 72 h. The NPs were characterized using NMR spectroscopy, TGA-MS, FTIR spectroscopy and solid-state absorption spectroscopy. **1**@$SrTiO_3$: TGA: weight loss / %, 0.8 (<380 °C), 1.0 (380 – 900 °C). TGA-MS: amu, 18 (<380 °C), 18, 44 (380 – 900 °C). FTIR spectroscopy: 3376, 3171, 1637, 1583, 1535, 1459, 1411, 1349, 1154, 1108, 1094, 1006 and 540 cm^{-1}. Solid-state absorption spectroscopy: no absorption bands within 350–700 nm. MALDI m/z: 317.0 $[(1) + H]^+$ (calc. 317.0). **1**@$SrTiO_3$-a: TGA: weight loss / %, 0.6 (<380 °C), 1.2 (380 – 900 °C). TGA-MS: amu, 18 (<380 °C), 18, 44, 81 (380 – 900 °C). FTIR spectroscopy: 3368, 1623, 1589, 1407, 1161, 1028 and 541 cm^{-1}. Solid-state absorption spectroscopy: 390 – 450 (broad) and 590 – 630 nm (broad). MALDI m/z: 317.0 $[(1) + H]^+$ (calc. 317.0). **1**@$SrTiO_3$-OH: TGA: weight loss / %, 1.6 (<380 °C), 1.3 (380 – 900 °C). TGA-MS: amu, 18 (<380 °C), 18, 44 (380 – 900 °C). FTIR spectroscopy: 3366, 1635, 1585, 1530, 1463, 1411, 1350, 1151, 1106, 1093, 1033, 1006 and 541 cm^{-1}. Solid-state absorption spectroscopy: 390 – 450 (broad), 530 – 570 (broad), 590 – 620 (broad) and 660 – 680 nm (broad). MALDI m/z: 317.1 $[(1) + H]^+$ (calc. 317.0). **1**@$BaTiO_3$: TGA: weight loss / %, 1.4 (<380 °C), 1.0 (380 – 900 °C). TGA-MS: amu, 18 (<380 °C), 18, 44 (380 – 900 °C). FTIR spectroscopy: 3488, 3102, 1647, 1589, 1538, 1459, 1347, 1151, 1103, 1085, 1080, 988, 1000, 988 and 500 cm^{-1}. Solid-state absorption spectroscopy: 380 – 440 (broad), 530 – 570 (broad) and 600 – 620 nm (broad). MALDI m/z: 316.9 $[(1) + H]^+$ (calc. 317.0). **1**@$BaTiO_3$-a: TGA: weight loss / %, 1.5 (<380 °C), 1.2 (380 – 900 °C). TGA-MS: amu, 18 (<380 °C), 18, 44, 81 (380 – 900 °C). FTIR spectroscopy: 3359, 1639, 1583, 1531, 1462, 1374, 1351, 1149, 1103, 1101, 1074, 989, 984 and 508 cm^{-1}. Solid-state absorption spectroscopy: 380 – 420 (broad), 540 – 580 (broad) and 600 – 620 nm (broad). MALDI m/z: 316.9 $[(1) + H]^+$ (calc. 317.0). **1**@$BaTiO_3$-OH: TGA: weight loss / %, 3.3 (<380 °C), 1.2 (380 – 900 °C). TGA-MS: amu, 18 (<380 °C), 18, 44 (380 – 900 °C). FTIR spectroscopy: 3361, 1636, 1585, 1538, 1459, 1348, 1149, 1101, 1103, 1078, 983, 985, 931 and 500 cm^{-1}. Solid-state absorption spectroscopy: 390 – 430 (broad), 540 – 580 (broad), 590 – 620 (broad) and 660 – 670 nm (broad). MALDI m/z: 317.0 $[(1) + H]^+$ (calc. 317.0).

1.2.4. Surface-complexation of anchoring ligand 1 functionalized NPs using $RuCl_3$ and bpy

The complexed coloured NPs **Ru**@$SrTiO_3$ (65.8 mg, orange) and **Ru**@$BaTiO_3$ (67.0 mg, dark brown) were stored in a sealed vial under N_2 after drying the NPs under high vacuum for 24 – 72 h. The NPs were characterized using NMR spectroscopy, TGA-MS, FTIR spectroscopy and solid-state absorption spectroscopy. **Ru**@$SrTiO_3$: TGA: weight loss / %, 1.0 (<380 °C), 1.8 (380 – 900 °C). TGA-MS: amu, 18, 44 (<380 °C), 18, 44 (380 – 900 °C). FTIR spectroscopy: 3386, 3203, 2651, 1639, 1534, 1462, 1400, 1348, 1152, 1107, 1093, 1005 and 541

cm^{-1}. Solid-state absorption spectroscopy: 430 – 470, 540 – 560 (broad), 600 – 620 (broad) and 660 – 670 nm. MALDI m/z: 317.0 [(**1**) + H]$^+$ (calc. 317.0), 416.0 [Ru(bpy)$_2$ + 2 H]$^+$ (calc. 416.1), 572.0 [Ru(**1**)(bpy) − 2 H]$^+$ (calc. 572.0), 602.0 [Ru(bpy)$_2$ + CHCA − H]$^+$ (calc. 602.1), and 728.9 [Ru(**1**)(bpy)$_2$ − H]$^+$ (calc. 729.0). (CHCA = α-cyano-4-hydroxycinnamic acid). **Ru**@$BaTiO_3$: TGA: weight loss / %, 1.5 (<380 °C), 1.6 (380 – 900 °C). TGA-MS: amu, 18, 44 (<380 °C), 18, 44 (380 – 900 °C). FTIR spectroscopy: 3369, 1636, 1400, 1152, 1113, 1081, 981, 985 and 494 cm^{-1}. Solid-state absorption spectroscopy: 400 – 480, 540 – 560, 600 – 620 and 660 – 670 nm. MALDI m/z: 316.9 [(**1**) + H]$^+$ (calc. 317.0), 413.8 [Ru(bpy)$_2$]$^+$ (calc. 414.0), 603.1 [Ru(bpy)$_2$ + CHCA]$^+$ (calc. 603.1) and 728.6 [Ru(**1**)(bpy)$_2$ − H]$^+$ (calc. 729.0). (CHCA = α-cyano-4-hydroxycinnamic acid).

1.2.5. Surface-complexation of anchoring ligand 1 functionalized NPs using $RuCl_3$, $RhCl_3$ and bpy

The reaction yielded **rR**@$SrTiO_3$ (354.2 mg) and **rR**@$SrTiO_3$-a (358.2 mg) as pale orange powder, **rR**@$SrTiO_3$-OH (358.4 mg) and **rR**@$BaTiO_3$ (349.9 mg) as grey powder and, **rR**@$BaTiO_3$-a (356.9 mg) and **rR**@$BaTiO_3$-OH (357.4 mg) as dark brown powder which were sealed in a vial under N_2 after drying under high vacuum. **rR**@$SrTiO_3$: TGA: weight loss / %, 0.9 (<380 °C), 1.5 (380 – 900 °C). TGA-MS: amu, 18, 44 (<380 °C), 18, 44 (380 – 900 °C). FTIR spectroscopy: 3380, 3177, 1636, 1625, 1608, 1531, 1463, 1402, 1347, 1154, 1083, 1058 and 987 cm^{-1}. Solid-state absorption spectroscopy: 410 – 480, 540 – 570 (broad), 600 – 620 (broad) and 660 – 670 nm (broad). MALDI m/z: 317.0 [(**1**) + H]$^+$ (calc. 317.0), 415.0 [Rh(bpy)$_2$]$^+$ (calc. 415.0), 574.9 [Rh(**1**)(bpy)]$^+$ (calc. 575.0) and 603.1 [Rh(bpy)$_2$ + CHCA − H]$^+$ (calc. 603.1). (CHCA = α-cyano-4-hydroxycinnamic acid). **rR**@$SrTiO_3$-a: TGA: weight loss / %, 0.7 (<380 °C), 1.4 (380 – 900 °C). TGA-MS: amu, 18, 44 (<380 °C), 18, 44 (380 – 900 °C). FTIR spectroscopy: 3404, 3236, 1634, 1404, 1158, 1008, 1003 and 538 cm^{-1}. Solid-state absorption spectroscopy: 410 – 480, 540 – 570 (broad), 600 – 620 (broad) and 660 – 670 nm (broad). MALDI m/z: 415.0 [Rh(bpy)$_2$]$^+$ (calc. 415.0) and 603.0 [Rh(bpy)$_2$ + CHCA − H]$^+$ (calc. 603.1). (CHCA = α-cyano-4-hydroxycinnamic acid). **rR**@$SrTiO_3$-OH: TGA: weight loss / %, 1.5 (<380 °C), 1.6 (380 – 900 °C). TGA-MS: amu, 18, 44 (<380 °C), 18, 44 (380 – 900 °C). FTIR spectroscopy: 3382, 1636, 1583, 1534, 1457, 1402, 1350, 1152, 1108, 1092, 1005 and 543 cm^{-1}. Solid-state absorption spectroscopy: 410 – 480, 540 – 570, 600 – 620 and 660 – 680 nm. MALDI m/z: 317.1 [(**1**) + H]$^+$ (calc. 317.0), 415.0 [Rh(bpy)$_2$]$^+$ (calc. 415.0), 574.8 [Rh(**1**)(bpy)]$^+$ (calc. 575.0) and 603.1 [Rh(bpy)$_2$ + CHCA − H]$^+$ (calc. 603.1). (CHCA = α-cyano-4-hydroxycinnamic acid). **rR**@$BaTiO_3$: TGA: weight loss / %, 0.9 (<380 °C), 1.2 (380 – 900 °C). TGA-MS: amu, 18, 44 (<380 °C), 18, 44 (380 – 900 °C). FTIR spectroscopy: 3325, 1632, 1457, 1402, 1345, 1152, 1110, 1082, 983 and 493 cm^{-1}. Solid-state absorption spectroscopy: 400 – 480, 540 – 570, 600 – 620 and 660 – 670 nm. MALDI m/z: 316.9 [(**1**) + H]$^+$ (calc. 317.0), 414.8 [Rh(bpy)$_2$]$^+$ (calc. 415.0), 574.7 [Rh(**1**)(bpy) + H]$^+$ (calc. 575.0) and 604.9 [Rh(bpy)$_2$ + CHCA + H]$^+$ (calc. 605.1). (CHCA = α-cyano-4-hydroxycinnamic acid). **rR**@$BaTiO_3$-a: TGA: weight loss / %, 1.6 (<380 °C), 1.5 (380 – 900 °C). TGA-MS: amu, 18, 44 (<380 °C), 18, 44 (380 – 900 °C). FTIR spectroscopy: 3346, 1634, 1450, 1401, 1345, 1148, 1103, 1075, 984, and 493 cm^{-1}. Solid-state absorption spectroscopy: 400 – 480, 540 – 570, 600 – 620 and 660 – 670 nm. MALDI m/z: 316.9 [(**1**) + H]$^+$ (calc. 317.0), 414.9 [Rh(bpy)$_2$]$^+$ (calc. 415.0) and 602.8 [Rh(bpy)$_2$ + CHCA − H]$^+$ (calc. 603.1). (CHCA = α-cyano-4-hydroxycinnamic acid). **rR**@$BaTiO_3$-OH: TGA: weight loss / %, 3.2 (<380 °C), 1.6 (380 – 900 °C). TGA-MS: amu, 18, 44 (<380 °C), 18, 44 (380 – 900 °C). FTIR spectroscopy: 3346, 1635, 1584, 1556, 1420, 1349, 1103, 1078, 986 and 493 cm^{-1}. Solid-state absorption spectroscopy: 400 – 480, 540 – 570, 600 – 620 and 660 – 670 nm. MALDI m/z: 317.0 [(**1**) + H]$^+$ (calc. 317.0) and 414.9 [Rh(bpy)$_2$]$^+$ (calc. 415.0). (CHCA = α-cyano-4-hydroxycinnamic acid).

1.2.6. Surface-complexation of anchoring ligand 1 functionalized NPs using $RuCl_3$, $RhCl_3$ and bpy under adjusted pH

The complexed pale orange NPs **rR**@$SrTiO_3$-OH-A (356.0 mg), and **rR**@$BaTiO_3$-OH-A (355.1 mg) were stored in a sealed vial under N2 after drying under high vacuum.

rR@$SrTiO_3$-OH-A: TGA: weight loss / %, 1.1 (<380 °C), 5.3 (380 – 900 °C). TGA-MS: amu, 18 (<380 °C), 18, 44, 48, 64 (380 – 900 °C). FTIR spectroscopy: 3405, 1640, 1403, 1219, 1141, 1100, 996 and 541 cm^{-1}. Solid-state absorption spectroscopy: 410 – 480, 540 – 570 (broad), 600 – 620 (broad) and 660 – 670 nm (broad). MALDI m/z: 317.0 [(**1**) + H]$^+$ (calc. 317.0), 414.9 [Rh(bpy)$_2$]$^+$ (calc. 415.0), 574.9 [Rh(**1**)(bpy)]$^+$ (calc. 575.0) and 603.0 [Rh(bpy)$_2$ + CHCA − H]$^+$ (calc. 603.1). (CHCA = α-cyano-4-hydroxycinnamic acid). **rR**@$BaTiO_3$-OH-A: TGA: weight loss / %, 2.9 (<380 °C), 7.6 (380 – 900 °C). TGA-MS: amu, 18 (<380 °C), 18, 44, 48, 64 (380 – 900 °C). FTIR spectroscopy: 3377, 1632, 1454, 1403, 1200, 1091, 984, 606 and 503 cm^{-1}. Solid-state absorption spectroscopy: 400 – 470, 600 – 620 (broad) and 660 – 680 nm (broad). MALDI m/z: 317.1 [(**1**) + H]$^+$ (calc. 317.0), 415.0 [Rh(bpy)$_2$]$^+$ (calc. 415.0), 603.1 [Rh(bpy)$_2$ + CHCA − H]$^+$ (calc. 603.1) and 731.4 [Ru(**1**)(bpy)$_2$]$^+$ (calc. 731.0). (CHCA = α-cyano-4-hydroxycinnamic acid).

1.3. Supporting Figures

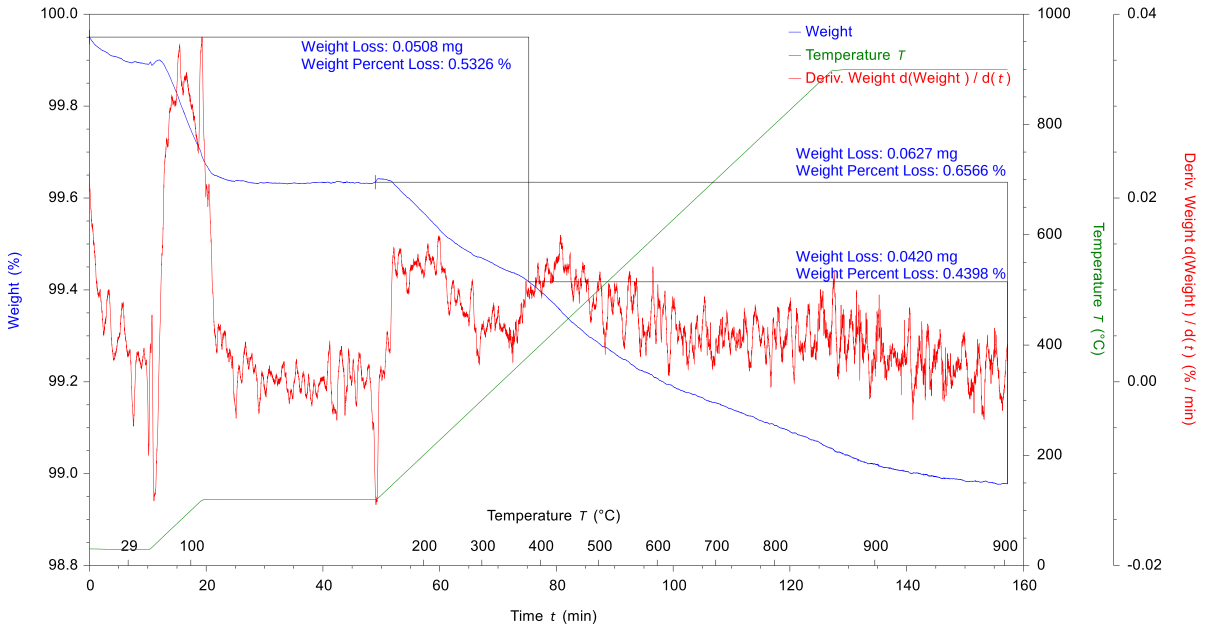

Figure S1. TGA-MS curves for pristine $SrTiO_3$ NPs; where blue is the weight loss, green is the temperature and red is the derivative weight against time.

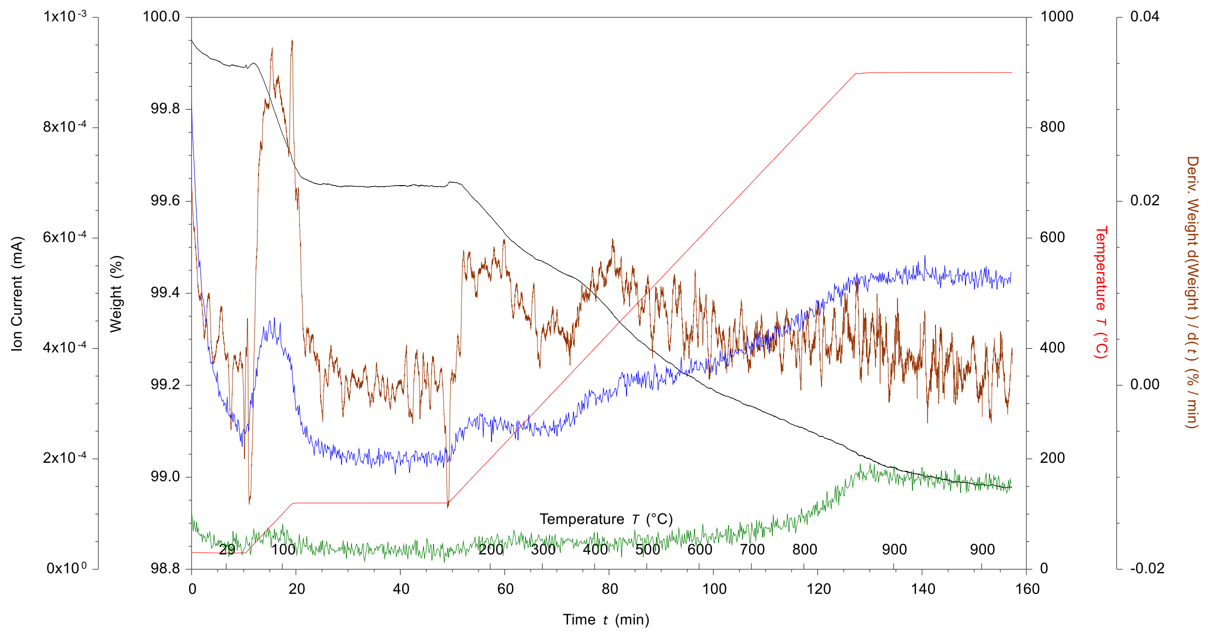

Figure S2. TGA-MS curves for pristine $SrTiO_3$ NPs; where black is the weight loss, red is the temperature, brown is the derivative weight against time, blue is the ion current of amu 18 and green is the ion current of amu 44.

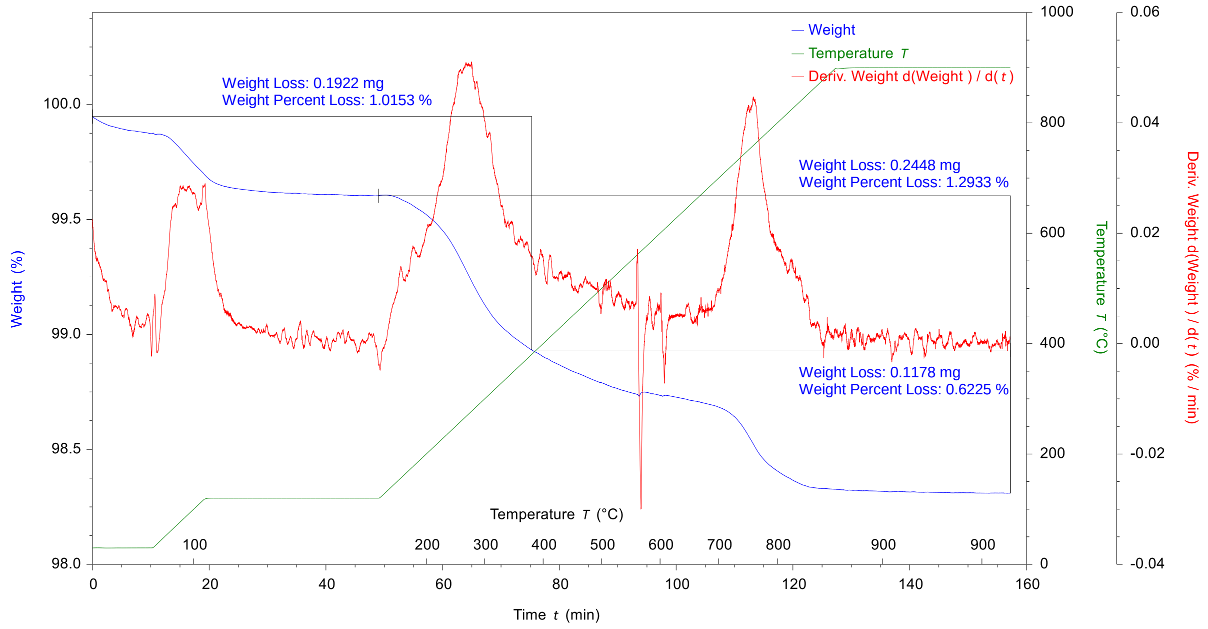

Figure S3. TGA-MS curves for pristine $BaTiO_3$ NPs; where blue is the weight loss, green is the temperature and red is the derivative weight against time.

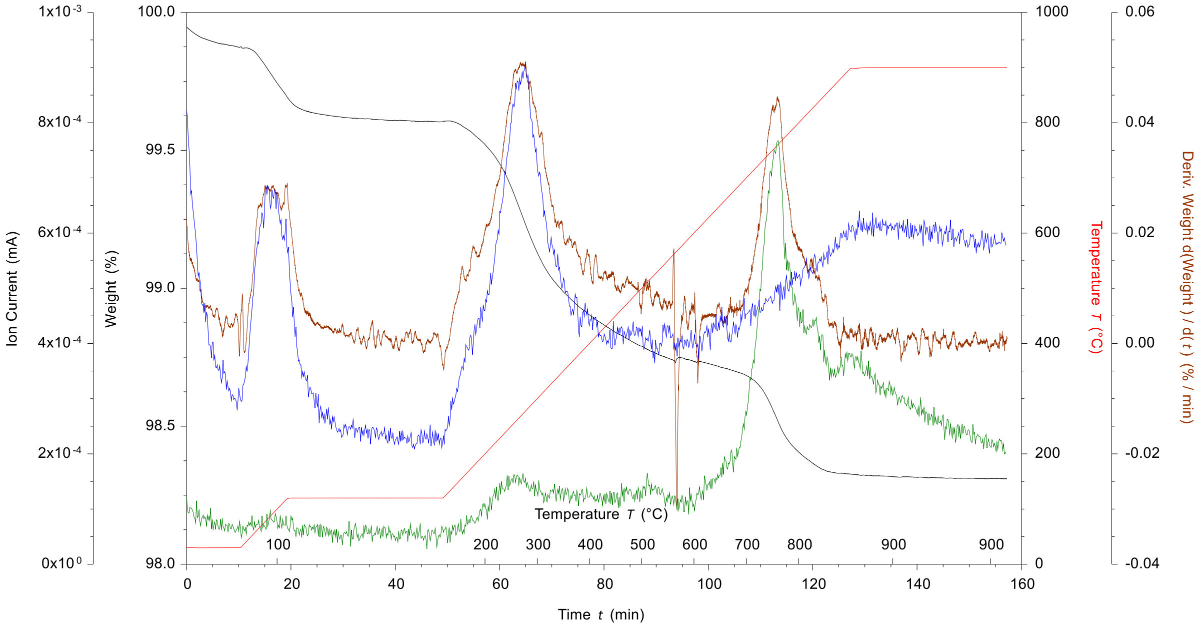

Figure S4. TGA-MS curves for pristine $BaTiO_3$ NPs; where black is the weight loss, red is the temperature, brown is the derivative weight against time, blue is the ion current of amu 18 and green is the ion current of amu 44.

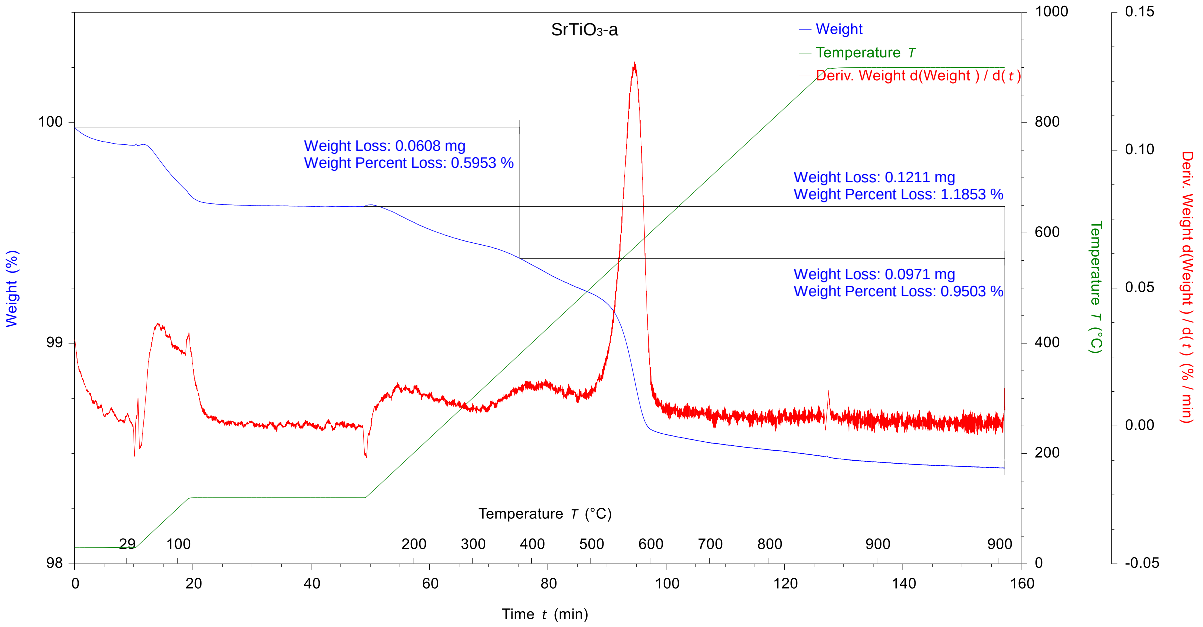

Figure S5. TGA curves for acid activated $SrTiO_3$ NPs; $SrTiO_3$-a where blue is the weight loss, green is the temperature and red is the derivative weight against time.

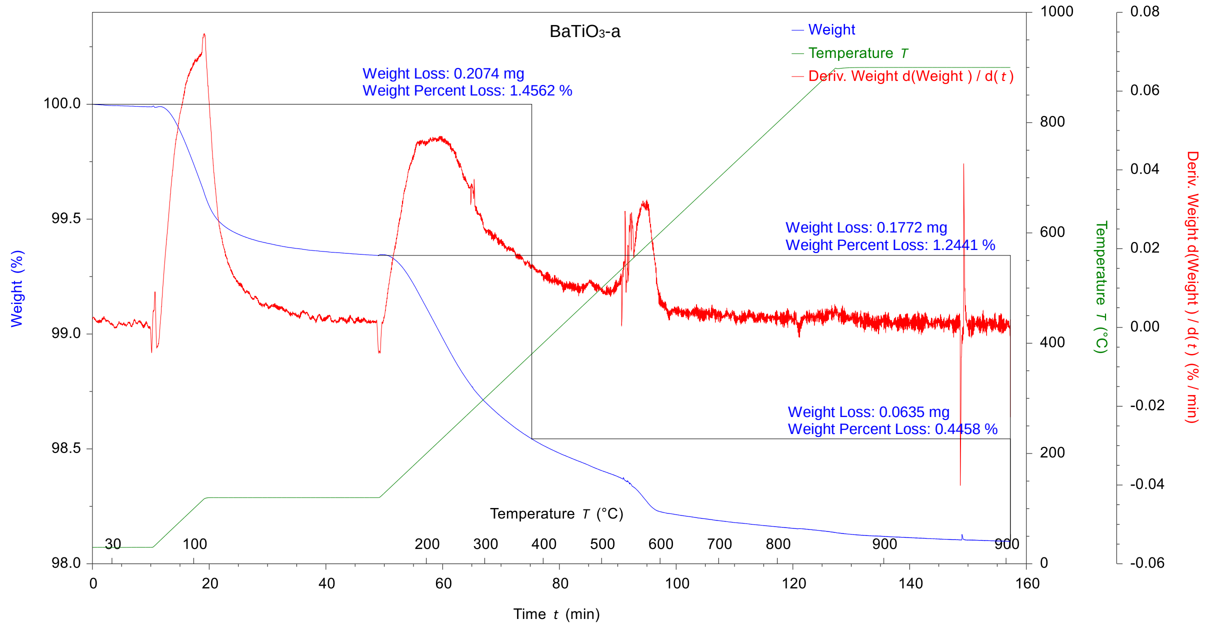

Figure S6. TGA-MS curves for acid activated $BaTiO_3$ NPs; where blue is the weight loss, green is the temperature and red is the derivative weight against time.

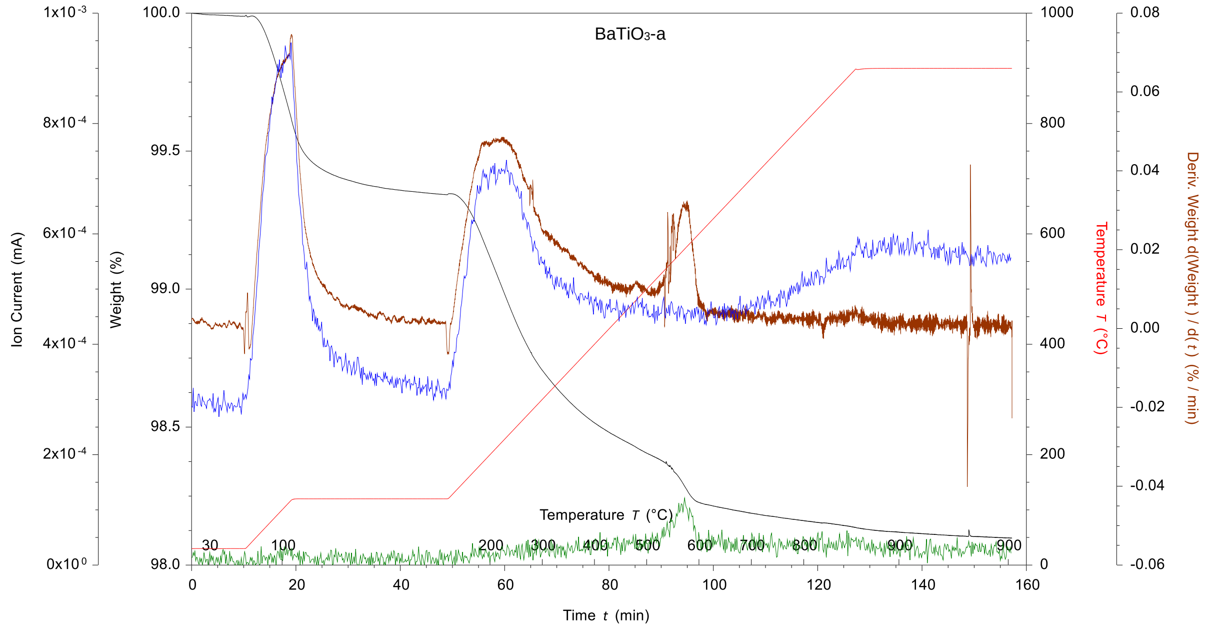

Figure S7. TGA-MS curves for acid activated $BaTiO_3$ NPs; $BaTiO_3$-a where black is the weight loss, red is the temperature, brown is the derivative weight against time, blue is the ion current of amu 18 and green is the ion current of amu 44.

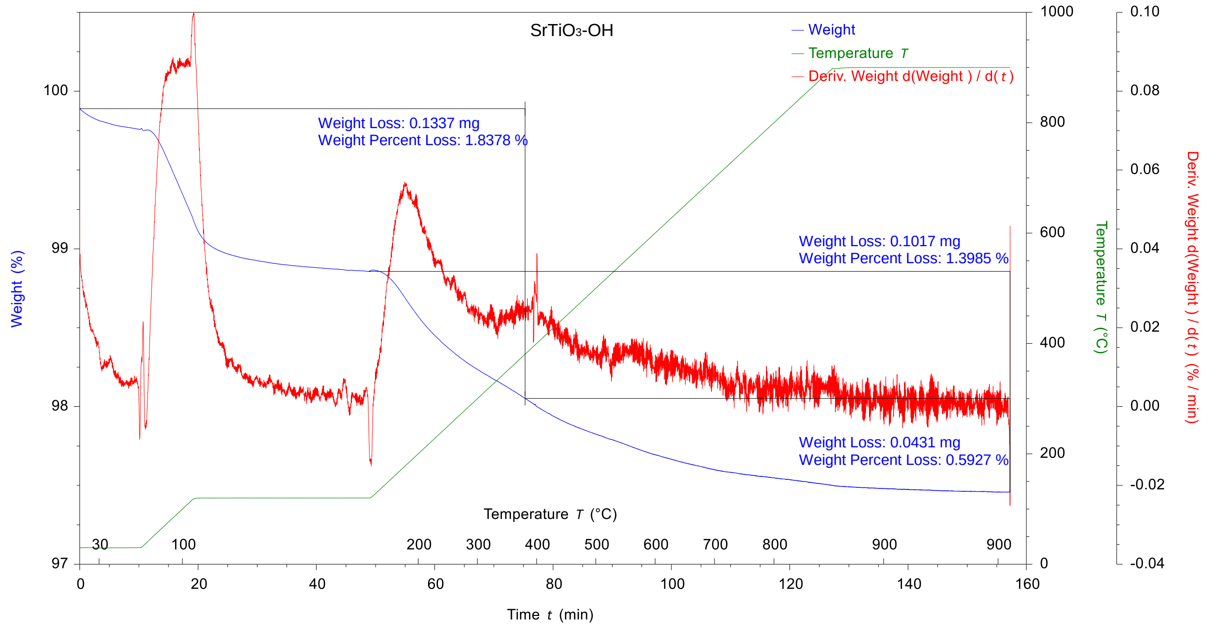

Figure S8. TGA-MS curves for H_2O_2 activated $SrTiO_3$ NPs; where blue is the weight loss, green is the temperature and red is the derivative weight against time.

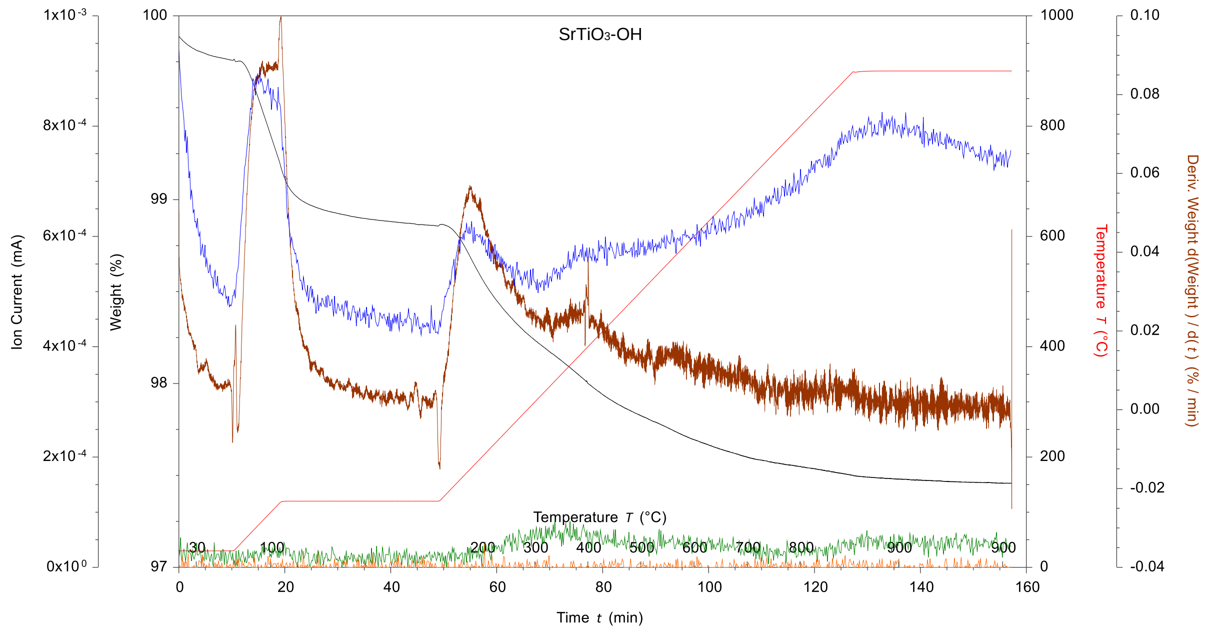

Figure S9. TGA-MS curves for H_2O_2 activated $SrTiO_3$ NPs; $SrTiO_3$-OH where black is the weight loss, red is the temperature, brown is the derivative weight against time, blue is the ion current of amu 18, green is the ion current of amu 44 and orange is the ion current of amu 81.

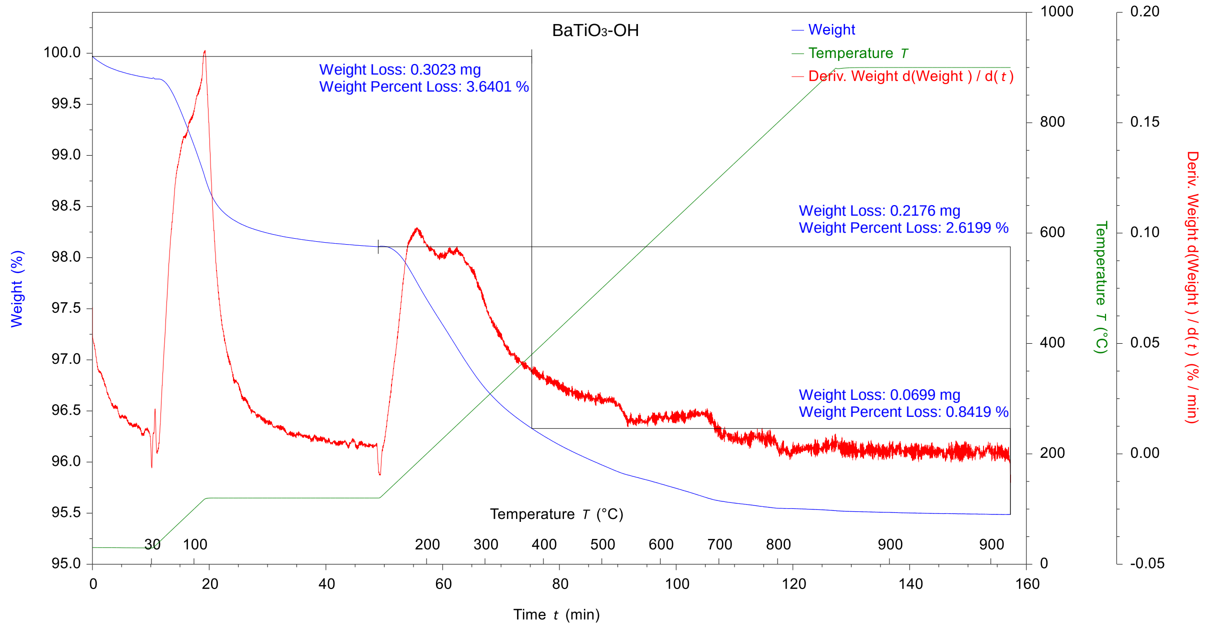

Figure S10. TGA-MS curves for H_2O_2 activated $BaTiO_3$ NPs; where blue is the weight loss, green is the temperature and red is the derivative weight against time.

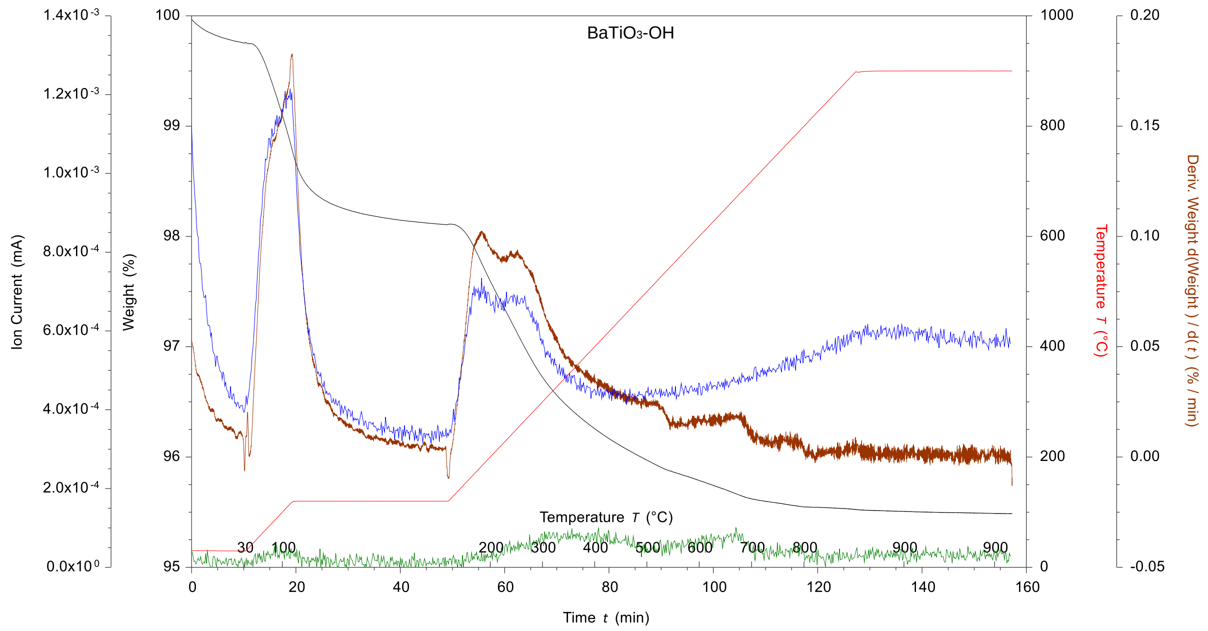

Figure S11. TGA-MS curves for H_2O_2 activated $BaTiO_3$ NPs; $BaTiO_3$-OH where black is the weight loss, red is the temperature, brown is the derivative weight against time, blue is the ion current of amu 18 and green is the ion current of amu 44.

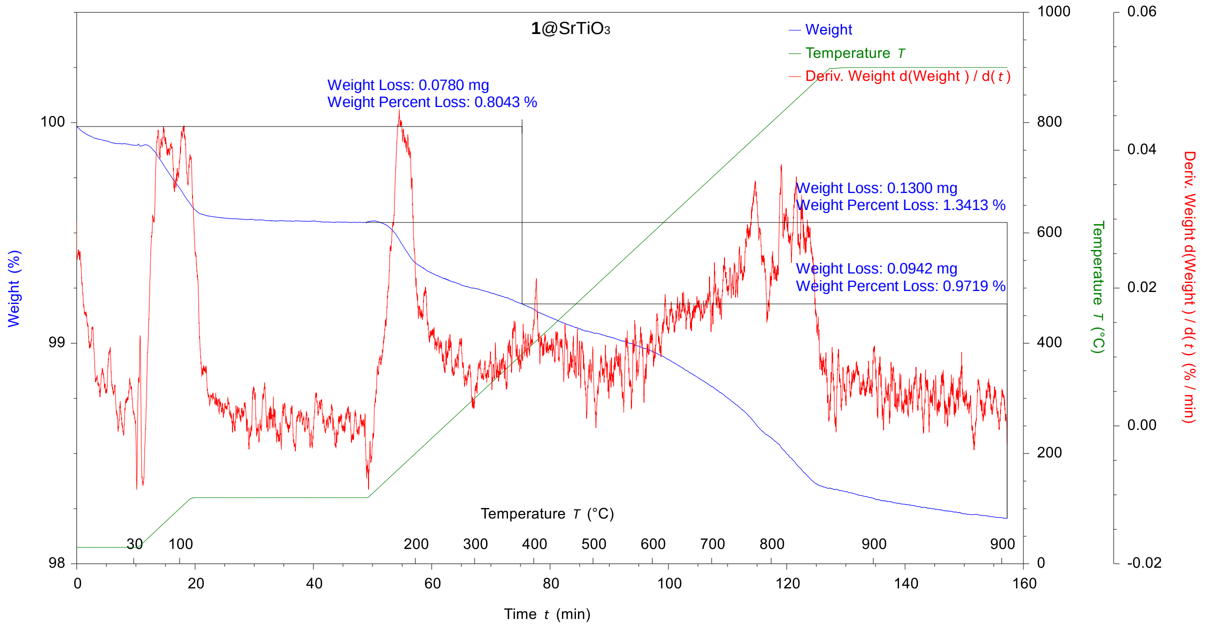

Figure S12. TGA-MS curves for with ligand **1** functionalized $SrTiO_3$ NPs; **1**@$SrTiO_3$ where blue is the weight loss, green is the temperature and red is the derivative weight against time.

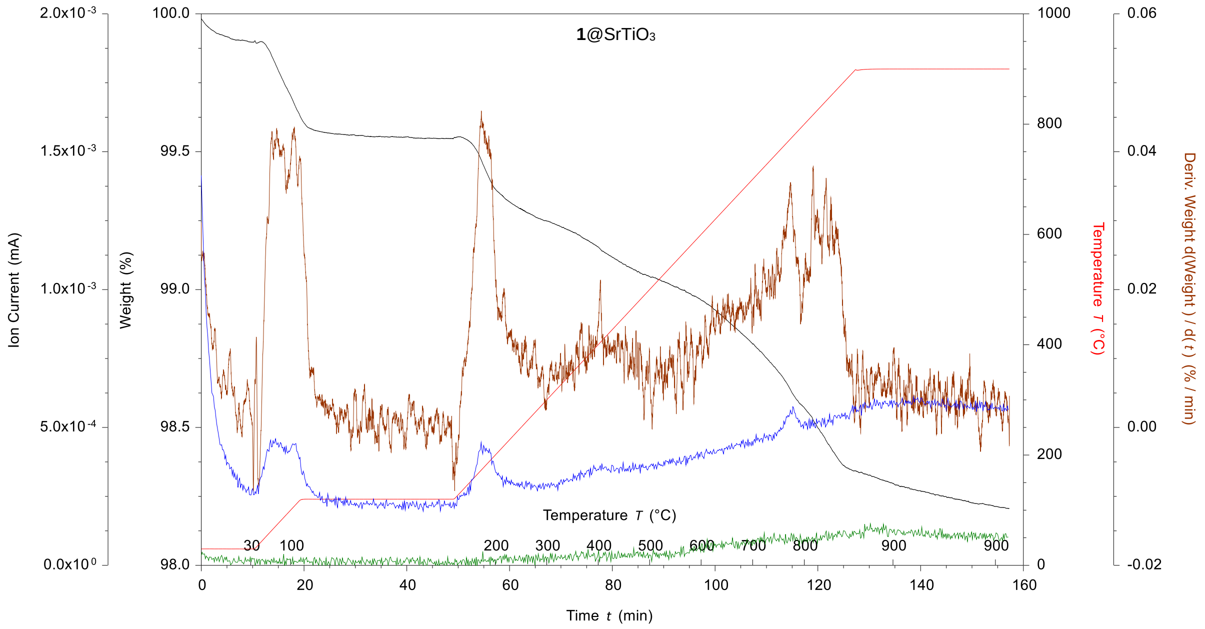

Figure S13. TGA-MS curves for with ligand **1** functionalized $SrTiO_3$ NPs; **1**@$SrTiO_3$ where black is the weight loss, red is the temperature, brown is the derivative weight against time, blue is the ion current of amu 18 and green is the ion current of amu 44.

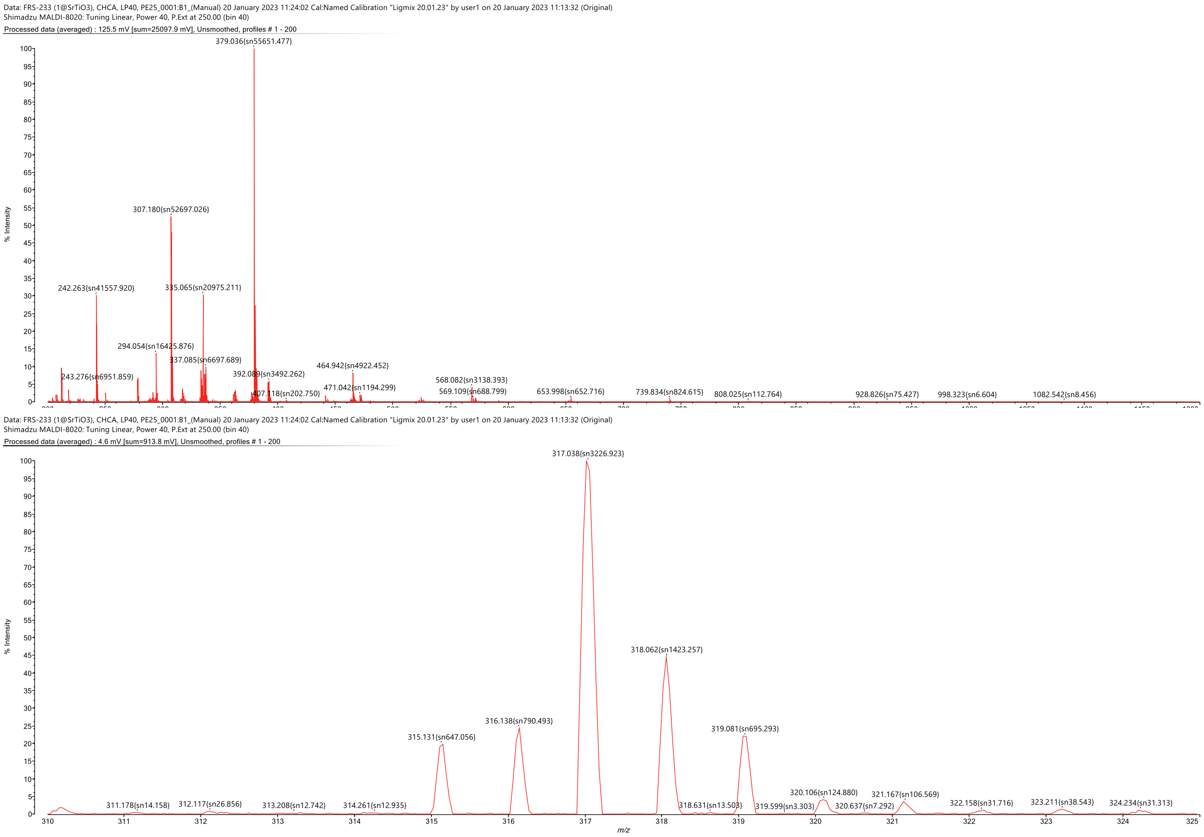

Figure S14. MALDI mass spectrum (with CHCA matrix) of **1**@$SrTiO_3$.

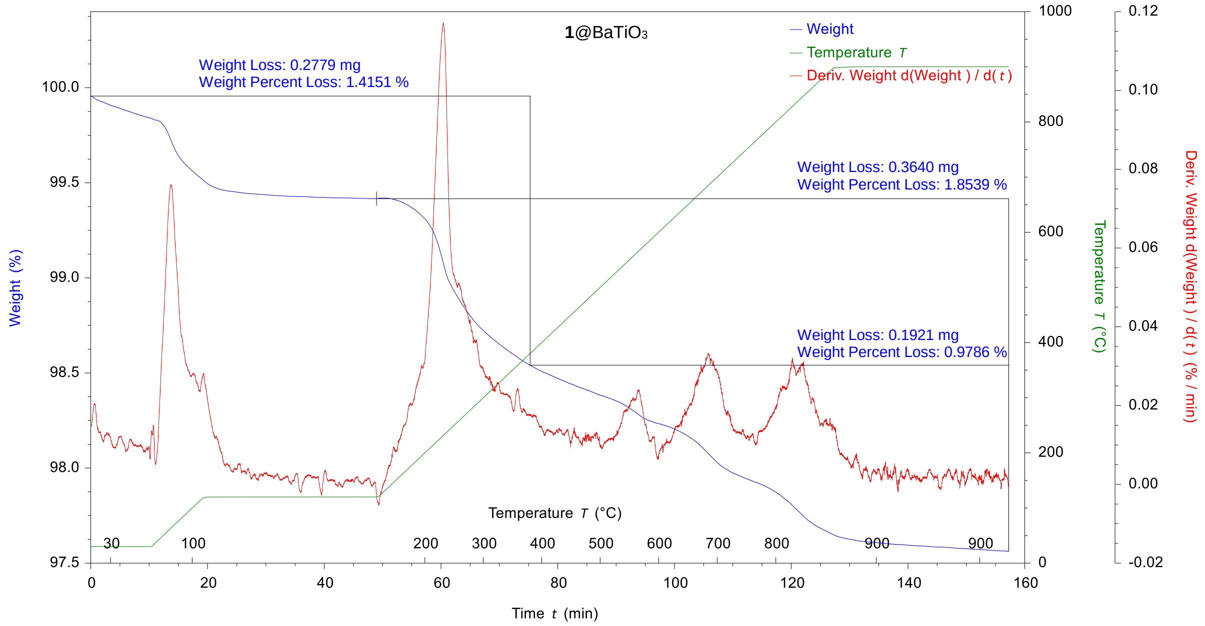

Figure S15. TGA-MS curves for with ligand **1** functionalized $BaTiO_3$ NPs; **1**@$BaTiO_3$ where blue is the weight loss, green is the temperature and red is the derivative weight against time.

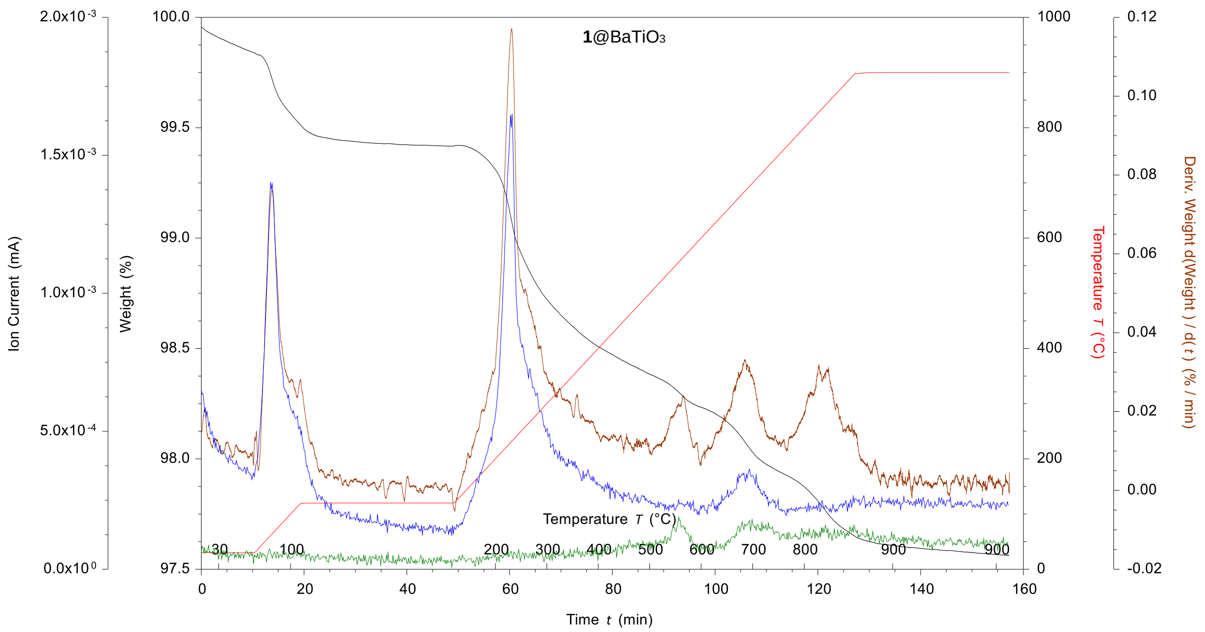

Figure S16. TGA-MS curves for with ligand **1** functionalized $BaTiO_3$ NPs; **1**@$BaTiO_3$ where black is the weight loss, red is the temperature, brown is the derivative weight against time, blue is the ion current of amu 18 and green is the ion current of amu 44.

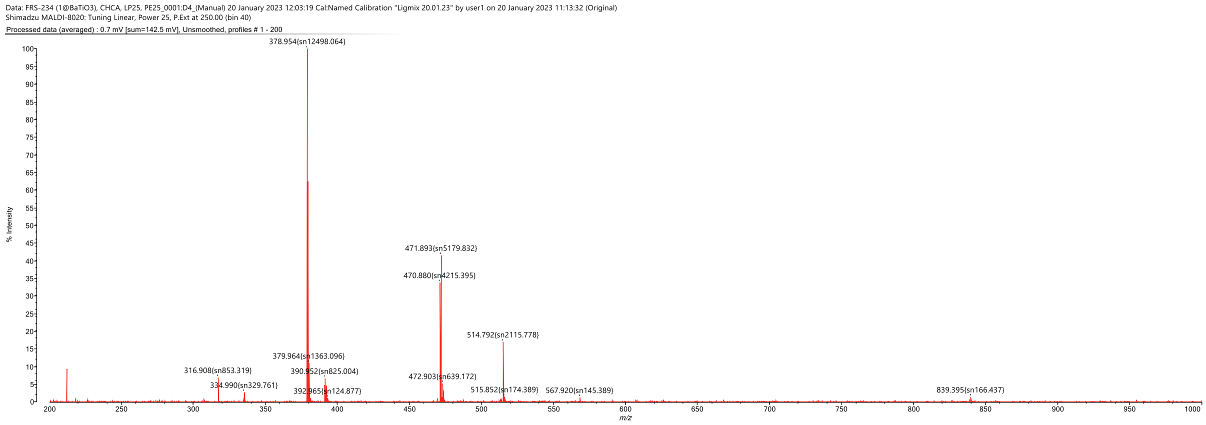

Figure S17. MALDI mass spectrum (with CHCA matrix) of **1**@$BaTiO_3$.

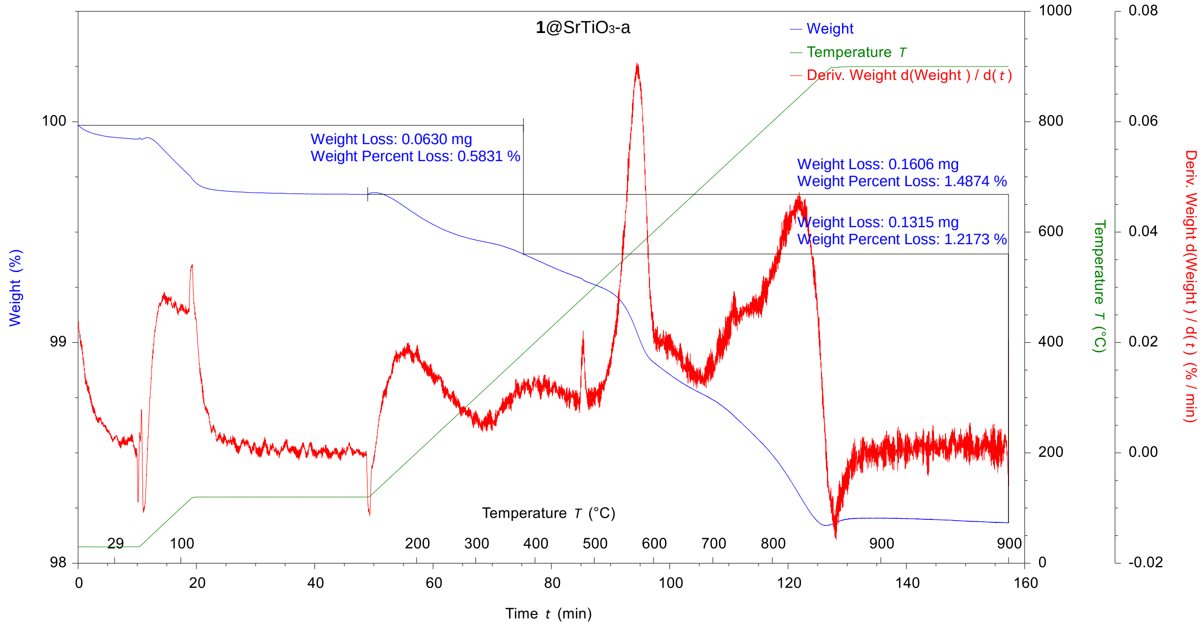

Figure S18. TGA curves for acid activated with ligand **1** functionalized $SrTiO_3$ NPs; **1**@$SrTiO_3$-a where blue is the weight loss, green is the temperature and red is the derivative weight against time.

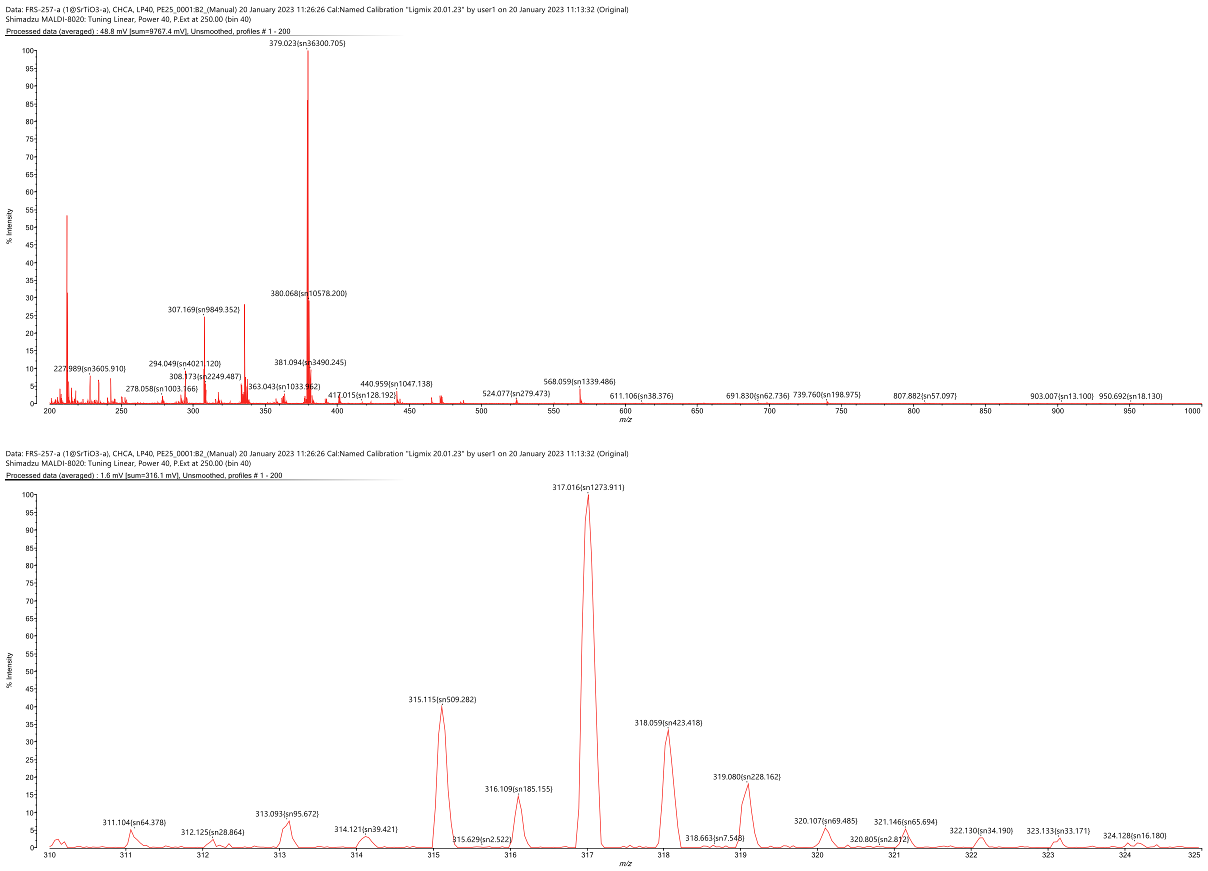

Figure S19. MALDI mass spectrum (with CHCA matrix) of **1**@$SrTiO_3$-a.

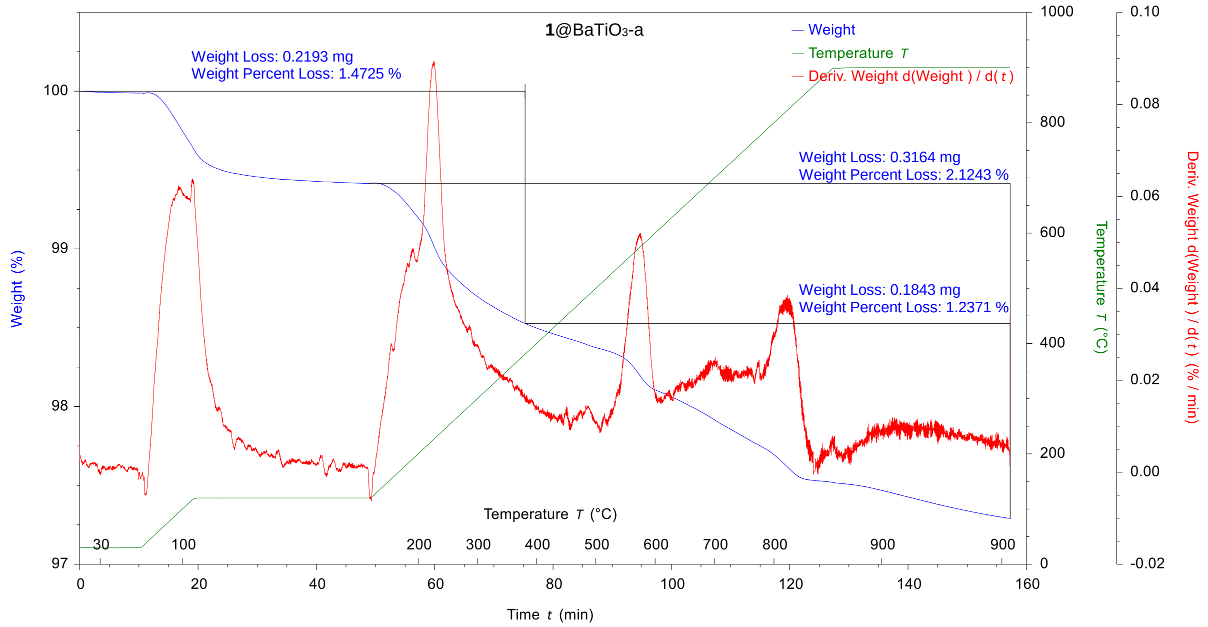

Figure S20. TGA-MS curves for acid activated with ligand **1** functionalized $BaTiO_3$ NPs; **1**@$BaTiO_3$-a where blue is the weight loss, green is the temperature and red is the derivative weight against time.

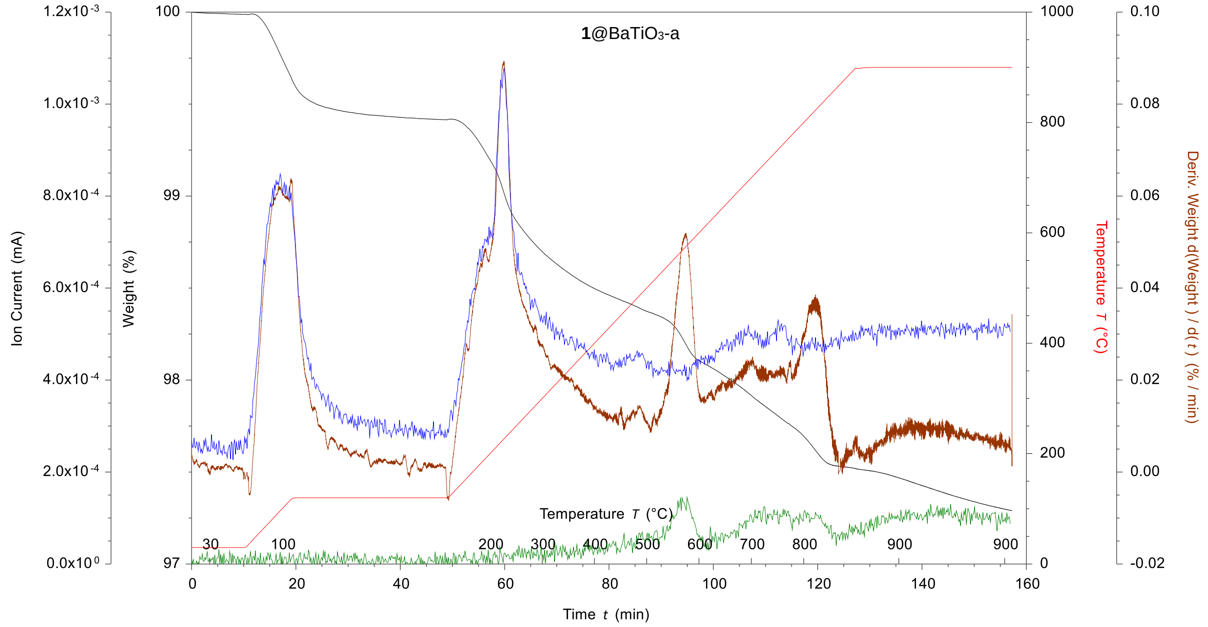

Figure S21. TGA-MS curves for acid activated with ligand **1** functionalized $BaTiO_3$ NPs; **1**@$BaTiO_3$-a where black is the weight loss, red is the temperature, brown is the derivative weight against time, blue is the ion current of amu 18 and green is the ion current of amu 44.

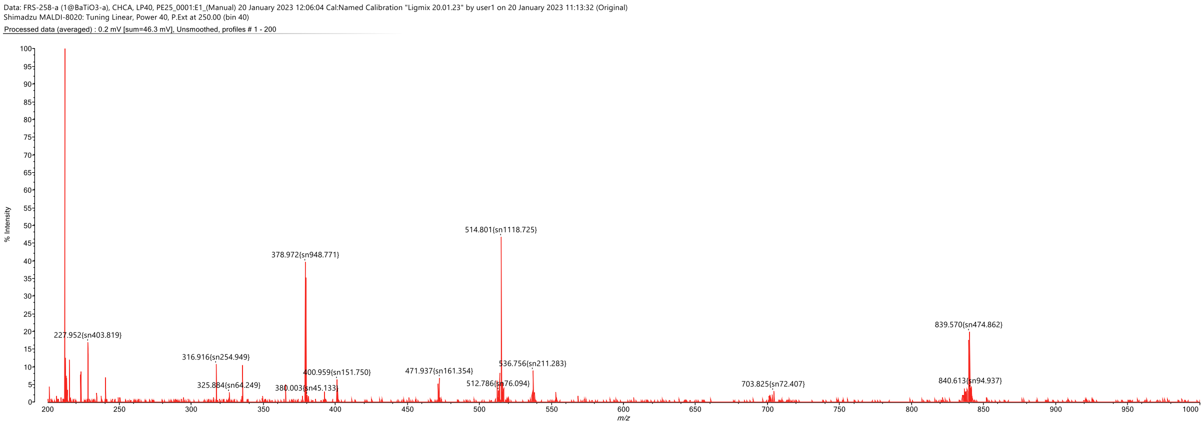

Figure S22. MALDI mass spectrum (with CHCA matrix) of **1**@$BaTiO_3$-a.

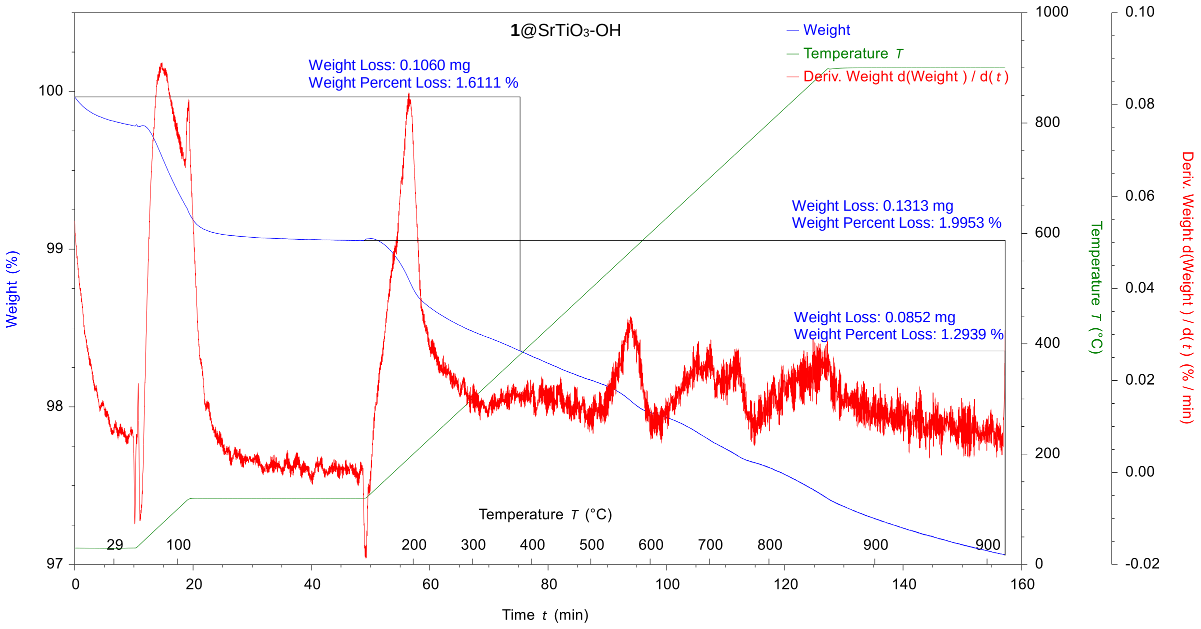

Figure S23. TGA-MS curves for H_2O_2 activated with ligand **1** functionalized $SrTiO_3$ NPs; **1**@$SrTiO_3$-OH where blue is the weight loss, green is the temperature and red is the derivative weight against time.

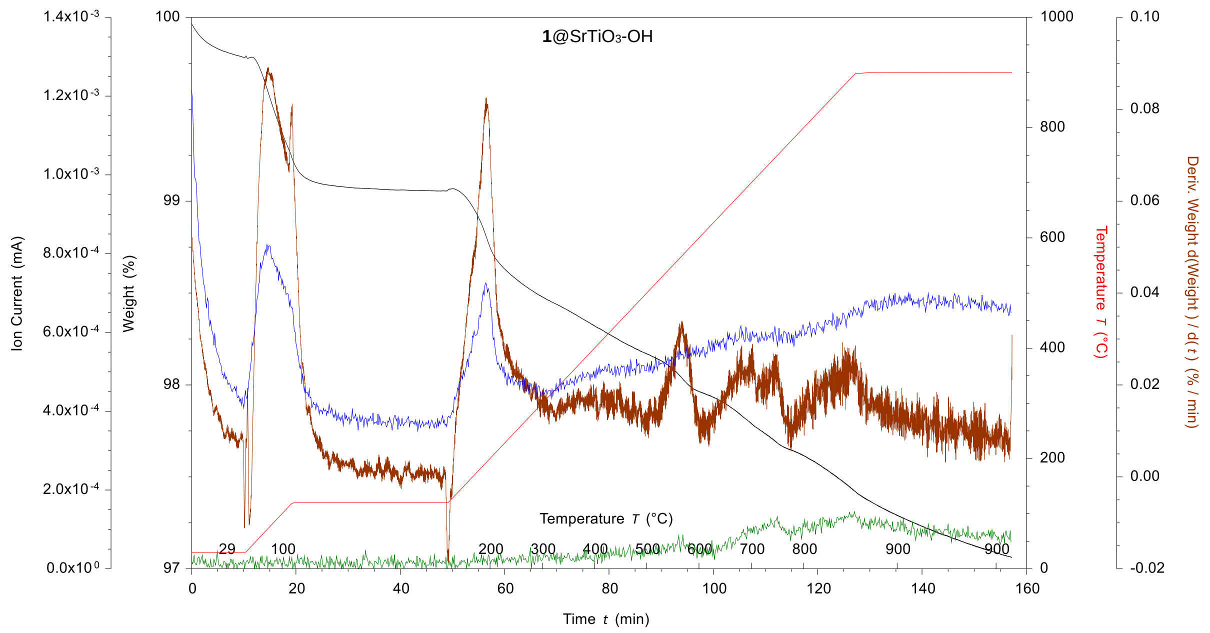

Figure S24. TGA-MS curves for H_2O_2 activated with ligand **1** functionalized $SrTiO_3$ NPs; **1**@$SrTiO_3$-OH where black is the weight loss, red is the temperature, brown is the derivative weight against time, blue is the ion current of amu 18 and green is the ion current of amu 44.

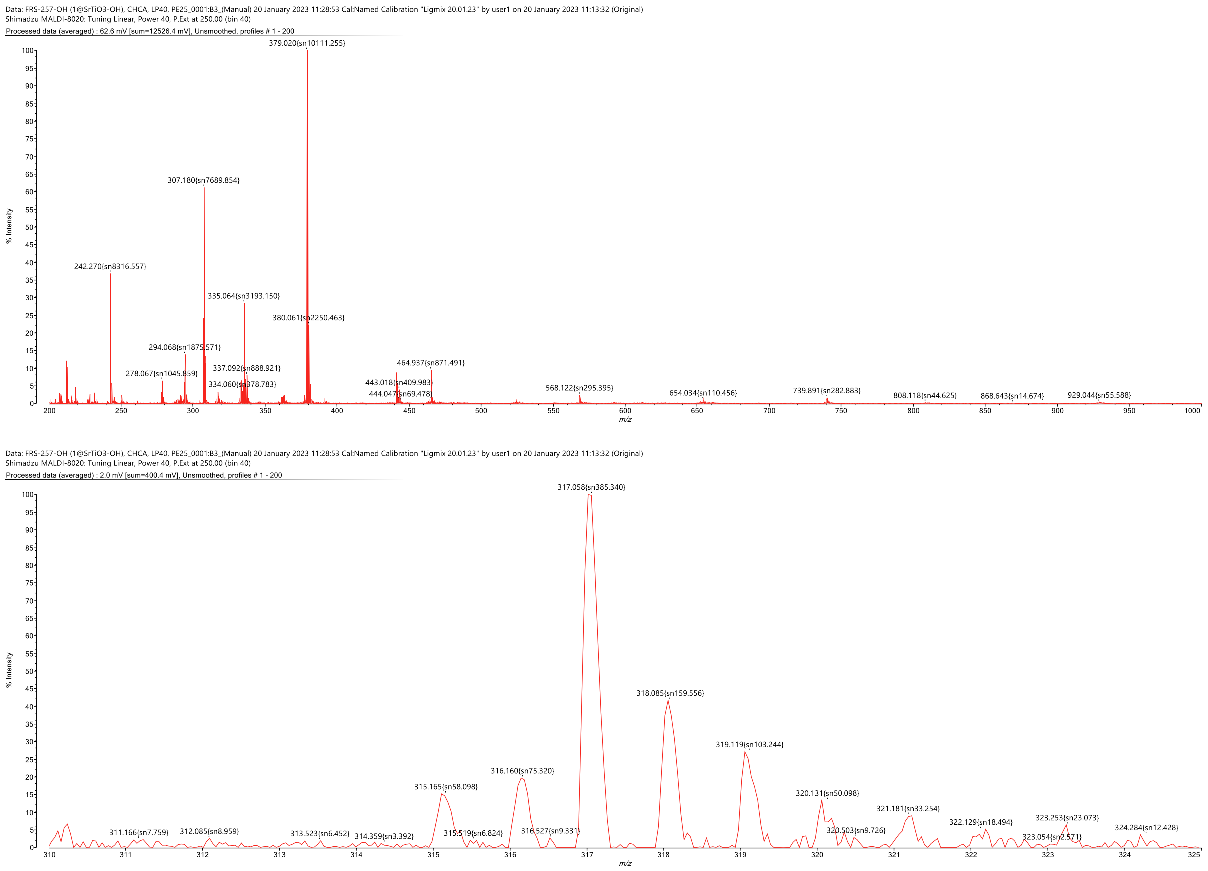

Figure S25. MALDI mass spectrum (with CHCA matrix) of **1**@$SrTiO_3$-OH.

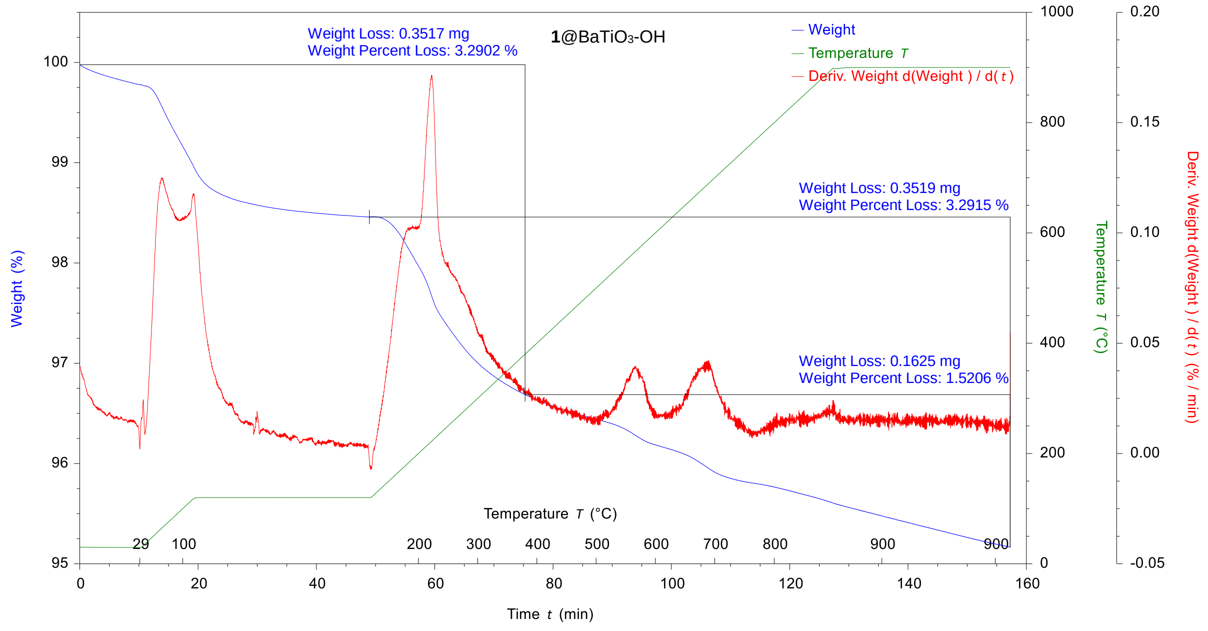

Figure S26. TGA-MS curves for H_2O_2 activated with ligand **1** functionalized $BaTiO_3$ NPs; **1**@Ba-TiO_3-OH where blue is the weight loss, green is the temperature and red is the derivative weight against time.

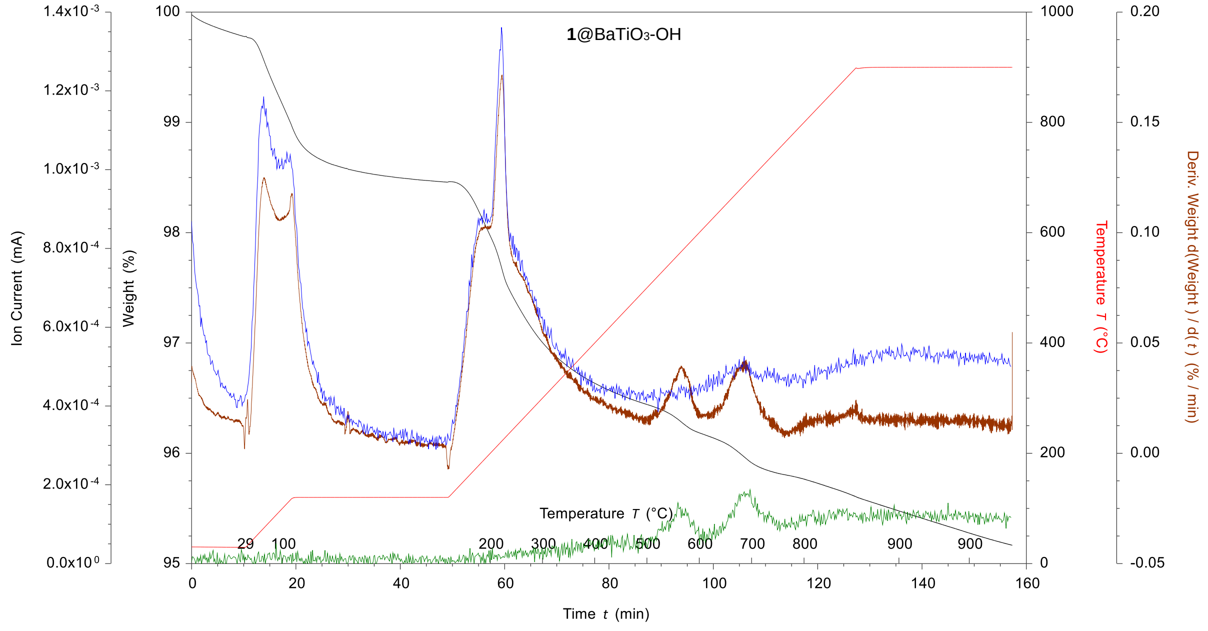

Figure S27. TGA-MS curves for H_2O_2 activated with ligand **1** functionalized $BaTiO_3$ NPs; **1**@Ba-TiO_3-OH where black is the weight loss, red is the temperature, brown is the derivative weight against time, blue is the ion current of amu 18 and green is the ion current of amu 44.

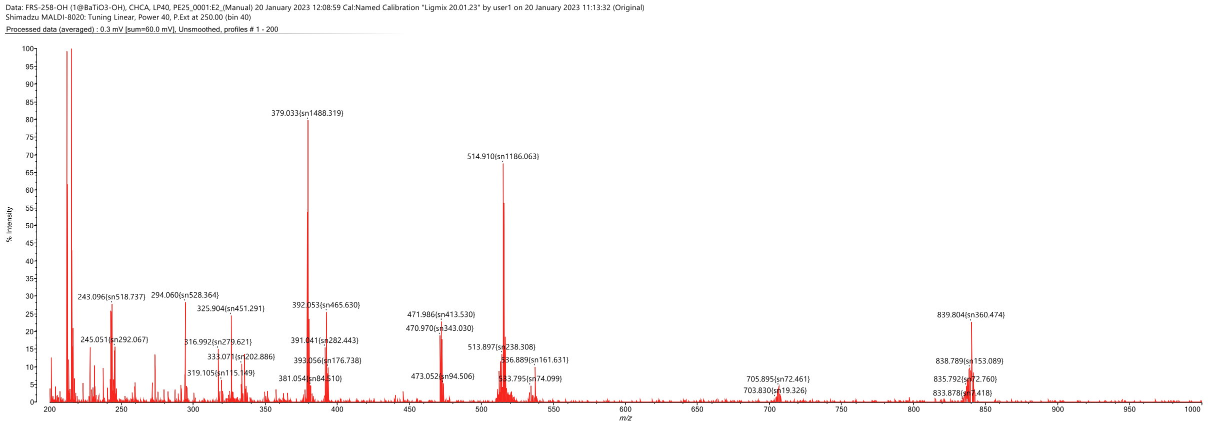

Figure S28. MALDI mass spectrum (with CHCA matrix) of **1**@$BaTiO_3$-OH.

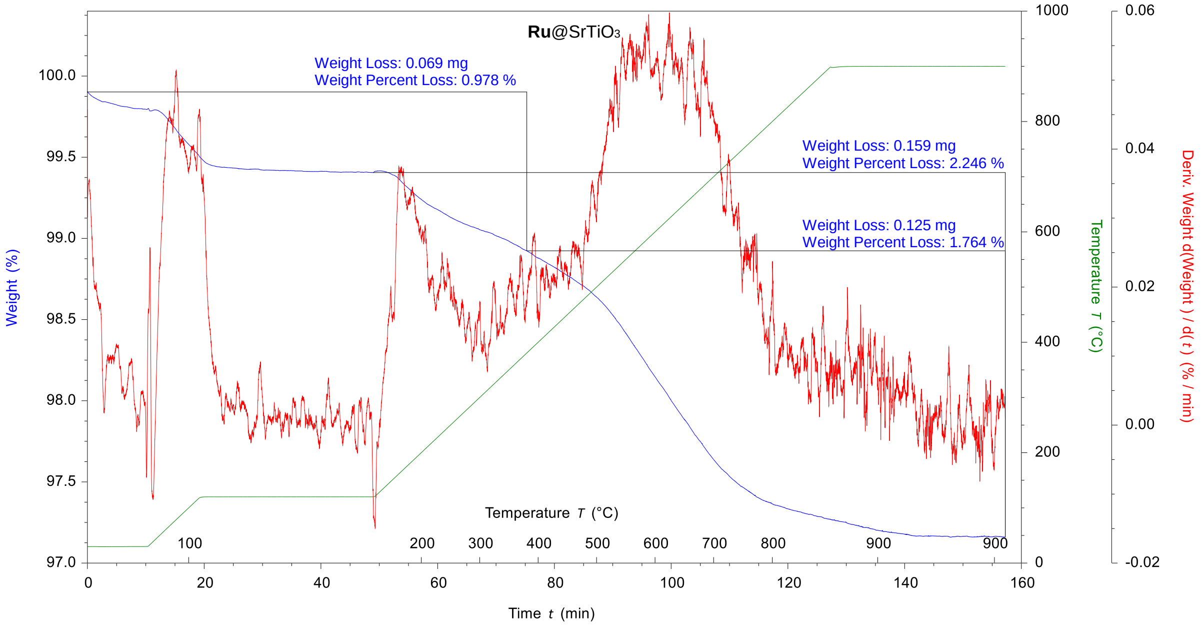

Figure S29. TGA-MS curves for with ligand **1** functionalized and ruthenium and bpy complexed $SrTiO_3$ NPs; **Ru**@$SrTiO_3$ where blue is the weight loss, green is the temperature and red is the derivative weight against time.

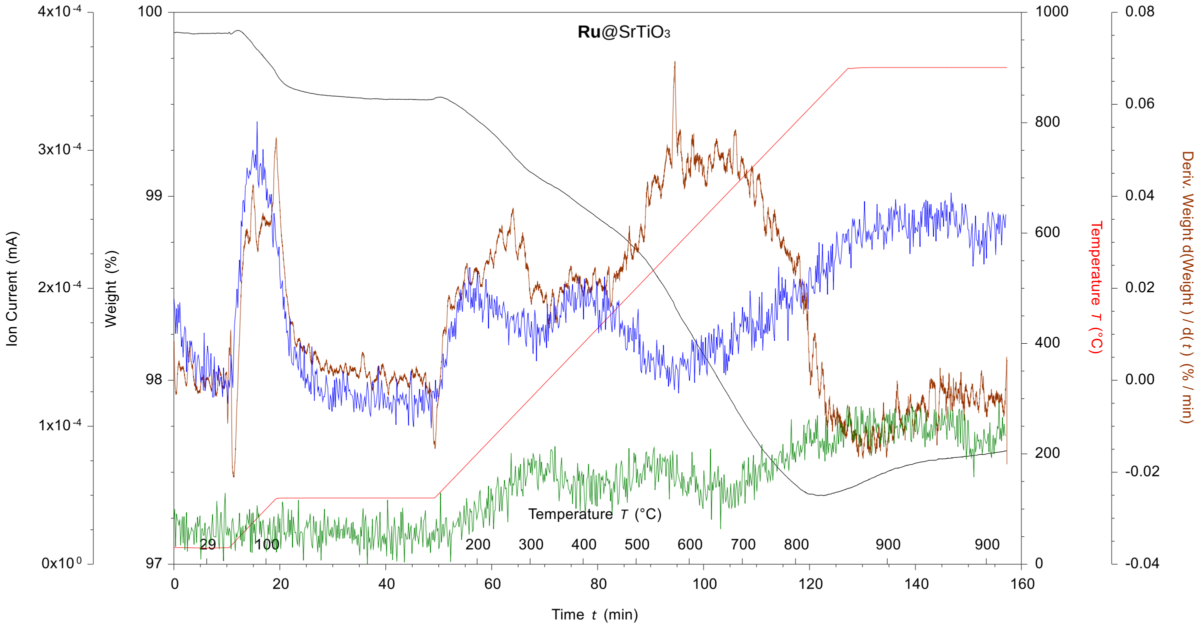

Figure S30. TGA-MS curves for with ligand **1** functionalized and ruthenium and bpy complexed $SrTiO_3$ NPs; **Ru**@$SrTiO_3$ where black is the weight loss, red is the temperature, brown is the derivative weight against time, blue is the ion current of amu 18 and green is the ion current of amu 44.

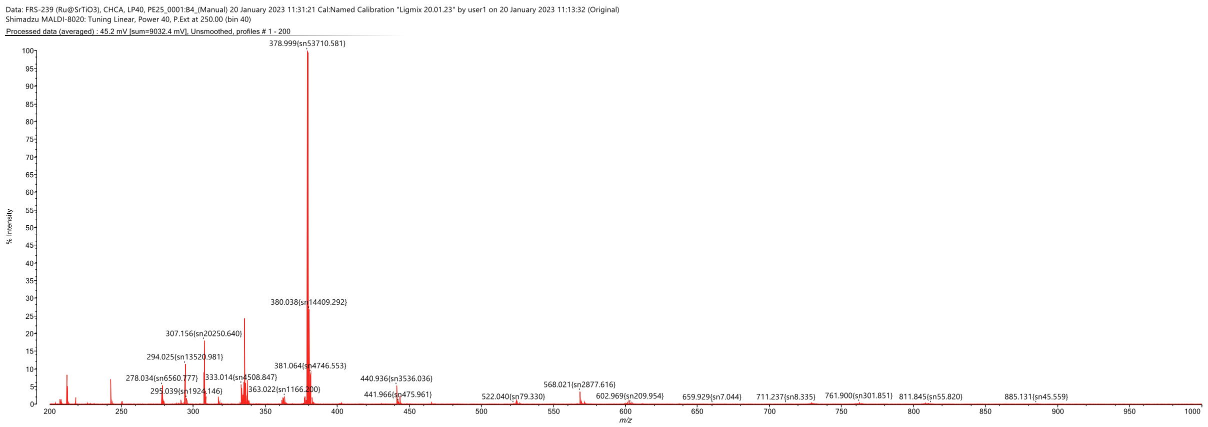

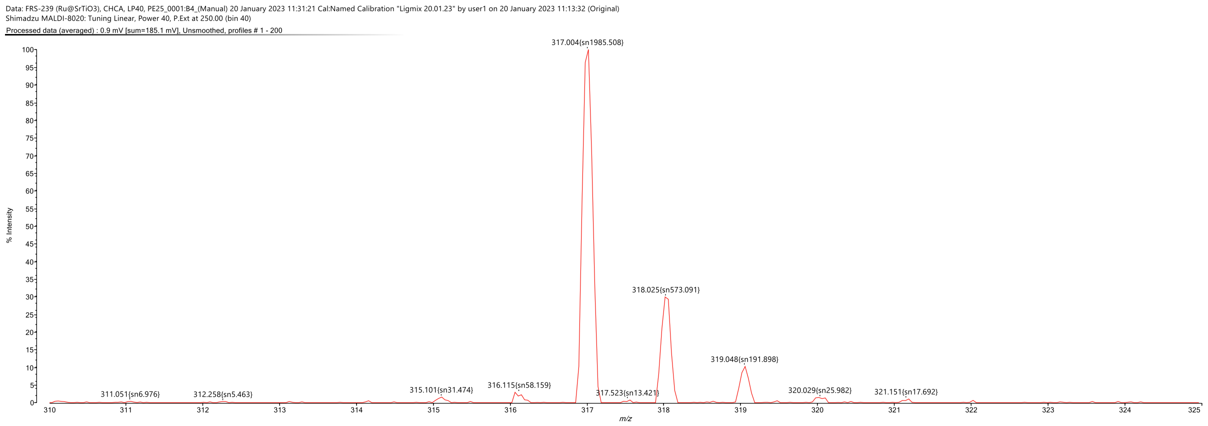

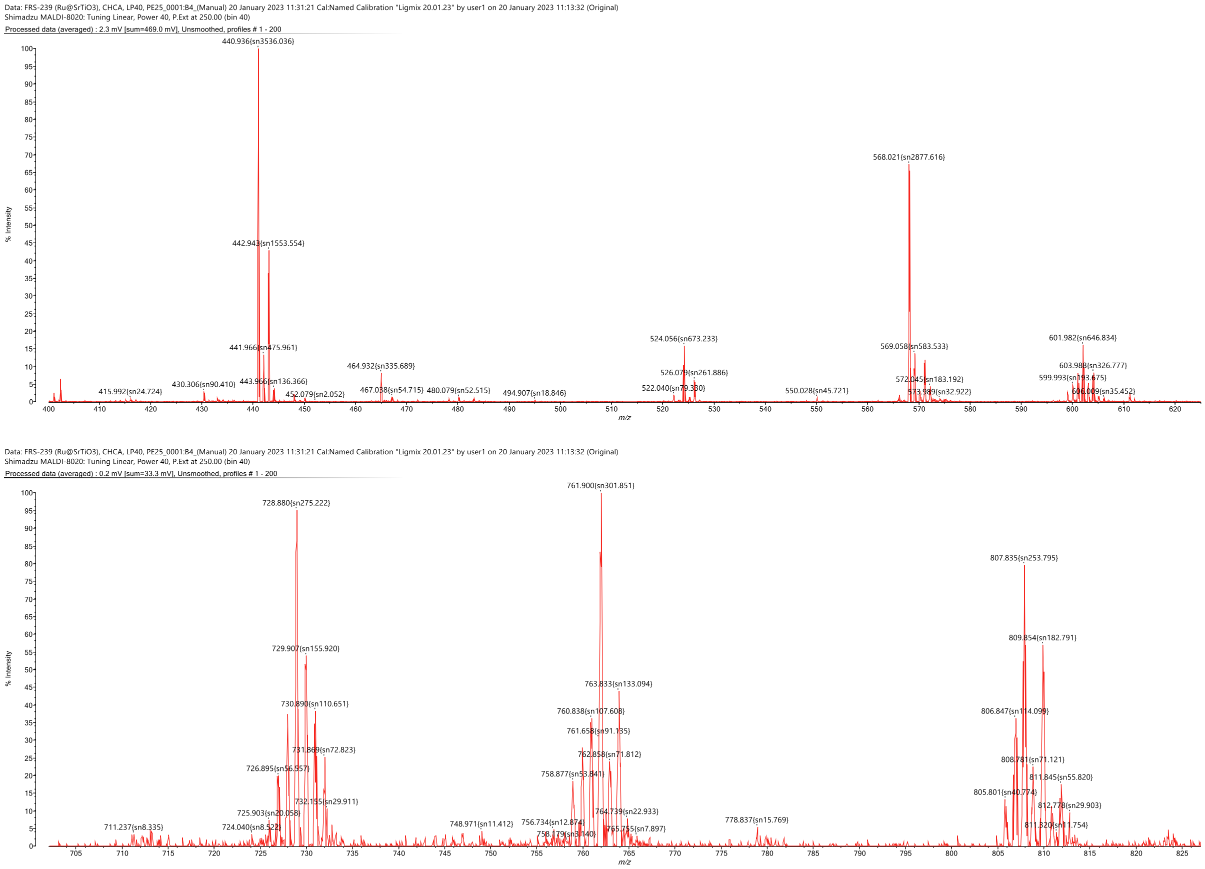

Figure S31. MALDI mass spectrum (with CHCA matrix) of **Ru**@$SrTiO_3$.

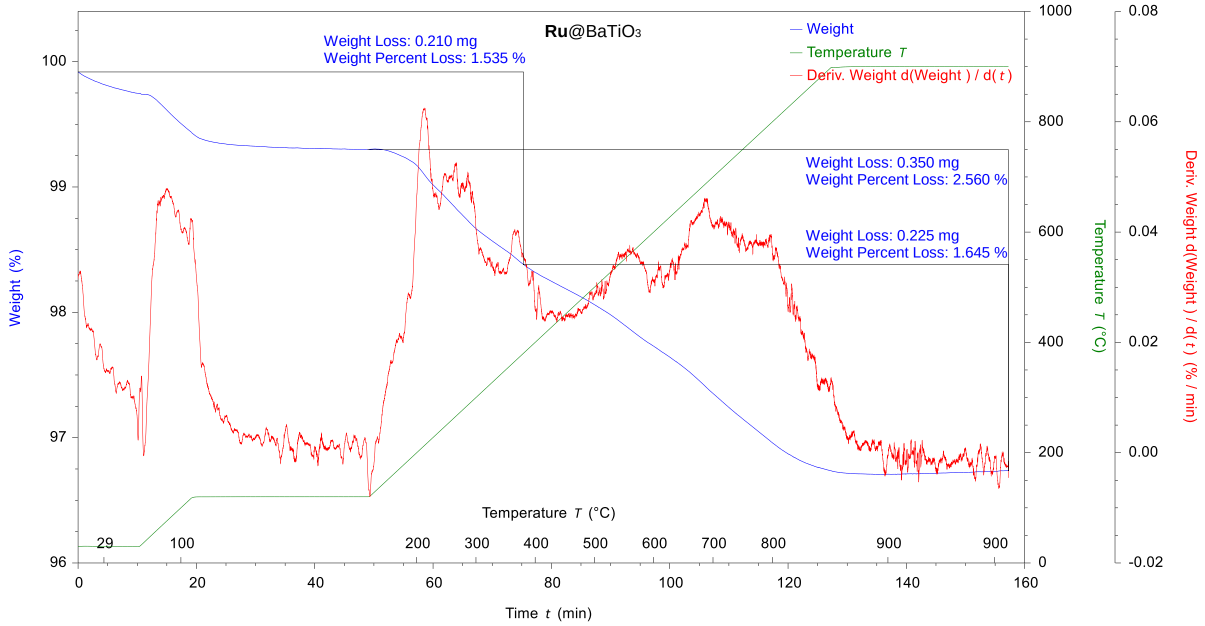

Figure S32. TGA-MS curves for with ligand **1** functionalized and ruthenium and bpy complexed $BaTiO_3$ NPs; **Ru**@$BaTiO_3$ where blue is the weight loss, green is the temperature and red is the derivative weight against time.

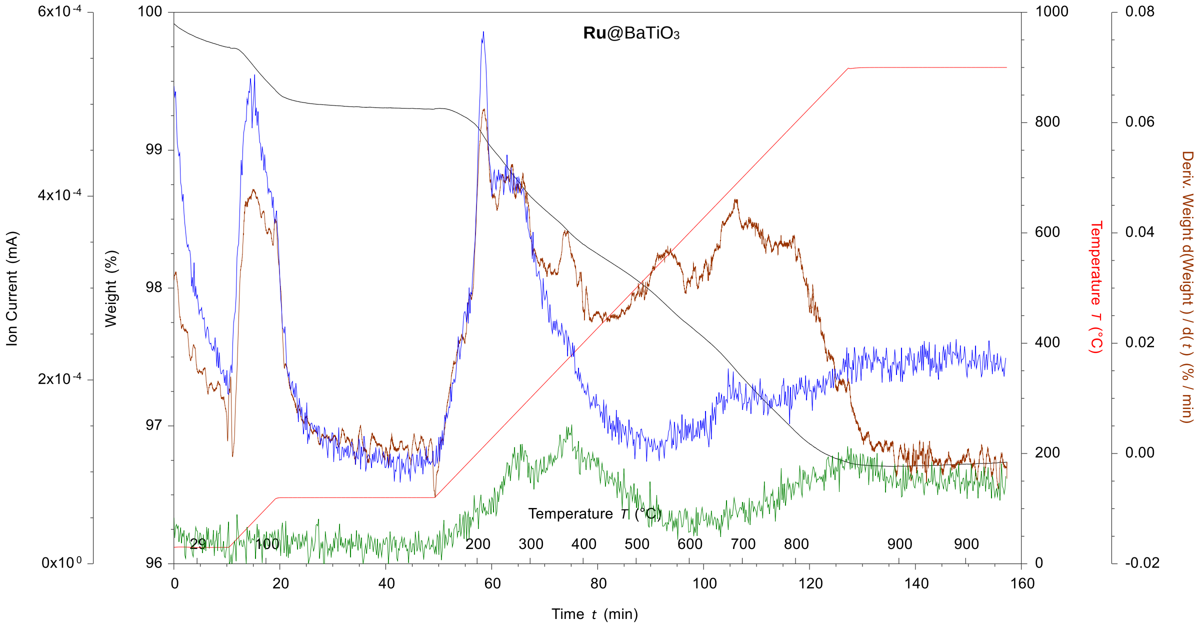

Figure S33. TGA-MS curves for with ligand **1** functionalized and ruthenium and bpy complexed $BaTiO_3$ NPs; **Ru**@$BaTiO_3$ where black is the weight loss, red is the temperature, brown is the derivative weight against time, blue is the ion current of amu 18 and green is the ion current of amu 44.

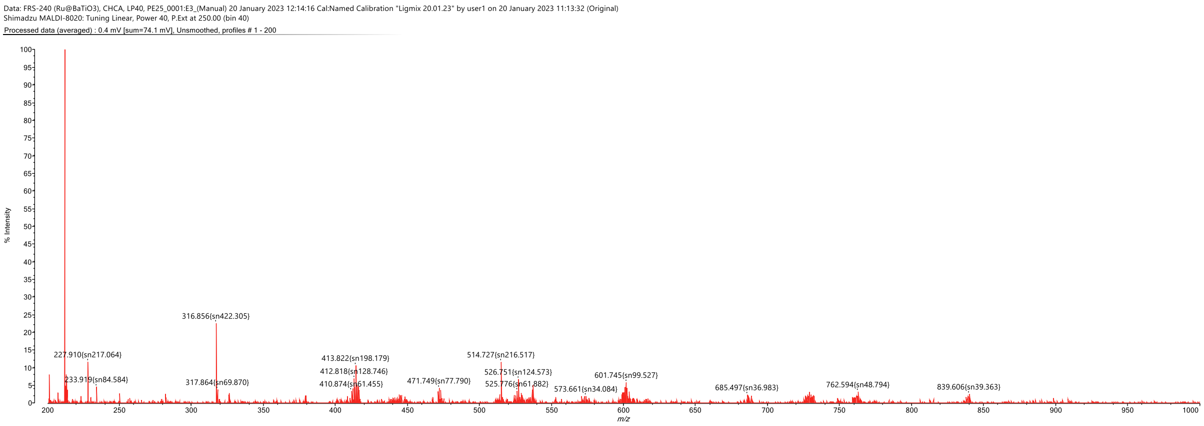

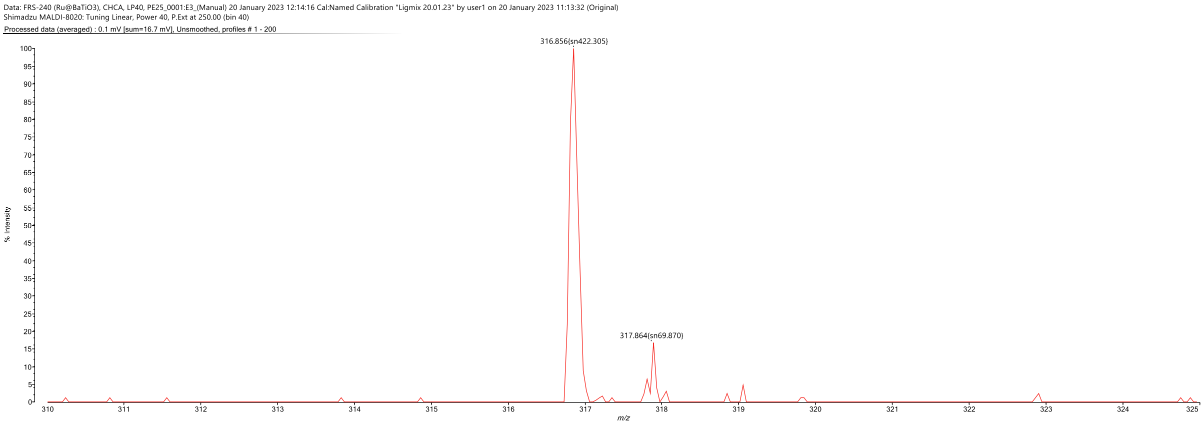

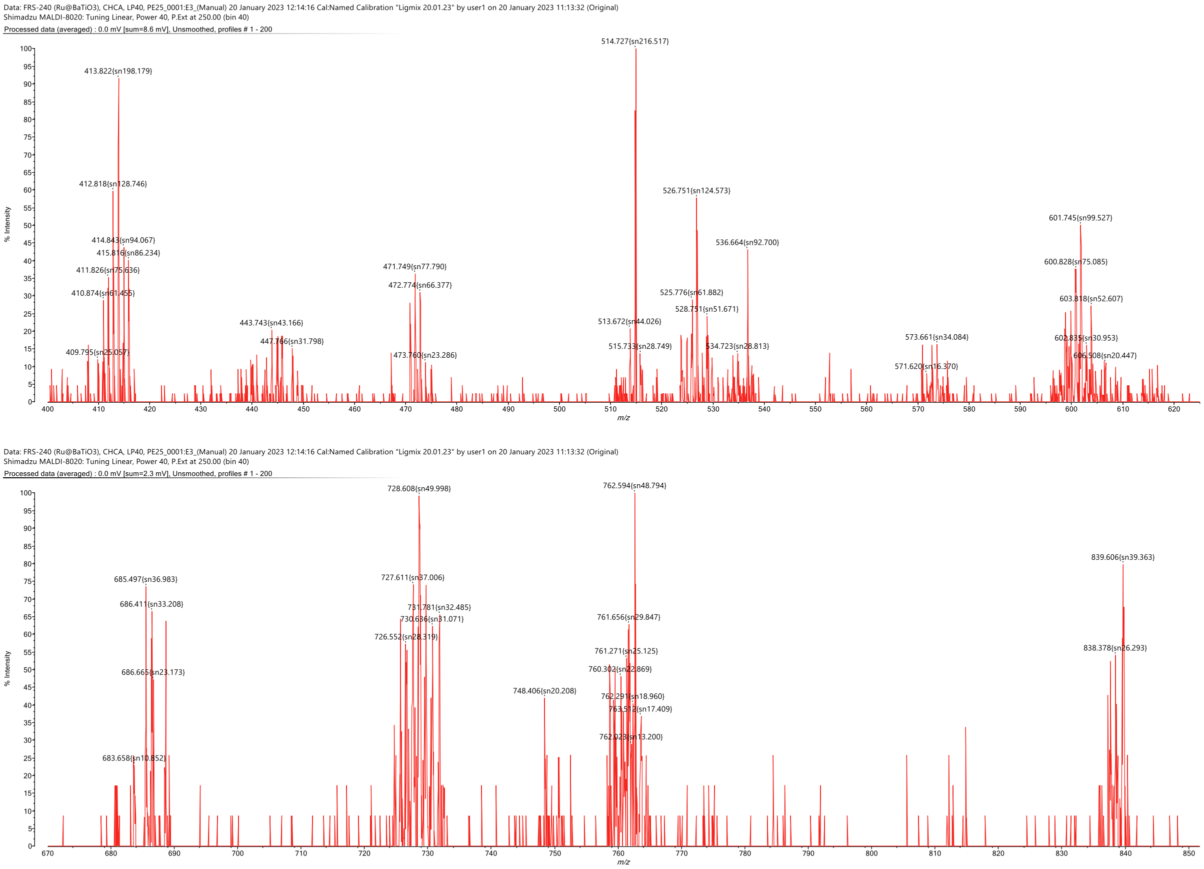

Figure S34. MALDI mass spectrum (with CHCA matrix) of **Ru**@$BaTiO_3$.

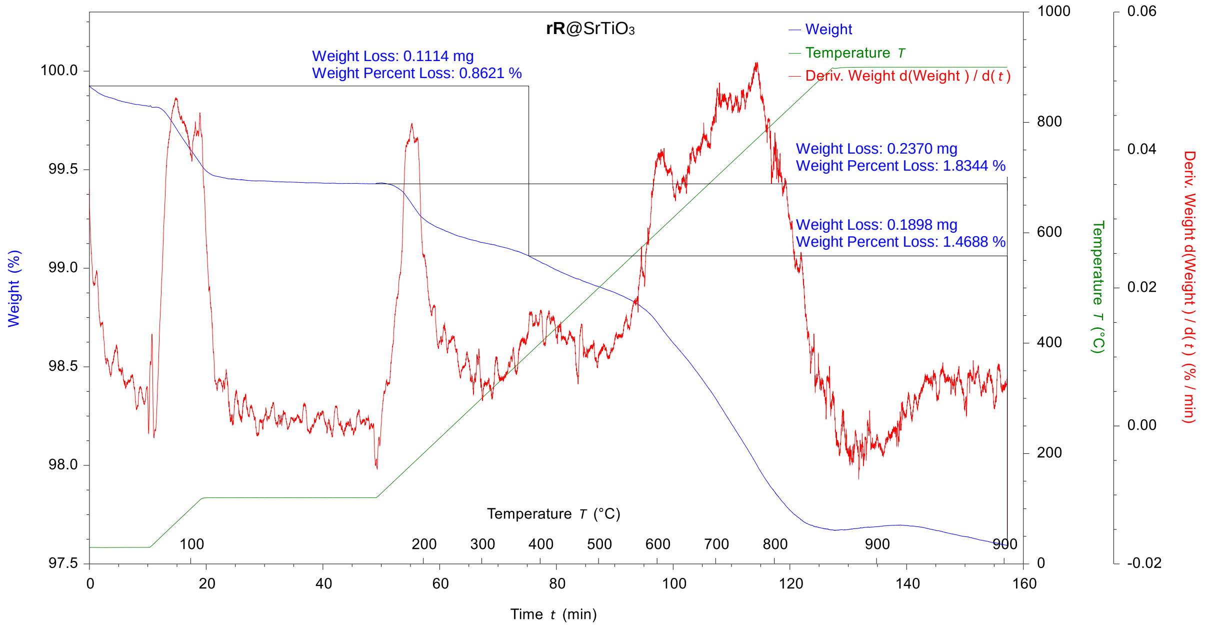

Figure S35. TGA-MS curves for with ligand **1** functionalized and ruthenium, rhodium and bpy complexed $SrTiO_3$ NPs; **rR**@$SrTiO_3$ where blue is the weight loss, green is the temperature and red is the derivative weight against time.

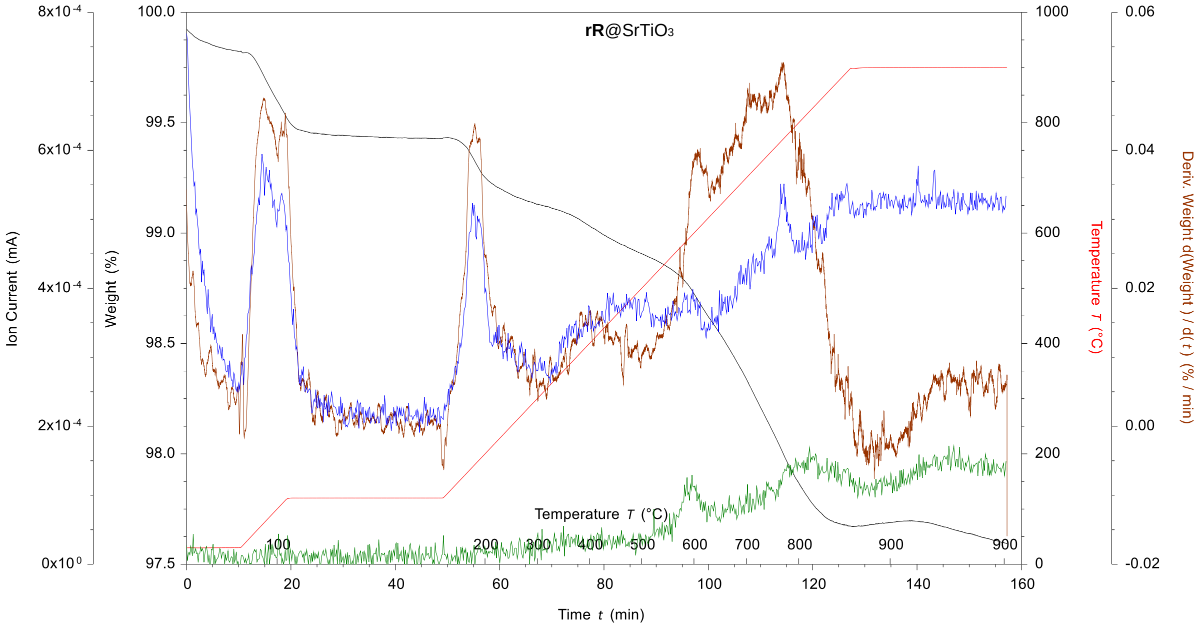

Figure S36. TGA-MS curves for with ligand **1** functionalized and ruthenium, rhodium and bpy complexed $SrTiO_3$ NPs; **rR**@$SrTiO_3$ where black is the weight loss, red is the temperature, brown is the derivative weight against time, blue is the ion current of amu 18 and green is the ion current of amu 44.

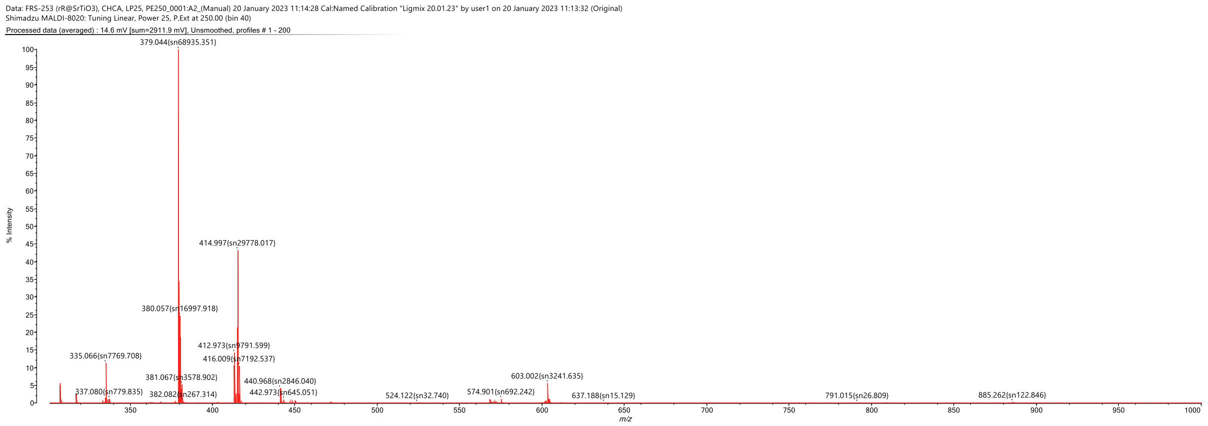

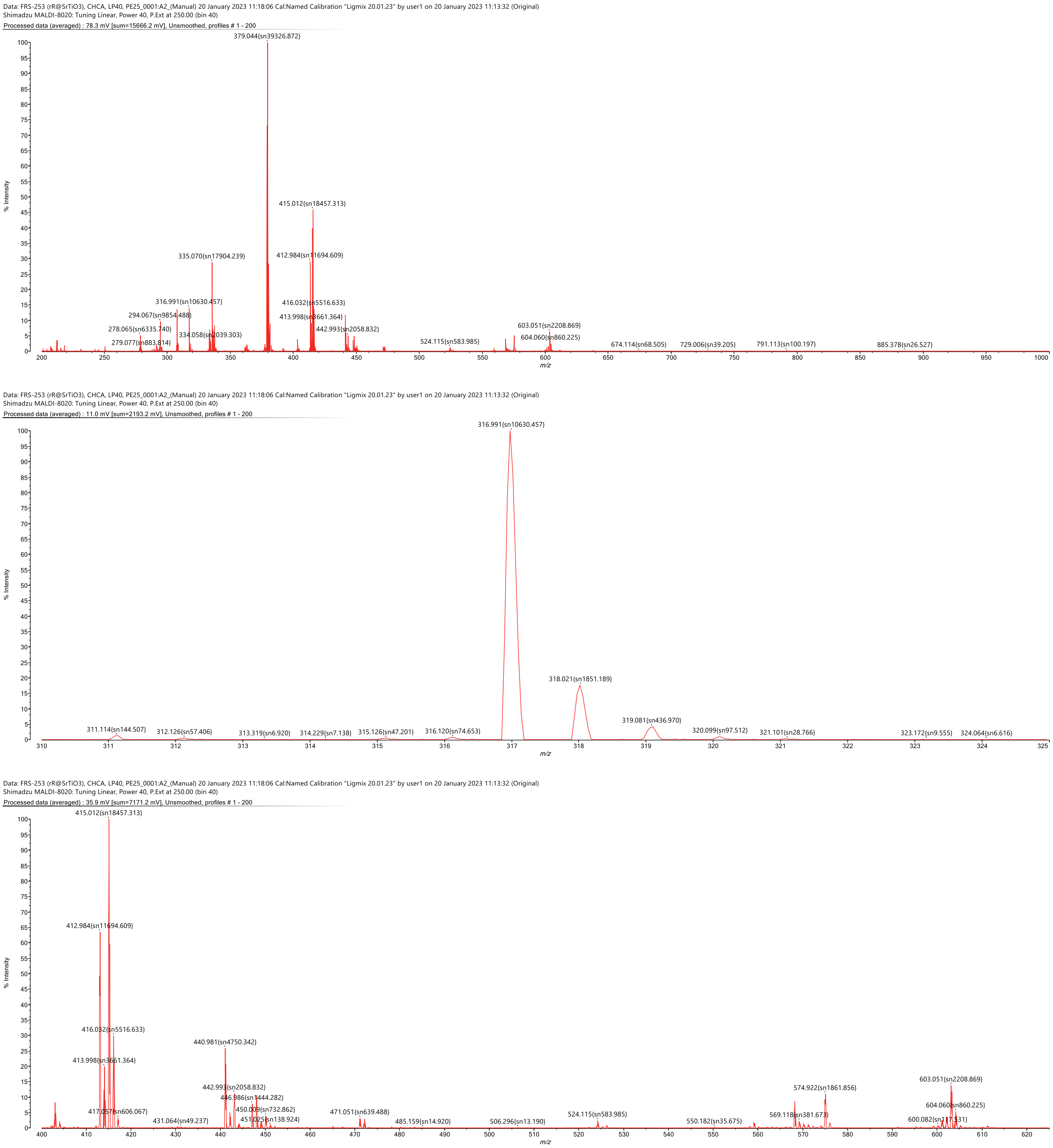

Figure S37. MALDI mass spectrum (with CHCA matrix) of **rR**@$SrTiO_3$.

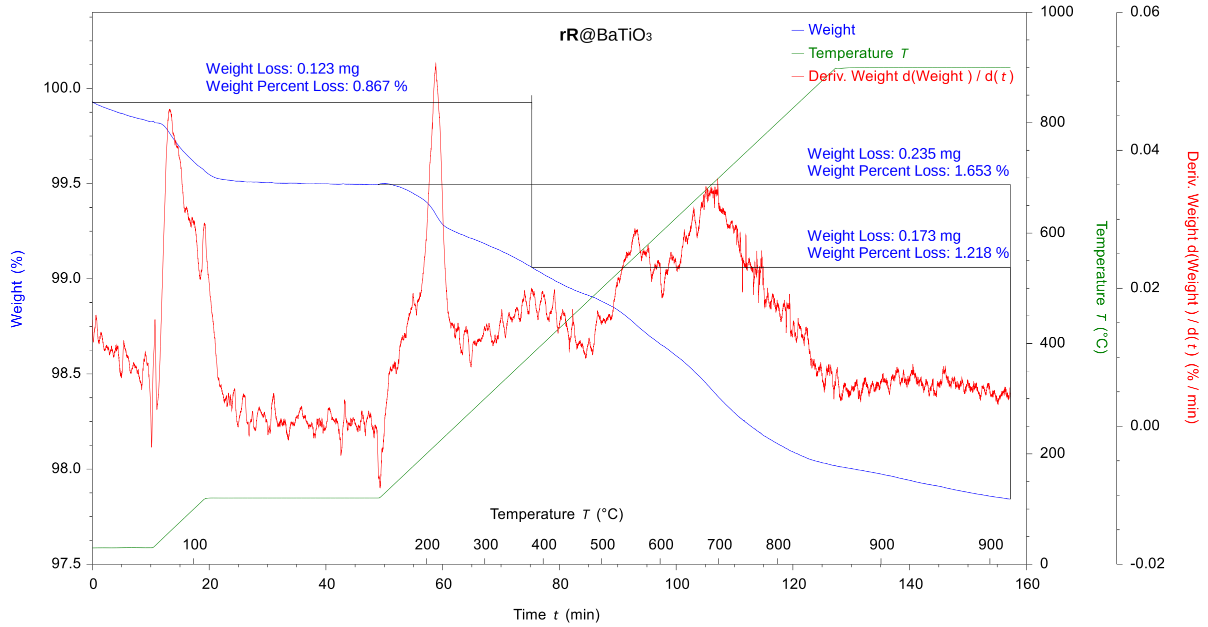

Figure S38. TGA-MS curves for with ligand **1** functionalized and ruthenium, rhodium and bpy complexed $BaTiO_3$ NPs; **rR**@$BaTiO_3$ where blue is the weight loss, green is the temperature and red is the derivative weight against time.

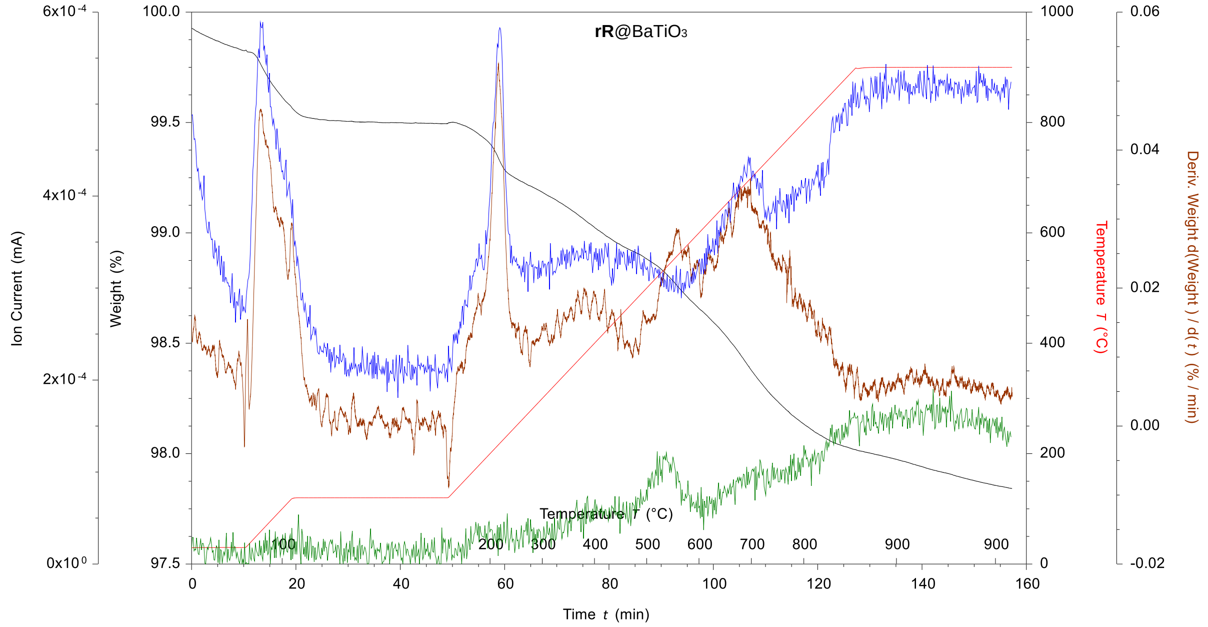

Figure S39. TGA-MS curves for with ligand **1** functionalized and ruthenium, rhodium and bpy complexed $BaTiO_3$ NPs; **rR**@$BaTiO_3$ where black is the weight loss, red is the temperature, brown is the derivative weight against time, blue is the ion current of amu 18 and green is the ion current of amu 44.

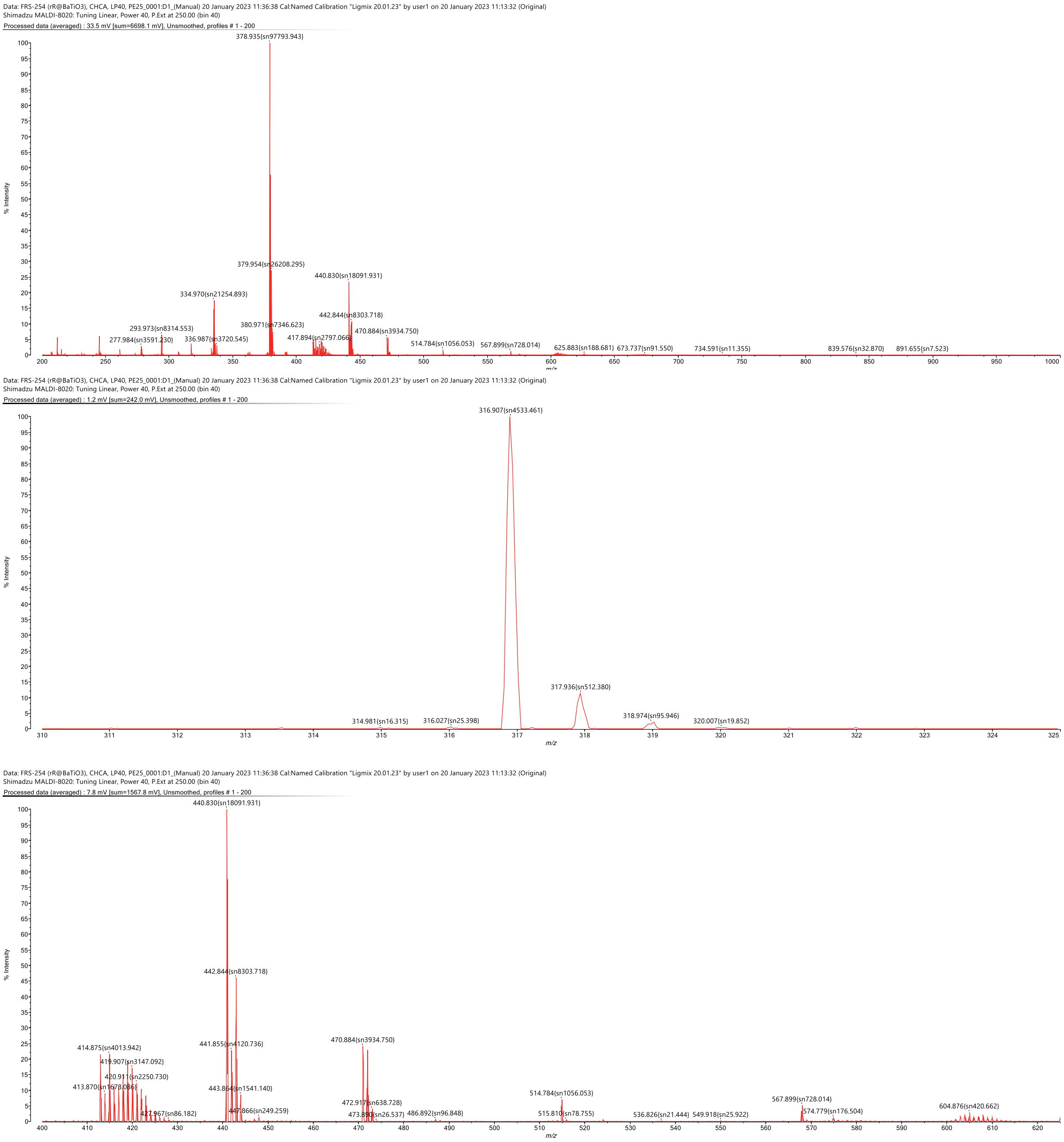

Figure S40. MALDI mass spectrum (with CHCA matrix) of **rR**@$BaTiO_3$.

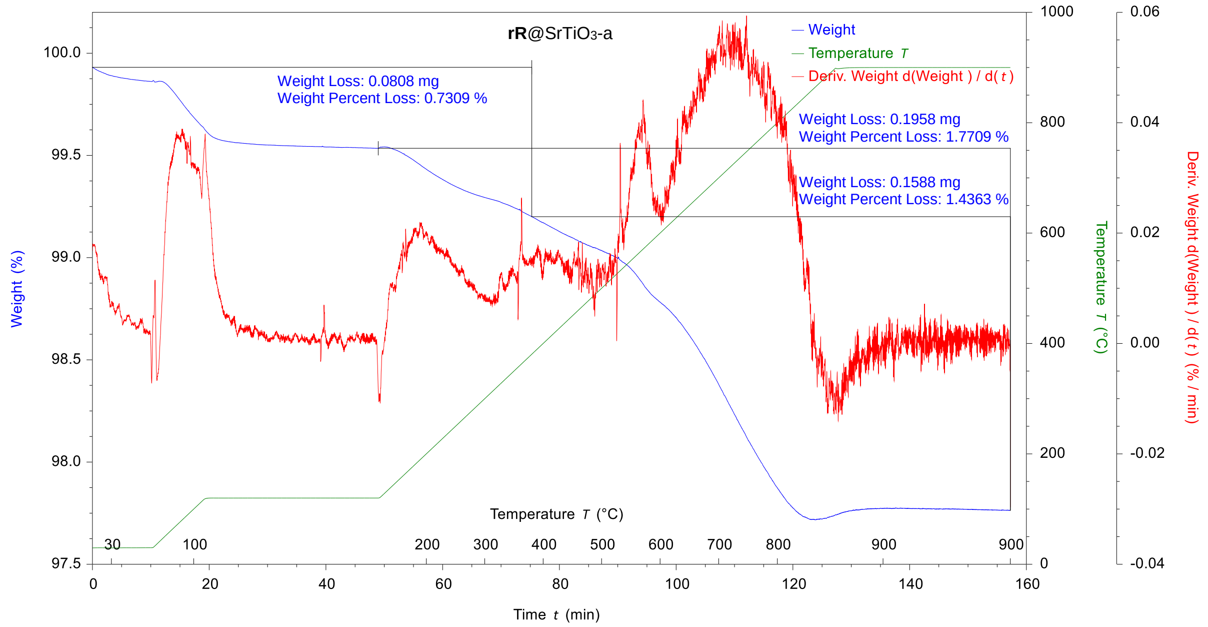

Figure S41. TGA curves for acid activated with ligand **1** functionalized and ruthenium, rhodium and bpy complexed $SrTiO_3$ NPs; **rR**@$SrTiO_3$-a where blue is the weight loss, green is the temperature and red.

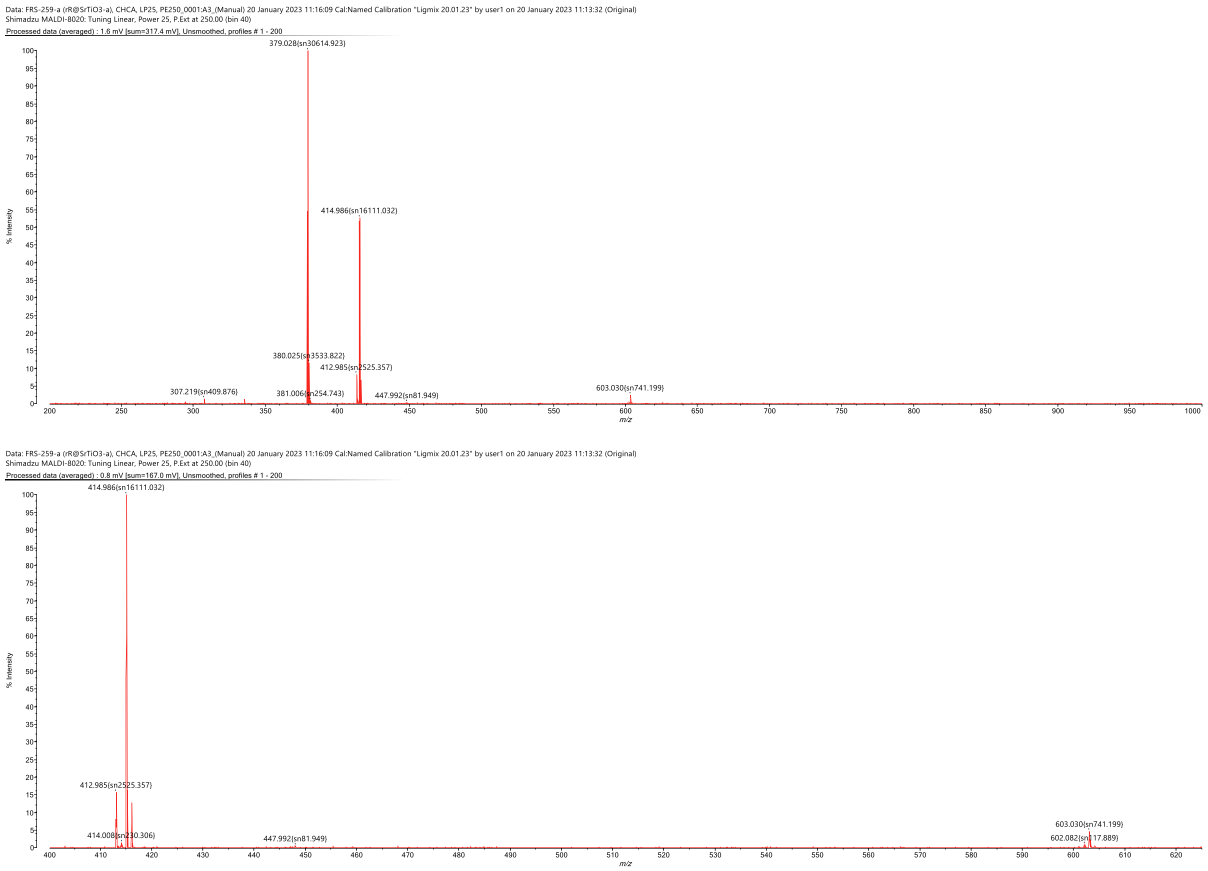

Figure S42. MALDI mass spectrum (with CHCA matrix) of **rR**@$SrTiO_3$-a.

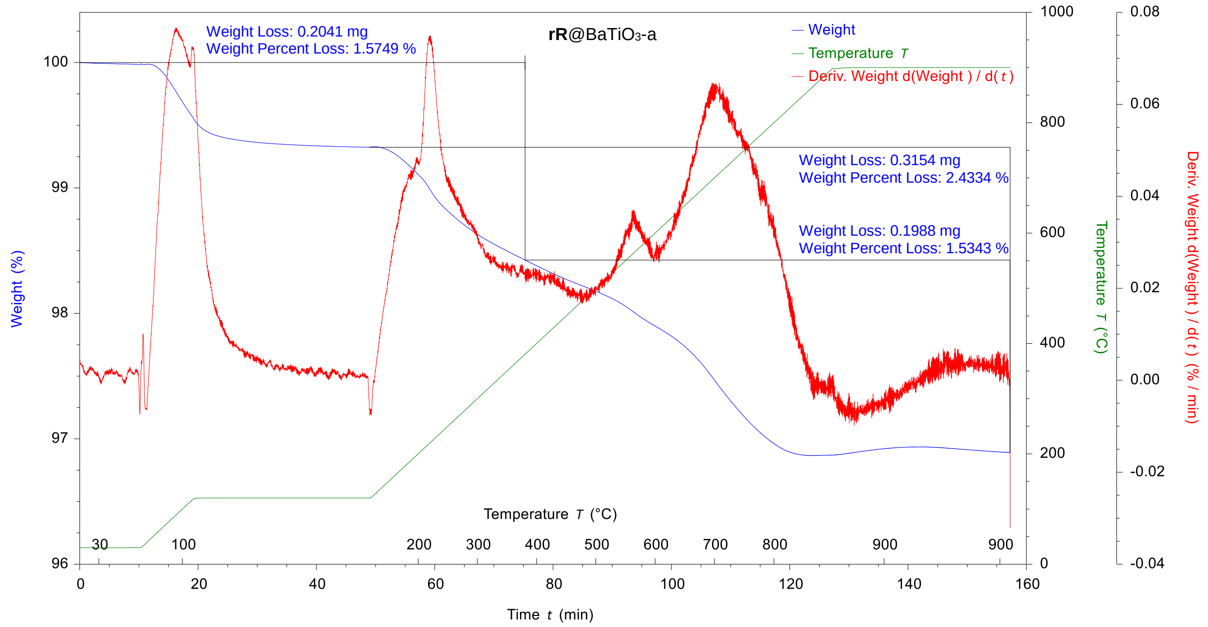

Figure S43. TGA-MS curves for acid activated with ligand **1** functionalized and ruthenium, rhodium and bpy complexed BaTiO3 NPs; **rR**@BaTiO3-a where blue is the weight loss, green is the temperature and red.

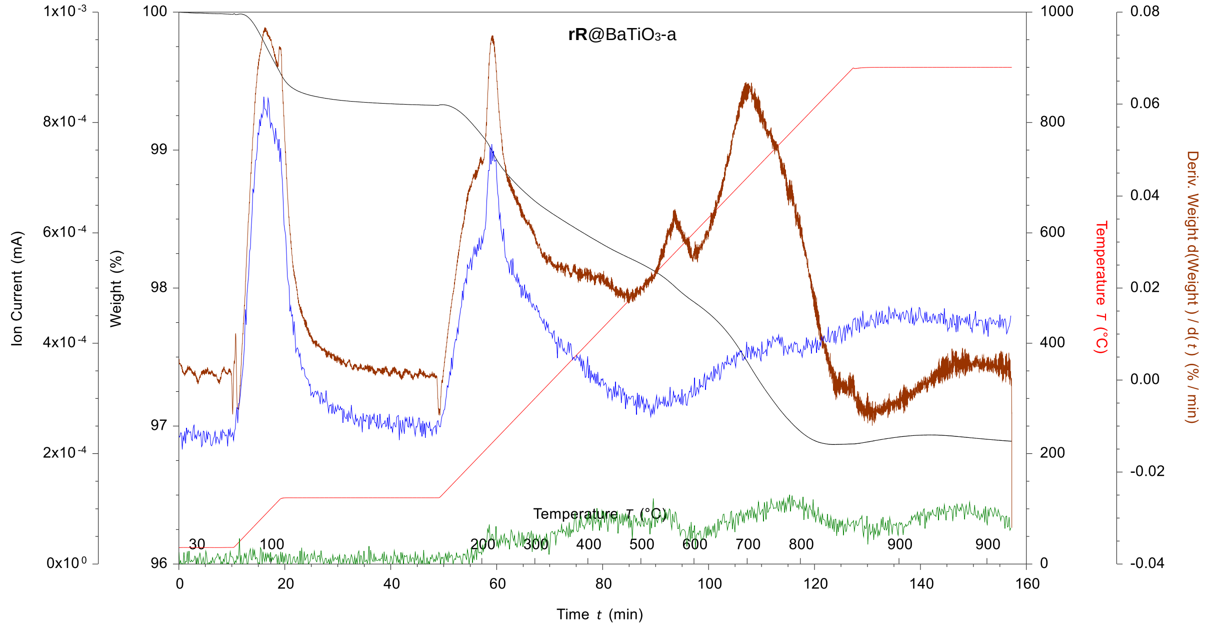

Figure S44. TGA-MS curves for acid activated with ligand **1** functionalized and ruthenium, rhodium and bpy complexed BaTiO3 NPs; **rR**@BaTiO3-a where black is the weight loss, red is the temperature, brown is the derivative weight against time, blue is the ion current of amu 18 and green is the ion current of amu 44.

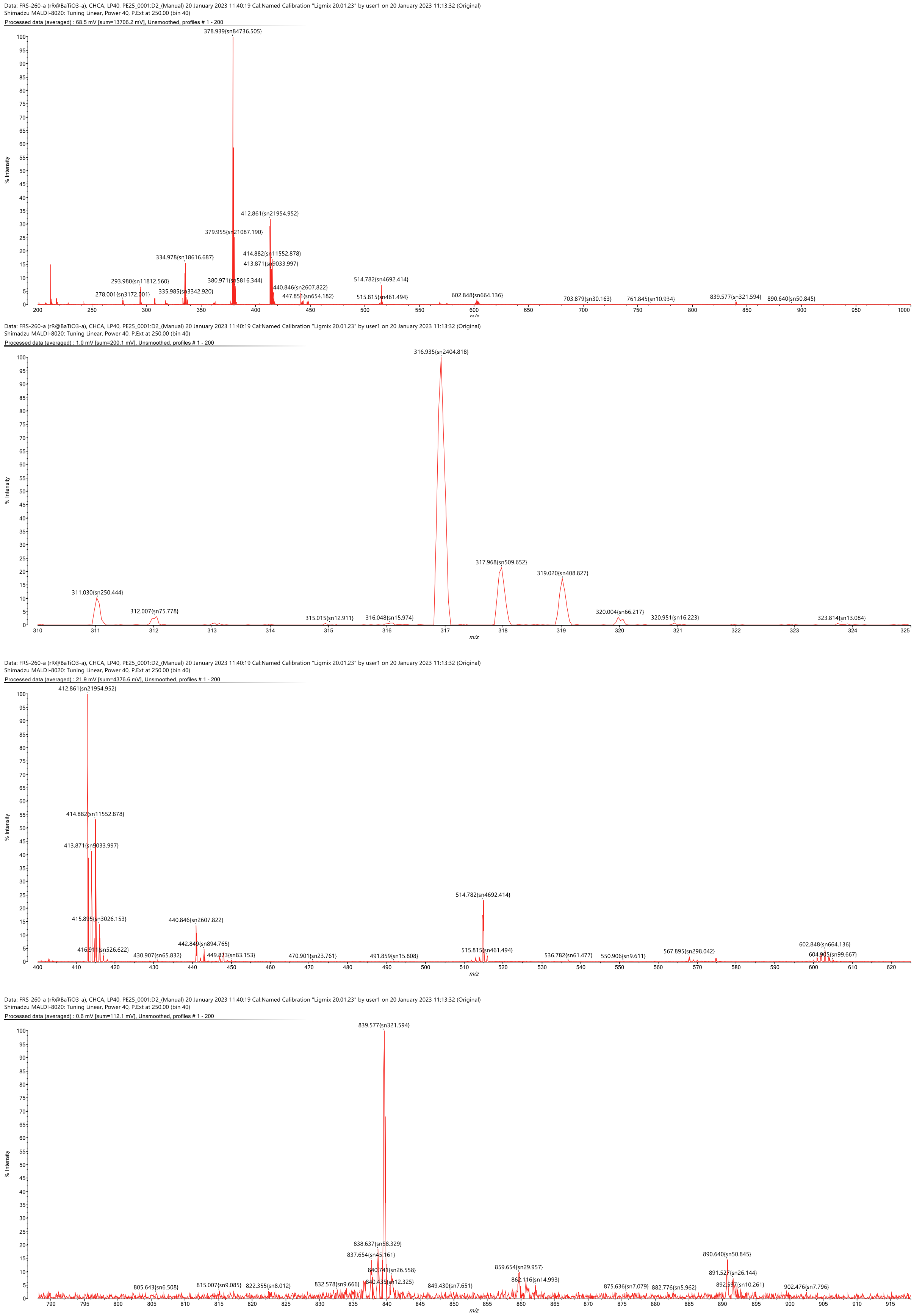

Figure S45. MALDI mass spectrum (with CHCA matrix) of **rR**@$BaTiO_3$-a.

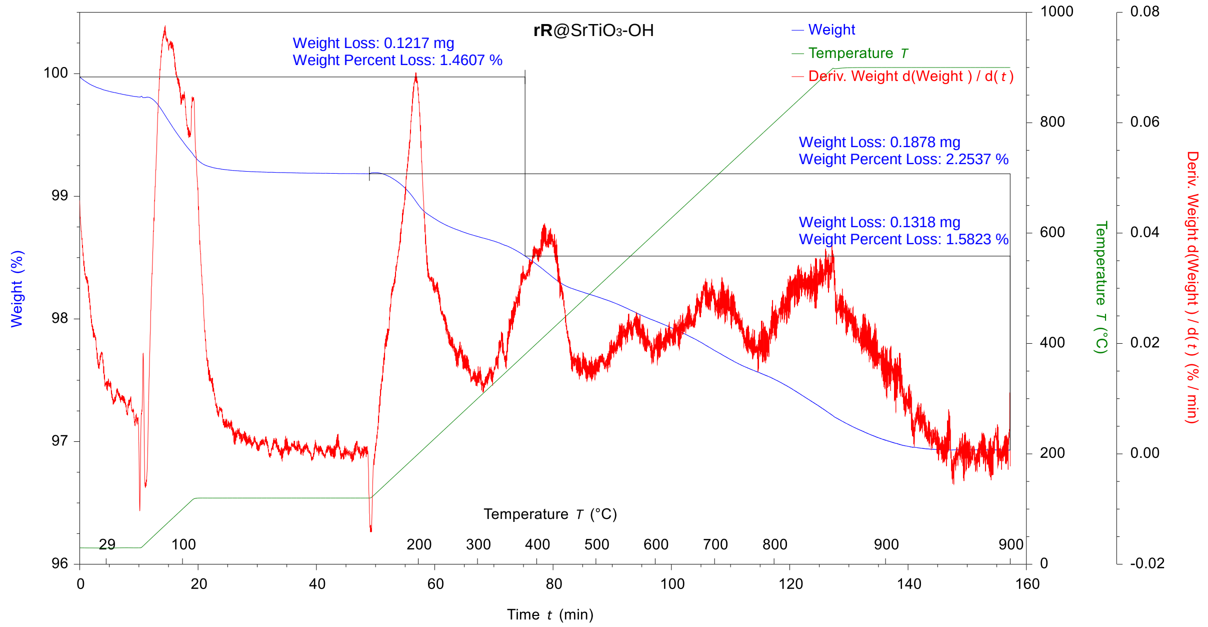

Figure S46. TGA-MS curves for H_2O_2 activated with ligand **1** functionalized and ruthenium, rhodium and bpy complexed $SrTiO_3$ NPs; **rR**@$SrTiO_3$-OH where blue is the weight loss, green is the temperature and red.

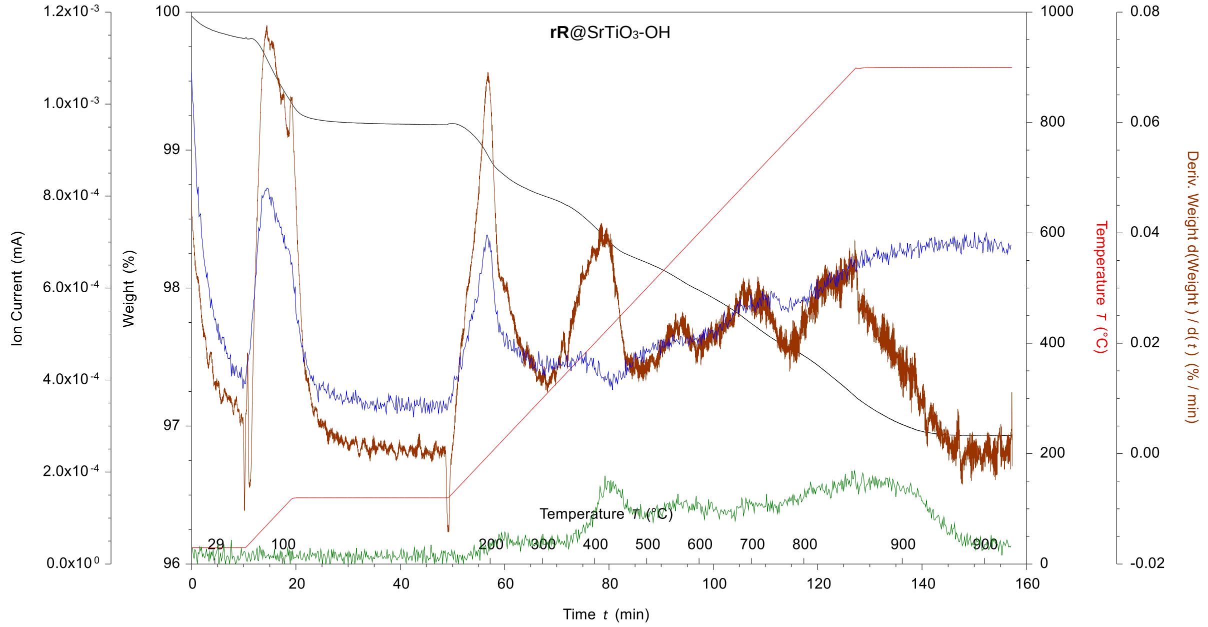

Figure S47. TGA-MS curves for H_2O_2 activated with ligand **1** functionalized and ruthenium, rhodium and bpy complexed $SrTiO_3$ NPs; **rR**@$SrTiO_3$-OH where black is the weight loss, red is the temperature, brown is the derivative weight against time, blue is the ion current of amu 18 and green is the ion current of amu 44.

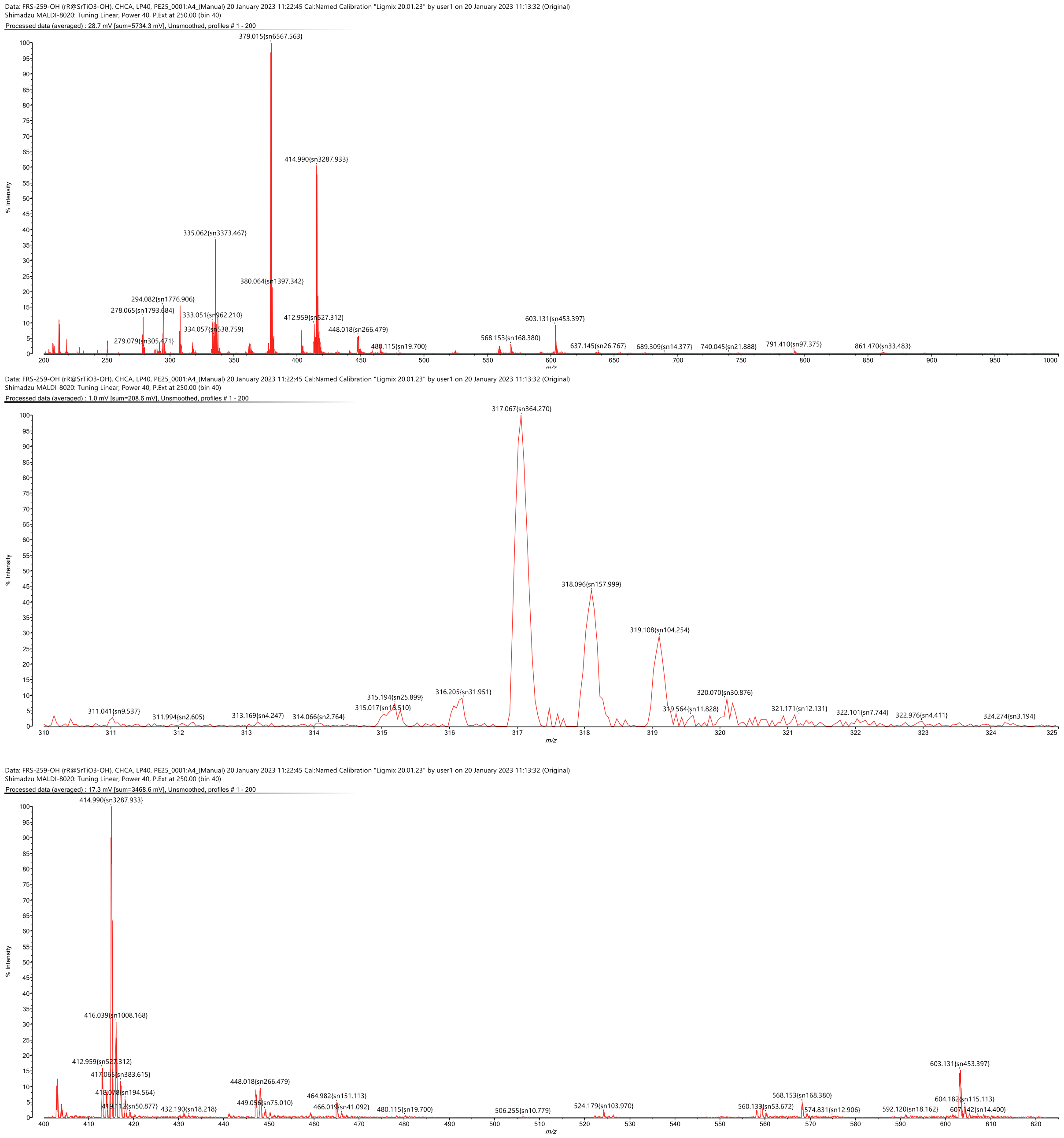

Figure S48. MALDI mass spectrum (with CHCA matrix) of **rR**@SrTiO$_3$-OH.

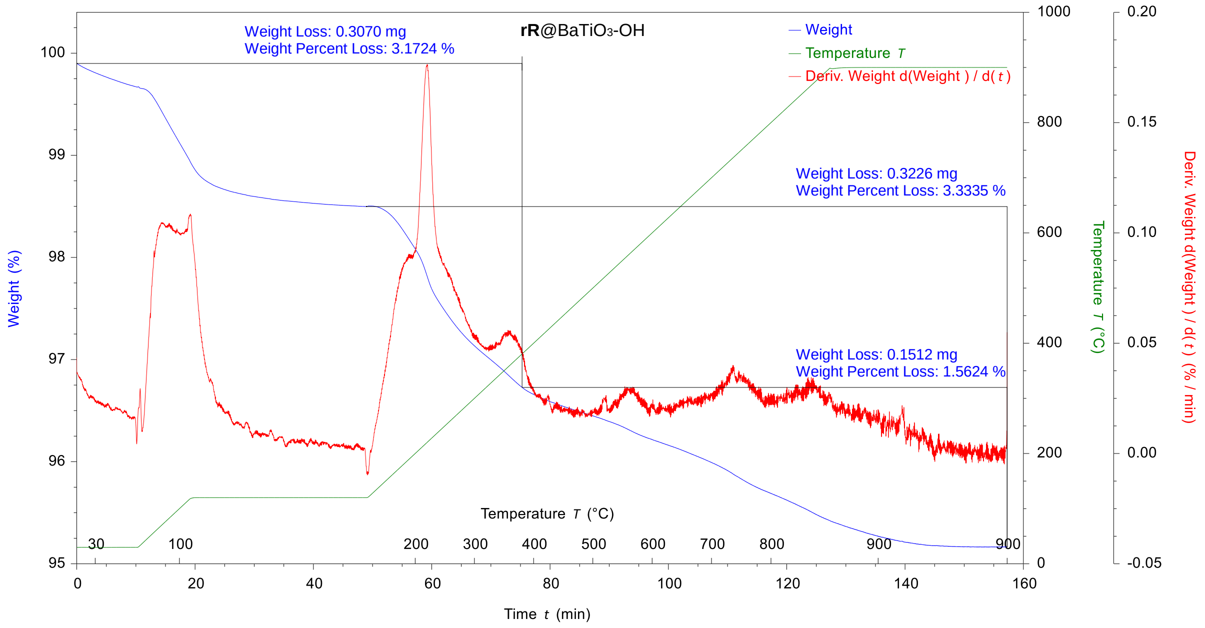

Figure S49. TGA-MS curves for H_2O_2 activated with ligand **1** functionalized and ruthenium, rhodium and bpy complexed $BaTiO_3$ NPs; **rR**@$BaTiO_3$-OH where blue is the weight loss, green is the temperature and red.

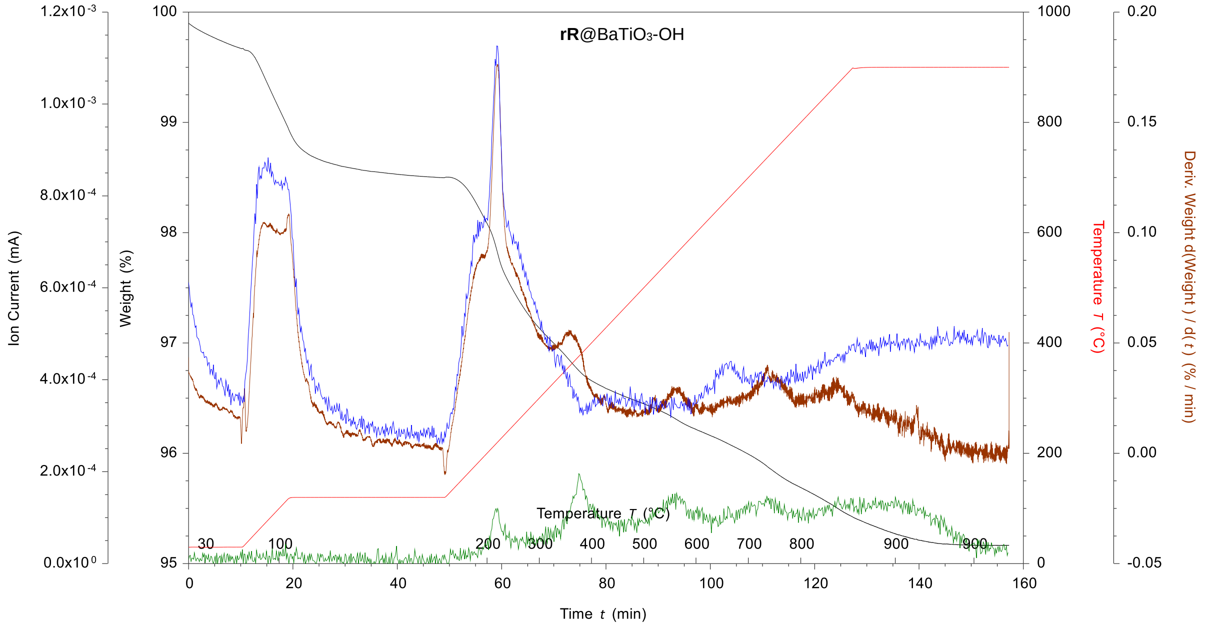

Figure S50. TGA-MS curves for H_2O_2 activated with ligand **1** functionalized and ruthenium, rhodium and bpy complexed $BaTiO_3$ NPs; **rR**@$BaTiO_3$-OH where black is the weight loss, red is the temperature, brown is the derivative weight against time, blue is the ion current of amu 18 and green is the ion current of amu 44.

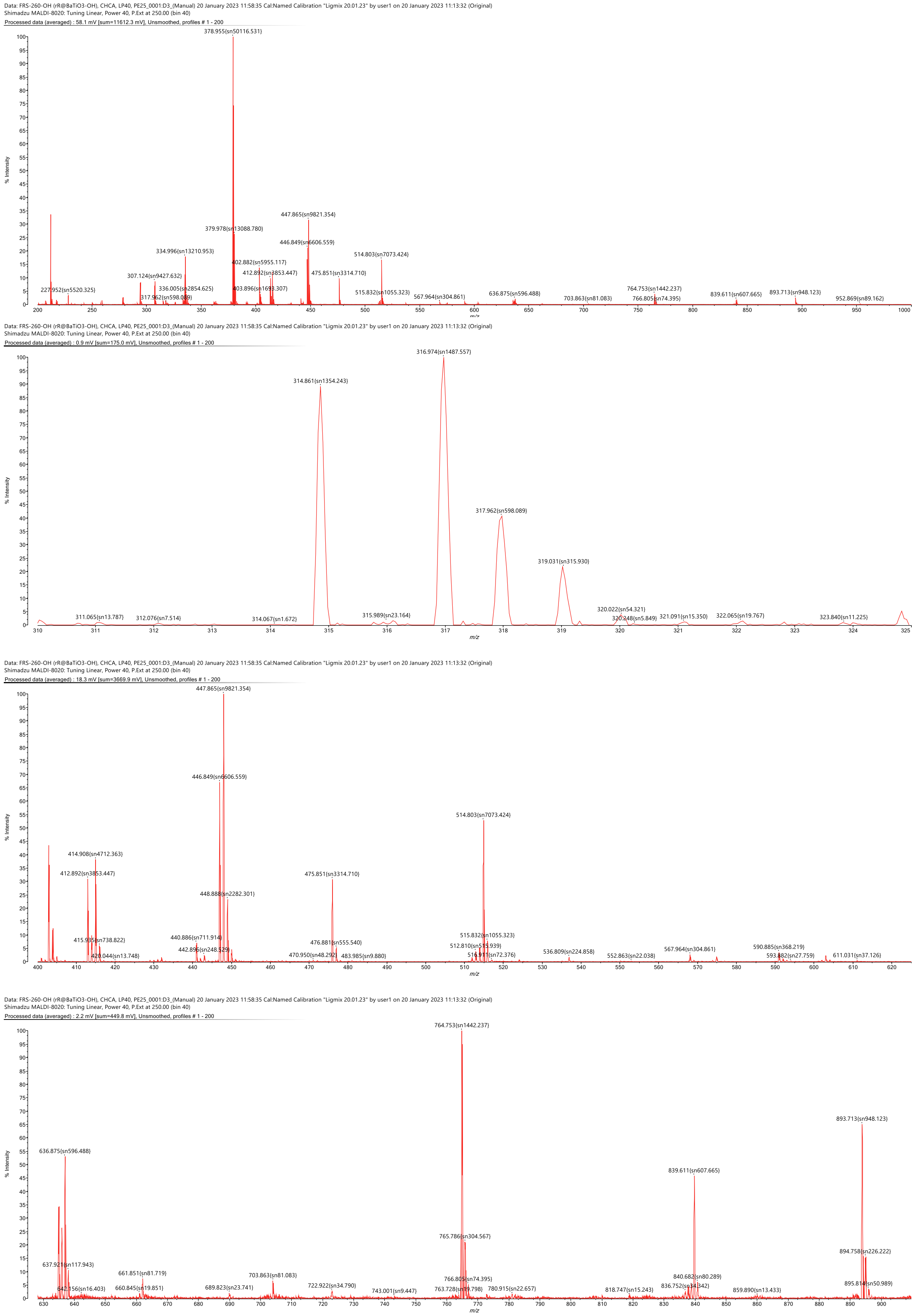

Figure S51. MALDI mass spectrum (with CHCA matrix) of **rR**@$BaTiO_3$-OH.

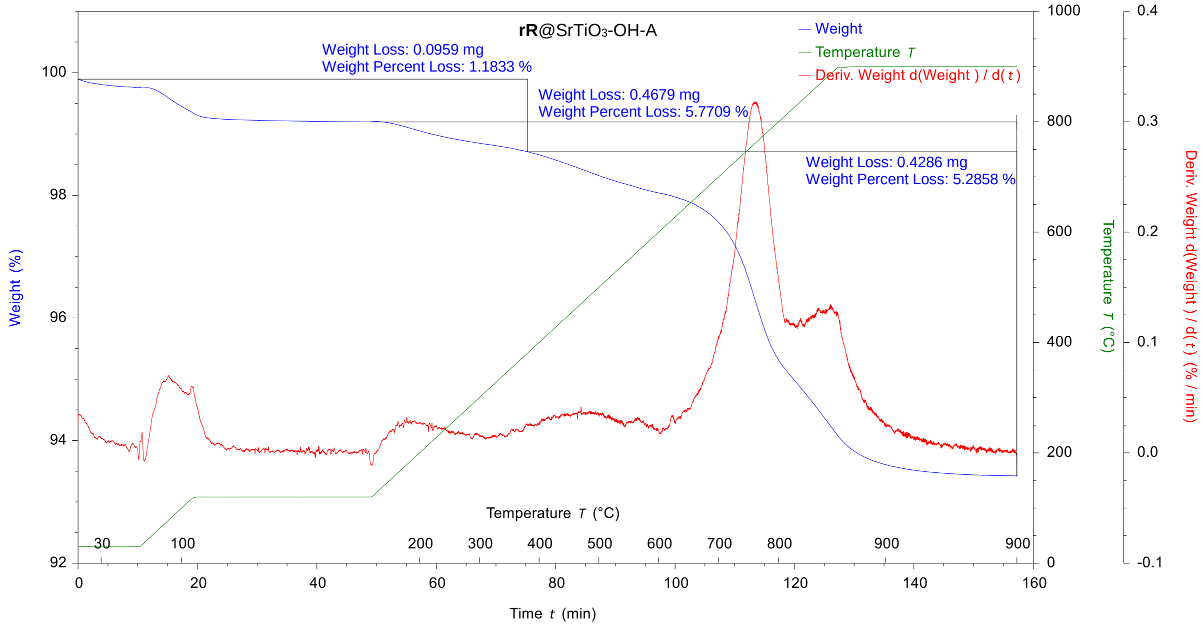

Figure S52. TGA curves for H_2O_2 activated with ligand **1** functionalized and ruthenium, rhodium and bpy under adjusted pH complexed $SrTiO_3$ NPs; **rR**@$SrTiO_3$-OH-A where blue is the weight loss, green is the temperature and red is the derivative weight against time.

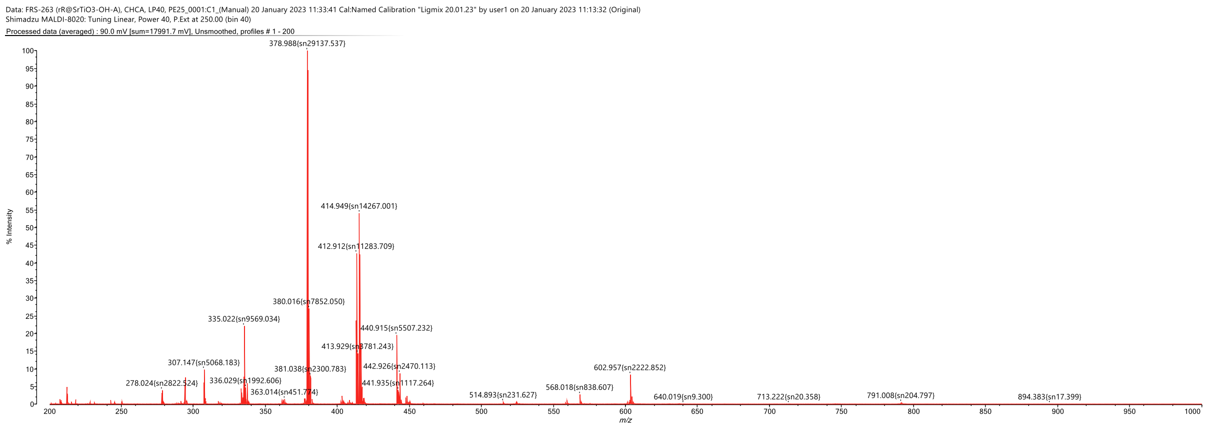

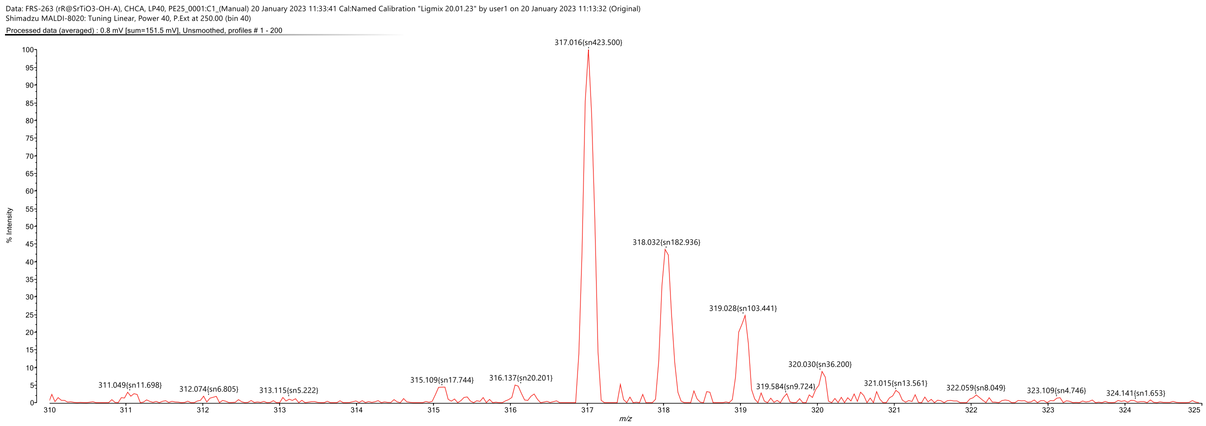

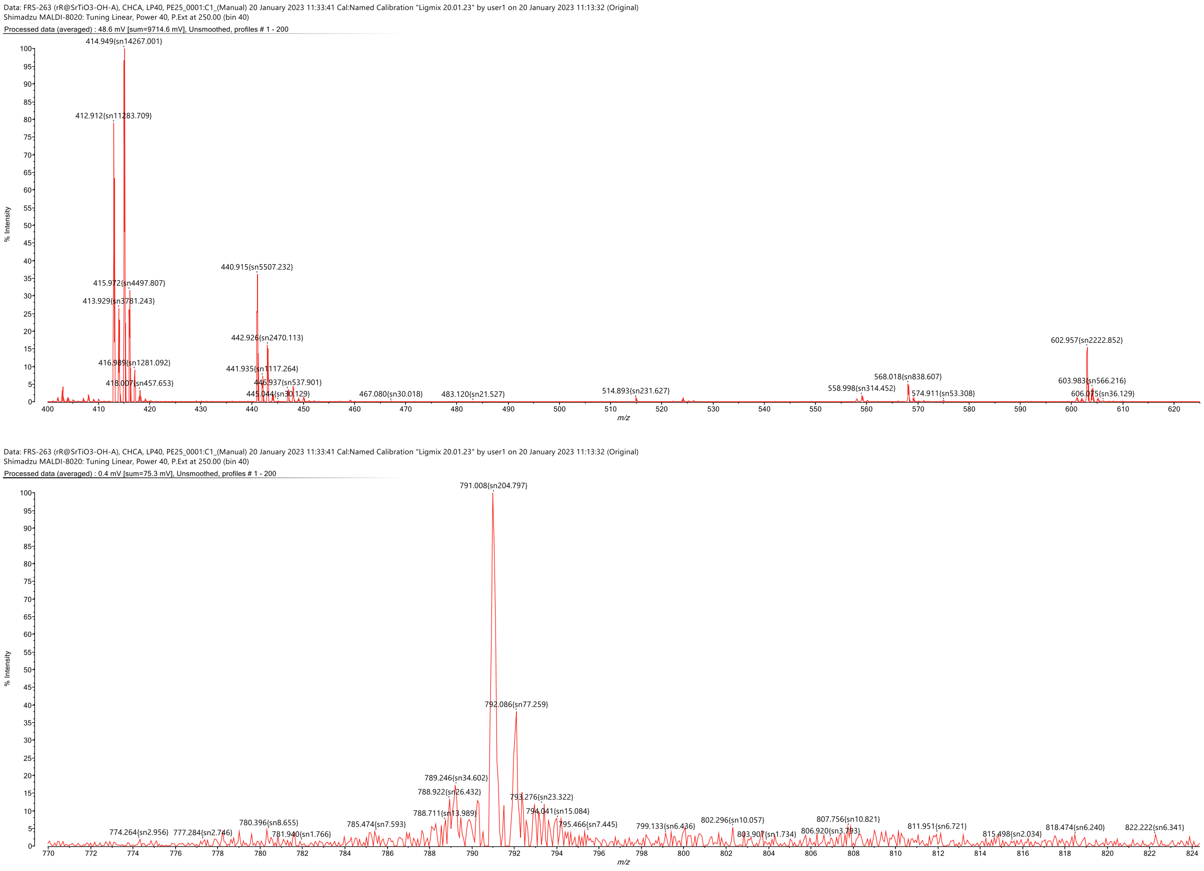

Figure S53. MALDI mass spectrum (with CHCA matrix) of **rR**@$SrTiO_3$-OH-A.

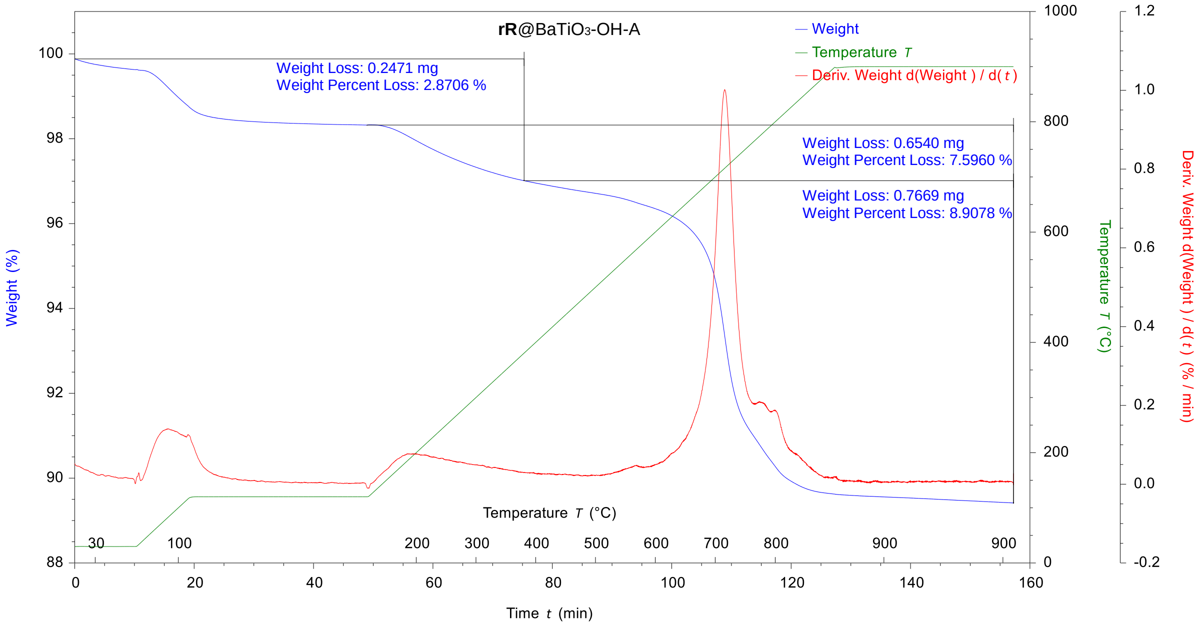

Figure S54. TGA curves for H_2O_2 activated with ligand **1** functionalized and ruthenium, rhodium and bpy under adjusted pH complexed $BaTiO_3$ NPs; **rR**@$BaTiO_3$-OH-A where blue is the weight loss, green is the temperature and red is the derivative weight against time.

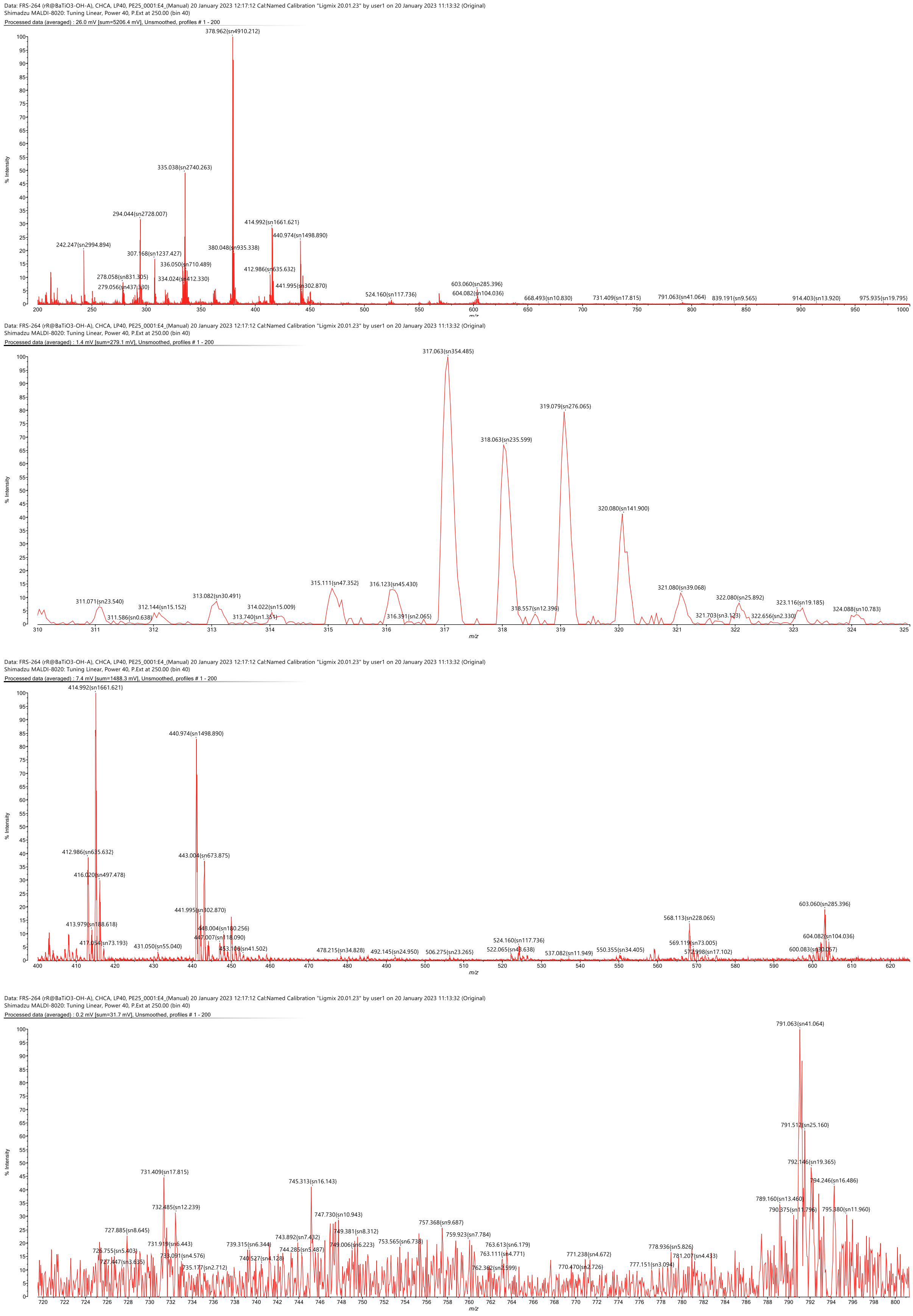

Figure S55. MALDI mass spectrum (with CHCA matrix) of **rR**@$BaTiO_3$-OH-A.

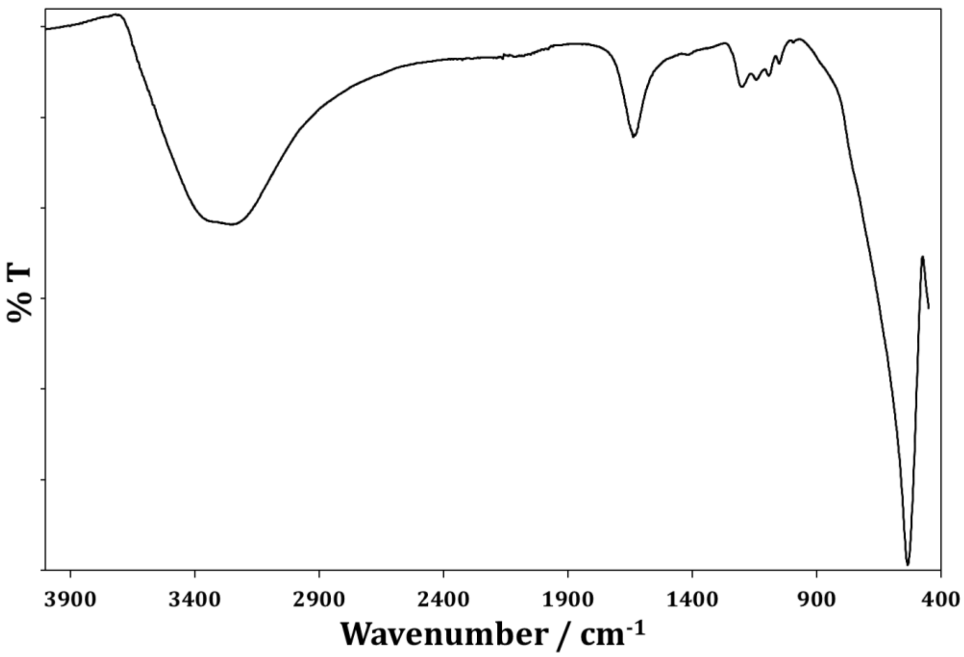

Figure S56. FTIR spectra of commercial $SrTiO_3$ NPs (500 mg) mixed with 200 μL aqueous H_2SO_4 (3M), FTIR spectroscopy: 3243, 1637, 1199, 1141, 1091, 1051 and 534 cm^{-1}.
